# An Energy-Gradient Strategy for Triplet-Pair Separation in Singlet-Fission Oligomers

**DOI:** 10.1021/jacs.6c09940

**Published:** 2026-08-14

**Authors:** Ebin Sebastian, Stephanie Montanaro, Oliver Millington, Ashish Sharma, Anastasia Leventis, Julia Osmólska, Daniel G. Congrave, Akshay Rao, Hugo Bronstein

**Affiliations:** † Cavendish Laboratory, 2152 University of Cambridge, J. J. Thomson Avenue, Cambridge CB3 0HE, U.K.; ‡ Department of Chemistry, University of Cambridge, Lensfield Road, Avenue, Cambridge CB2 1EW, U.K.; § Chemistry Research Laboratory, 6396 University of Oxford, Oxford OX1 3TA, U.K.

## Abstract

Singlet fission (SF) offers a pathway to surpass the Shockley–Queisser limit by converting one photoexcited singlet exciton into two triplet excitons. However, in molecular assemblies, this process commonly yields a strongly exchange-coupled correlated triplet pair, ^1^(TT), where intertriplet interactions facilitate geminate recombination and back-transfer to the singlet manifold. Here we implement an energy-gradient strategy in linear diphenylhexatriene (DPH) hetero-oligomers to guide postfission dynamics toward triplet-pair separation while minimizing thermodynamic loss. The hetero-oligomer series is constructed from two DPH-based building blocks with matched chromophore frameworks but offset triplet energies: a TIPS-substituted unit (T-DPH) and an alkyl-substituted unit (P-DPH). By positioning T-DPH units at the termini of the hetero-oligomers, we encode a modest downhill triplet-energy gradient (∼60 meV) that drives the migration of the triplet population away from the initially formed ^1^(TT) configuration toward the chain periphery. Using femtosecond and nanosecond transient absorption spectroscopy, we track the kinetic evolution from the singlet manifold through ^1^(TT) formation to a spatially separated, weakly exchange-coupled (T··T) state, and ultimately to free triplets. Magnetic-field-dependent transient absorption and photoluminescence measurements provide supporting evidence for population of a weak-exchange triplet-pair regime. Comparison with a homotrimer (PPP-DPH_3_) lacking energetic asymmetry directly isolates the role of this design lever, demonstrating that a ∼60 meV offset is sufficient to accelerate ^1^(TT) → (T··T) separation. Thus, this energy-gradient strategy enhances harvestable free-triplet yield while retaining the high triplet energy required for device-relevant SF optoelectronics and provides a simple and transferable design principle for SF materials that favor productive triplet-pair separation over geminate loss.

## Graphical abstract

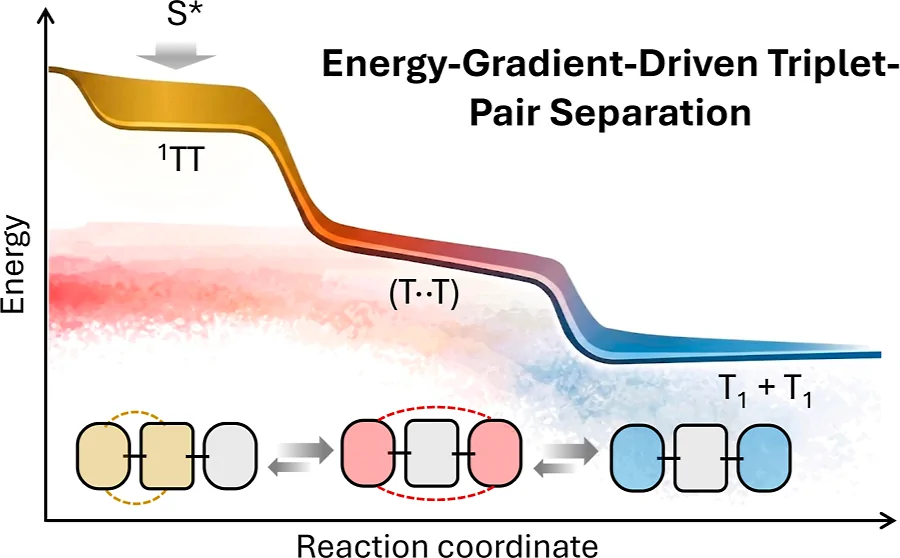

## Introduction

Singlet fission (SF) is a multiexciton generation process that converts one photoexcited high-energy singlet exciton into two lower-energy triplet excitons, offering a pathway to overcome the Shockley–Queisser limit in photovoltaic devices.^1–8^ Realizing this opportunity in practical devices requires molecular architectures that not only enable efficient singlet-to-triplet conversion but also deliver free triplets that can be extracted.^2,9,10^ In molecular assemblies, however, SF typically yields a correlated triplet pair,^1^(TT) whose strong exchange interactions favor geminate recombination and back transfer to the singlet manifold, thereby regenerating emissive singlet character.^1,11–16^ Achieving high yields of long-lived triplets therefore requires the kinetic conversion of this bound pair into weakly exchange-coupled spatially separated (T··T) configurations that can undergo spin evolution and dissociate into free triplets.^15,17,18^

A variety of strategies have been explored to mitigate back-transfer or geminate recombination, including modulating interchromophore coupling via structural bridges, extending from dimers to longer oligomers (and, in some cases, polymers), and controlling solid-state packing and disorder.^16,19–27^ Although these approaches can suppress geminate recombination under specific circumstances, they often fail to enhance the triplet-pair separation. While employing bridges or spacers can prolong ^1^(TT) lifetimes and allow partial dissociation into free triplets by weakening electronic coupling, this often comes at the expense of the initial SF rate or leads to increased radiative loss from the singlet manifold.^20,21,25,28^

Sanders, Campos, Sfeir, and co-workers pioneered an “energy-cleft” design in which intramolecular triplet-energy asymmetry drives a strongly exchange-coupled ^1^(TT) state toward a spatially separated, weakly exchange-coupled multiexciton state, yielding long-lived free triplets.^25,29^ This work established energetic asymmetry within a multichromophoric framework as an effective means of directing triplet-pair fate. However, the large triplet-energy offsets used in such architectures can impose a thermodynamic penalty, because population funnelled to the lowest-energy triplet site carries less extractable energy per exciton.^30^ This trade-off is evident in tetracene–pentacene systems, where the triplet energies differ by ∼0.4 eV, promoting rapid downhill migration but substantially lowering the final triplet energy (*T*_1(tetracene)_ ≈ 1.2 eV vs *T*_1(pentacene)_ ≈ 0.8 eV).^1,20,29,31^

These considerations raise a central design question: how small can the triplet-energy offset Δ*E*_T_ be while still providing a reliable thermodynamic bias for ^1^(TT) → (T··T) separation?

Here, we address this question using an “energy-gradient” motif based on a modest, stepwise triplet-energy bias of ∼60 meV, designed to favor ^1^(TT) → (T··T) separation while preserving a higher average triplet energy across the oligomer. The system under study comprises linear diphenylhexatriene (**DPH**) hetero-oligomers composed of terminal TIPS-substituted units (**T-DPH**) and higher-triplet-energy **P-DPH** units. Using steady-state, ultrafast, and magnetic-field-dependent spectroscopy, we examine how this energetic asymmetry reshapes triplet-pair evolution following singlet fission. Comparison with a homotrimer reference further isolates the role of energetic asymmetry from oligomer length.

## Results

### Molecular Design and Structural Nomenclature

The molecular series investigated in this work is based on the singlet-fission-active diphenylhexatriene (**DPH**) chromophore, assembled into linear hetero-oligomeric chains of increasing length.^32–36^ The series is constructed from two fundamental building blocks, both sharing the same π-conjugated **DPH** core but differing in substitution. **T-DPH** denotes a **DPH** unit bearing a bulky triisopropylsilyl (TIPS) group at the terminal position. Whereas **P-DPH** denote any **DPH** unit lacking terminal **TIPS** substitution, including terminal alkyl-substituted units and units bearing alkoxy substituents at interior positions to ensure sufficient solubility (Figure 1d). Thus, the labels “**P**” and “**T**” distinguish the identity of the **DPH** unit within the hetero-oligomer sequence: “**T**” refers specifically to a terminal **TIPS**-substituted unit, while “**P**” encompasses all remaining **DPH** units. Structural variation within the series arises from two independently controlled parameters: (i) the number of **DPH** units in the hetero-oligomeric chain (oligomer length), and (ii) the arrangement of **P** and **T** units along the chain. This modular architecture preserves the integrity of the **DPH** chromophore framework while enabling systematic fine-tuning of site energies and interchromophore interactions (Figure 1d).

**Figure 1:**
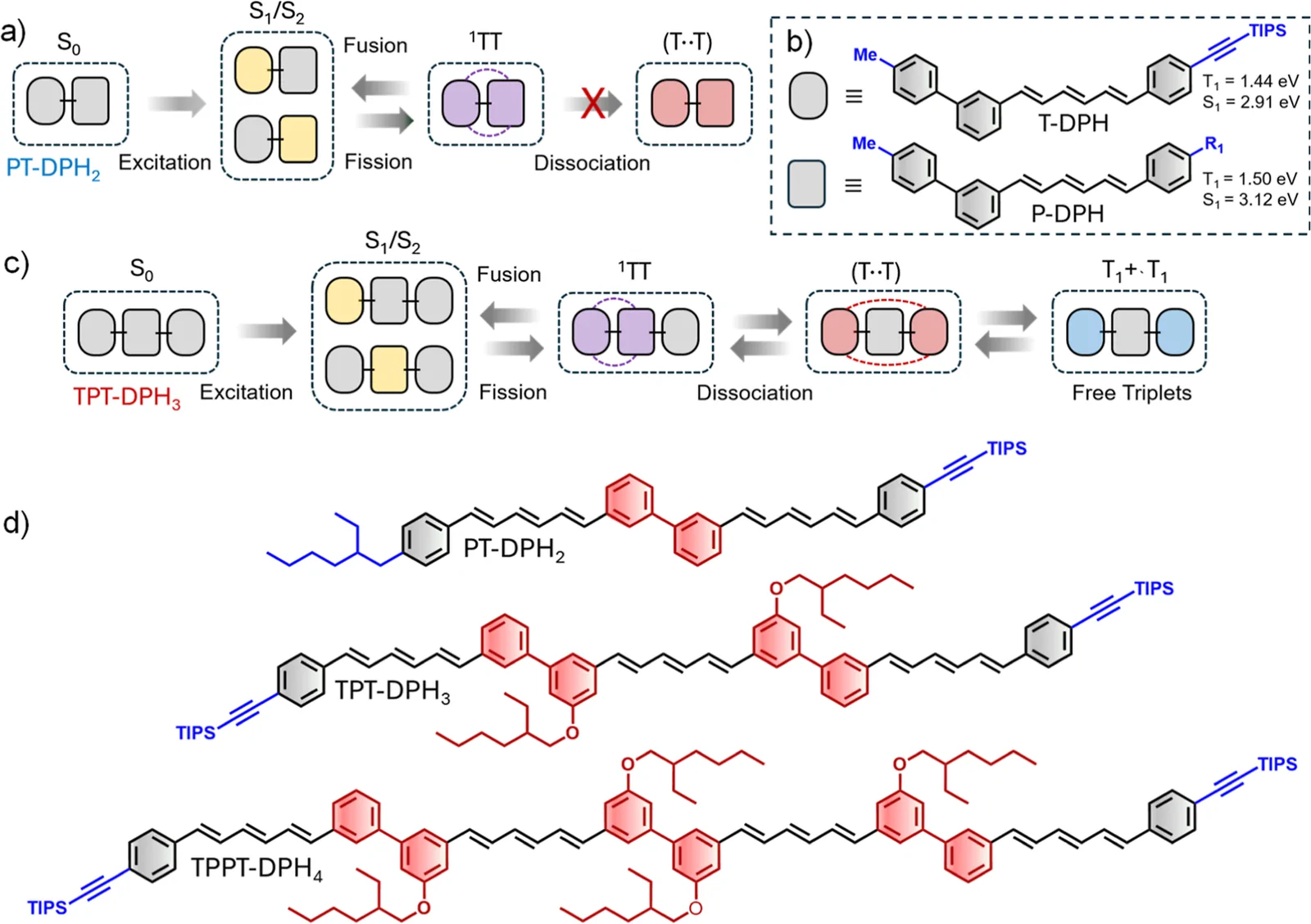
Intramolecular singlet fission (iSF) pathways and molecular structures of DPH-based oligomers. (a) Scheme of iSF in the PT-DPH_2_ dimer, where photoexcitation generates S_1_ and populates the correlated triplet pair ^1^(TT). (b) Monomer reference structures of P-DPH and T-DPH, together with their TD-DFT-calculated singlet and triplet energies. Here, T denotes a TIPS-substituted DPH unit, whereas P denotes an alkyl-substituted DPH unit. (c) Scheme of iSF in TPT-DPH_3_, where formation of ^1^(TT) is followed by triplet migration to a terminal unit to yield a spatially separated correlated (T··T) state. (d) Molecular structures of the oligomeric architectures PT-DPH_2_, TPT-DPH_3_, and TPPT-DPH_4_ (TIPS = triisopropylsilyl).

Crucially, **TIPS** substitution lowers the local triplet energy relative to the **P-DPH** analogue (Figure 1b).^28,37–39^ Accordingly, terminal **T-DPH** units introduce the intended downhill energetic bias within the oligomer.^25^ The nomenclature of each oligomer directly reflects its substitution pattern along the chain. For example, **PT-DPH**_**2**_ is a heterodimer comprising one **P-DPH** and one **T-DPH** unit. **TPT-DPH**_**3**_ is a heterotrimer in which a central **P-DPH** unit, bearing alkoxy substituents to ensure sufficient solubility, is flanked by two terminal **T-DPH** units. Extending this logic, **TPPT-DPH**_**4**_ denotes a heterotetramer bearing **T-DPH** units at both terminal positions and two interior **P-DPH** units functionalized with alkoxy groups. The molecular structures of all oligomers are shown in Figure 1d. These compounds were synthesized through modular cross-coupling sequences from the corresponding DPH building blocks, with full synthetic procedures and characterization provided in the Supporting Information (Schemes S1–S3, Section 4, Supporting Information). The final key coupling steps afforded **PT-DPH**_**2**_, **TPT-DPH**_**3**_, and **TPPT-DPH**_**4**_ in isolated yields of 27%, 19%, and 21%, respectively.

### Optical Properties of Energy-Gradient Oligomers

Normalized steady-state absorption and photoluminescence (PL) spectra of the reference monomers (**T-DPH** and **P-DPH**) and hetero-oligomers (**PT-DPH**_**2**_, **TPT-DPH**_**3**_, and **TPPT-DPH**_**4**_ **)** were recorded in toluene (∼15 μM) at room temperature (Figures 2 and S1–S3). Across the series, the absorption profiles retain the characteristic vibronic structure of the **DPH** chromophore, confirming that the fundamental framework is preserved upon oligomerization. **TIPS** substitution induces a noticeable redshift in the absorption onset relative to **P-DPH**, consistent with DFT-calculated S_1_ energies of ≈3.12 eV for **P-DPH** and ≈2.91 eV for **T-DPH** (Table S1 and Figure S4). Together, these results confirm that **TIPS** substitution selectively lowers the excited-state energy of the terminal units. As a result, the hetero-oligomer spectra appear as a superposition of two monomer-like components: a lower-energy band arising from the **T-DPH** termini and a higher-energy shoulder associated with the interior **P-DPH** units. The relative intensities of these features scale with the stoichiometric **T**-to-**P** ratio, while the absorption maxima shift only minimally upon oligomerization, consistent with weak interunit electronic coupling.^28,40–42^ For the longest hetero-oligomer, **TPPT-DPH**_**4**_, a slight reduction in vibronic contrast is observed, likely reflecting increased conformational heterogeneity or modest exciton delocalization across the extended π-system (Figure S3).

**Figure 2:**
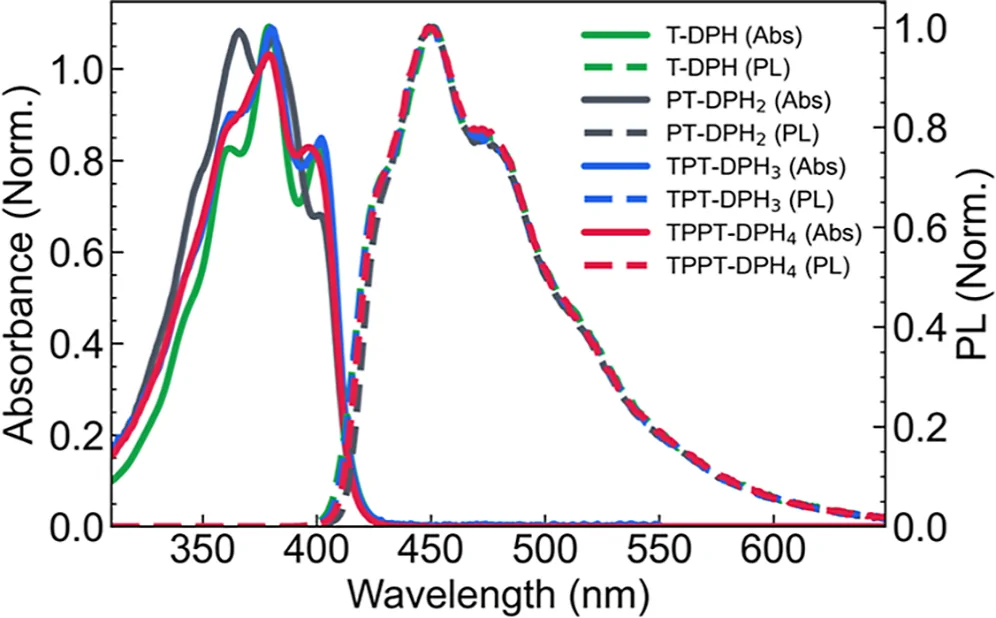
Normalized UV–vis absorption (solid lines) and photoluminescence (PL; dashed lines) spectra of **T-DPH**, **PT-DPH**_**2**_, **TPT-DPH**_**3**_, and **TPPT-DPH**_**4**_ recorded in toluene at room temperature (∼15 μM). PL spectra were acquired with excitation at 370 nm.

In contrast, the PL spectra of all hetero-oligomers are dominated by emission from the lowest-energy **T-DPH**-like state (Figures 2 and S1–S3). The emission maxima and spectral envelopes of all compounds closely overlap, with no pronounced redshift or spectral broadening observed with increasing oligomer length (Table S2). The absence of new low-energy or broadened emission features argues against substantial contributions from excimer-like, charge-transfer-like, or other relaxed emissive states, and instead suggests that the PL remains localized at the **T-DPH** unit.^28,43,44^ These observations are consistent with rapid intramolecular energy transfer from the higher-energy **P-DPH** units to the terminal **T-DPH** units upon photoexcitation.

### Hetero-Oligomer Length Control of Triplet-Pair Evolution Following Singlet Fission

The iSF dynamics of the hetero-oligomers (**PT-DPH**_**2**_, **TPT-DPH**_**3**_, and **TPPT-DPH**_**4**_) were characterized by transient absorption (TA) spectroscopy (Figures 3 and S5–S6). At early delay times following 355 nm photoexcitation, all three hetero-oligomers display a structured transient response in the visible region (420–540 nm) characteristic of the **DPH** singlet excited-state manifold.^16,34^ Following convention in the DPH photophysics literature, we denote this manifold as S* to reflect rapid subpicosecond equilibration among closely spaced singlet states.^16,34^,,^35,45^ At later time delays, **PT-DPH**_**2**_, **TPT-DPH**_**3**_, and **TPPT-DPH**_**4**_ exhibit pronounced spectral evolution marked by the growth of a strong photoinduced absorption (PIA) (≈430–480 nm) accompanied by a partial decay of the initial S* contribution. The emerging PIA band is assigned to the correlated triplet-pair state ^1^(TT), which is formed by singlet fission on the tens-of-picoseconds time scale across all three hetero-oligomer systems. Notably, the singlet signal does not vanish completely, suggesting quasi-equilibrium coupling between S* and ^1^(TT).^16^ In contrast, the **T-DPH** monomer shows predominantly relaxation of the initial singlet manifold, with no clear emergence of a new long-lived feature in the ≈430–480 nm region over the subns window (Figure S7). Kinetic traces for the singlet and triplet-pair channels were extracted by monitoring Δ*T*/*T* at 505 and 460 nm, respectively (Figures 3c, S5c and S6c), where the spectra are dominated by the S* and ^1^(TT) contributions and spectral overlap is minimized.

**Figure 3:**
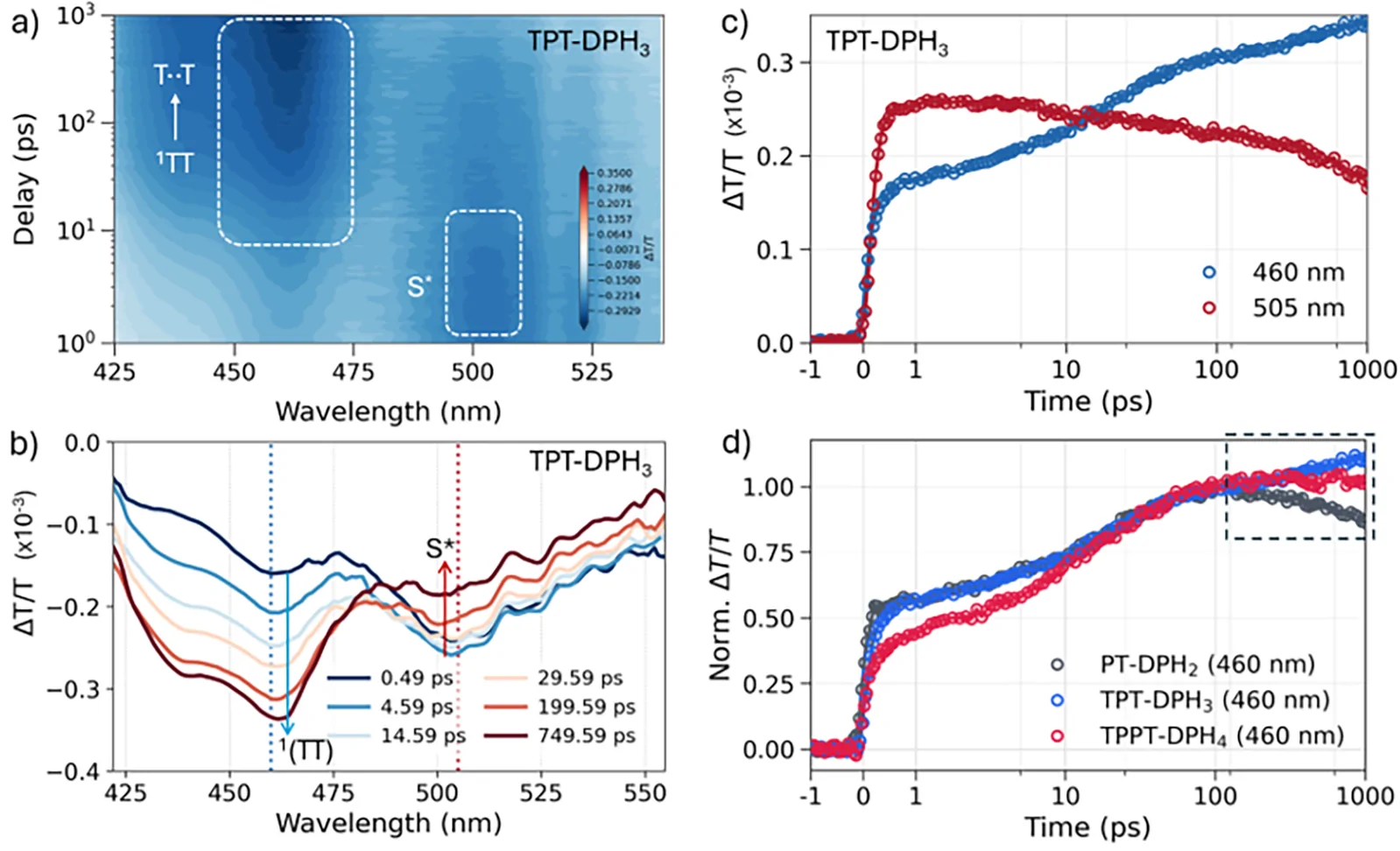
Two-step triplet-pair evolution in TPT-DPH_3_ revealed by femtosecond transient absorption spectroscopy, and its dependence on oligomer length. (a) fsTA map of TPT-DPH_3_ in toluene following 355 nm excitation, showing the early time singlet manifold (S*) and the growth of the correlated triplet-pair band (^1^(TT)), followed by the emergence of a weakly exchange-coupled triplet-pair contribution (T··T). (b) Selected fsTA spectra of TPT-DPH_3_ at representative pump–probe delays, illustrating the redistribution of spectral weight from the singlet region into the triplet-pair region. (c) Kinetic traces extracted for TPT-DPH_3_ from the S* and triplet-pair spectral windows. (d) Comparison of normalized triplet-pair kinetics across the PT-DPH_2_, TPT-DPH_3_, and TPPT-DPH_4_, showing a delayed rise component in the longer oligomers, that is absent in the dimer.

While the initial S* → ^1^(TT) conversion occurs on comparable time scales for all three hetero-**DPH** architectures (≈20–23 ps, Table 1), the subsequent evolution of the triplet-pair manifold depends systematically on oligomer length. In the dimer (**PT-DPH**_**2**_), the kinetics are well described by a reversible coupling between S* and ^1^(TT), establishing a quasi-equilibrium in which residual singlet character persists alongside the triplet-pair population (Figure S5). The ^1^(TT) state in **PT-DPH**_**2**_ primarily undergoes geminate recombination, with no clear secondary evolution into a more spatially separated triplet-pair configuration, indicating that the two-chromophore architecture does not provide sufficient separation to weaken exchange coupling and promote triplet-pair dissociation.^16,47^

**Table 1:** Photoluminescence Lifetimes and Quantum Yields, Singlet-Fission Time Constants, and Correlated Triplet-Pair and Free-Triplet Yields of Hetero-DPH Oligomers in Toluene

material	τ_PL_ (ns)	*k*_SF_(10^–3^ ps^–1^)	*k*_–SF_(10^–3^ ps^–1^)	*k*_TT_(10^–4^ ps^–1^)	*k*_T··T_(10^–4^ ps^–1^)	ϕ_PL_ (%)	ϕ_TT_ (%)	ϕ_T1_ (%)
PT-DPH_2_	4.85 (92.2%)	43.78	7.31	1.83	-	82	86	10
	9.08 (7.8%)							
TPT-DPH_3_	1.93 (36.2%)	50.63	4.89	3.59	1.00	53	91	89
	3.07 (62.3%)							
	9.99 (1.5%)							
TPPT-DPH_4_	2.76 (78.0%)	47.71	9.39	2.79	0.59	54	84	73
	4.18 (21.6%)							
	17.9 (0.4%)							

In contrast, the trimer and tetramer (**TPT-DPH**_**3**_ and **TPPT-DPH**_**4**_) exhibit an additional, delayed growth component in the triplet-pair population dynamics that emerges on a longer time scale following initial S* → ^1^(TT) conversion (Figures 3 and S6). This slower component is assigned to evolution from the initially formed, strongly exchange-coupled ^1^(TT) state into a more weakly exchange-coupled, spatially separated triplet-pair manifold, denoted (T··T) (Figures 1b and S8). We use (T··T) as a shorthand for this separated weak-exchange manifold, rather than as a strictly spin-pure singlet eigenstate, because spin mixing becomes important as the intertriplet exchange interaction decreases.^16,48^ Back transfer from ^1^(TT) to the singlet manifold remains operative across the series, however, in the longer oligomers (**TPT-DPH**_**3**_ and **TPPT-DPH**_**4**_) the pathway provides a kinetically competing channel (^1^(TT) → (T··T)) that draws population away from the singlet manifold, suppressing radiative recombination. The fsTA data are well reproduced by a minimal target model in which S* and ^1^(TT) are in quasi-equilibrium in **PT-DPH**_**2**_, whereas in **TPT-DPH**_**3**_ and **TPPT-DPH**_**4**_ this coupled S*/^1^(TT) manifold undergoes subsequent conversion to (T··T) (Figures S9–S11, Section 1.7, Supporting Information). The singlet fission yield, Φ_TT_, was estimated from an equilibrium model of S* and ^1^(TT) and is high across the series: 86% for **PT-DPH**_**2**_, 91% for TPT-DPH3, and 84% for **TPPT-DPH**_**4**_ (Table 1).^16^

The delayed rise in triplet-pair dynamics was further investigated using nanosecond transient absorption (nsTA) spectroscopy. For **PT-DPH**_**2**_, the nsTA response is dominated by the ^1^(TT) photoinduced absorption, which decays over the measurement window with a lifetime of τ_1_ = 5.45 ns, leaving behind a small residual signal of free triplet attributed to isolated triplet state population via S* → T_1_ intersystem crossing (ISC, $\tau_{\left(T_{1}\right)}$ = 33.53 μs, Figures 4 and S13).^16^ The extracted kinetics (Figure 4c, inset) are consistent with a picture in which the correlated triplet pair primarily undergoes geminate recombination, either to the ground state or via back-transfer to the singlet manifold, rather than progressing to fully dissociated triplets.

**Figure 4:**
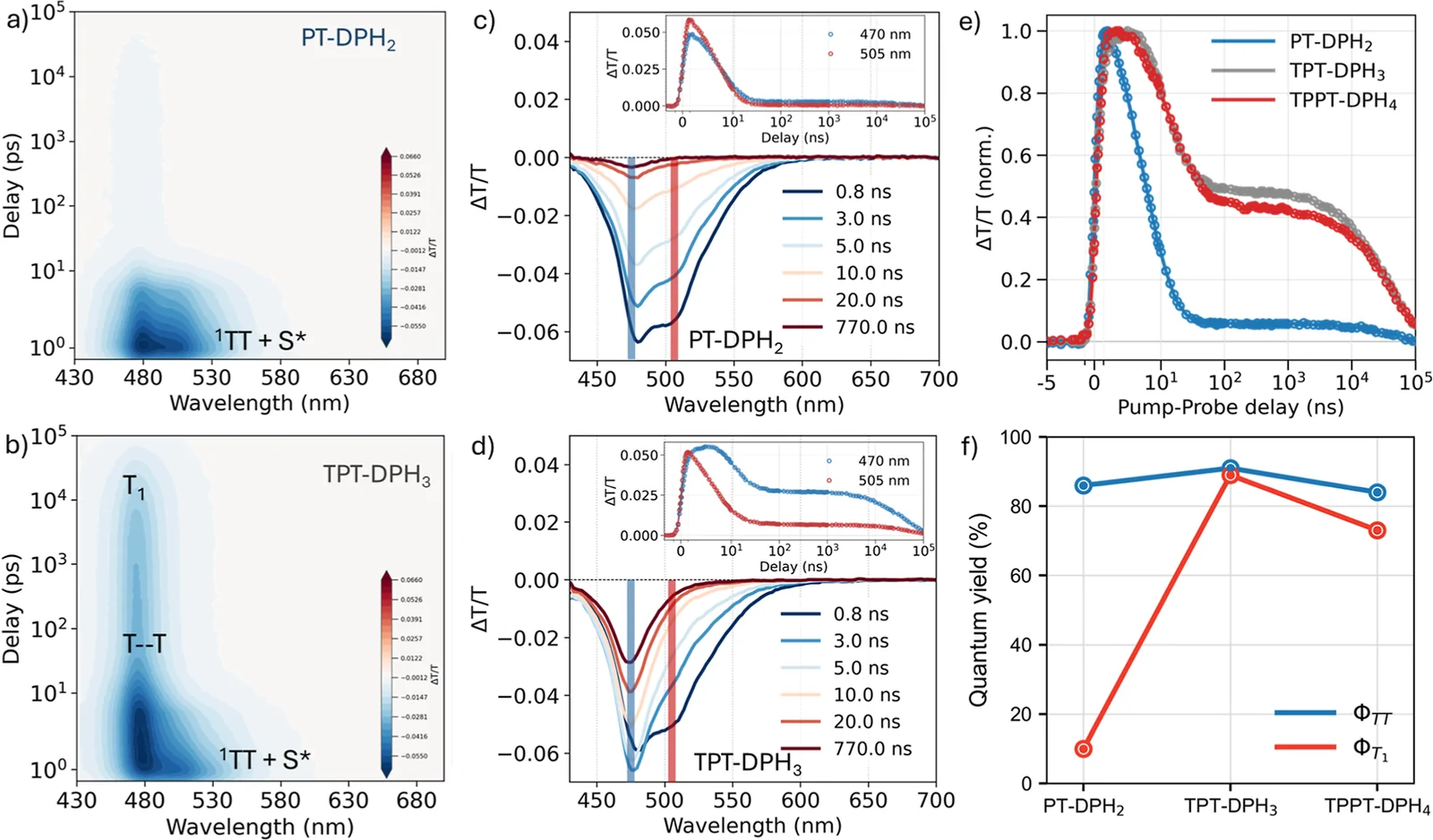
Nanosecond transient absorption reveals length-dependent triplet-pair separation and free-triplet formation in DPH oligomers. (a,b) nsTA maps of PT-DPH_2_ (dimer) and TPT-DPH_3_ (trimer) in toluene following 355 nm excitation. (c,d) Selected nsTA spectra at representative delays for the dimer and trimer, respectively; insets show kinetic traces at 470 and 505 nm used to track triplet-pair and triplet-related signals. (e) Normalized long-time populations comparing the dimer, trimer, and tetramer. (f) Length-dependent evolution of triplet-pair and free triplet formation yields in the DPH oligomer series.

The longer hetero-oligomers show a distinct evolution from ^1^(TT) to (T··T), as evidenced by the initial rise in the nsTA triplet-pair population (Figures 4, S14 and S15). In **TPT-DPH**_**3**_, the nsTA map and spectra reveal the growth of a new spectral contribution that closely resembles the sensitized triplet PIA of **T-DPH**, accompanied by clear spectral reshaping on the subnanosecond to few-nanosecond time scale (Figures 4b,d and S13). This evolving feature is assigned to the formation of weakly exchange-coupled, spatially separated triplet-pair state, (T··T). Subsequently, the (T··T) state feeds a long-lived free-triplet (T_1_) population on the microsecond time scale (Figures 4 and S12), consistent with spatial separation facilitating productive triplet-pair dissociation. Global analysis of the nsTA kinetics resolves three sequential components representing the ^1^(TT), (T··T), and T_1_ states, respectively. The associated lifetimes were determined to be τ_1_ = 2.69 ns for ^1^(TT), τ_2_ = 9.79 ns for (T··T), ultimately yielding a long-lived free triplet population with $\tau_{\left(T_{1}\right)}$ = 30.97 μs (Figure S14).

The heterotetramer, **TPPT-DPH**_**4**_, shows analogous nsTA behavior, characterized by a slow evolution from ^1^(TT) to (T··T) and the subsequent growth of a long-lived T_1_ population (Figures 4e and S15). The corresponding lifetimes are τ_1_ = 3.57 ns for ^1^(TT), τ_2_ = 16.72 ns for (T··T) and $\tau_{\left(T_{1}\right)}$ = 46.70 μs for T_1_. The slower ^1^(TT) → (T··T) conversion in **TPPT-DPH**_**4**_ relative to **TPT-DPH**_**3**_ likely arises from the broader distribution of initially formed ^1^(TT) configurations accessible in the longer chain. Specifically, triplet pairs formed on the central **P**–**P** chromophore pair can evolve directly downhill toward the terminally separated (T··T) state. Triplet pairs formed on the terminal **T–P** chromophore pair, however, require longer-range redistribution before reaching the fully separated configuration (Figure S8). The measured ^1^(TT) → (T··T) conversion rate in **TPPT-DPH**_**4**_ therefore reflects an ensemble-averaged redistribution bottleneck rather than a single elementary rate-limiting step.

The free-triplet quantum yield, evaluated by triplet-plateau analysis (Supporting Information Section 1.8), increases dramatically from 10% in **PT-DPH**_**2**_ to 89% in **TPT-DPH**_**3**_, with a modest reduction to 73% in **TPPT-DPH**_**4**_ (Figure 4f and Table 1). These results demonstrate that extending beyond the dimer architecture strongly improves triplet separation, but that the benefit is not monotonic with oligomer length. The reduced free triplet yield of **TPPT-DPH**_**4**_ relative to **TPT-DPH**_3_ is consistent with the broader distribution of triplet-pair configurations and redistribution pathways available in the longer oligomer, as discussed above and illustrated in Figure S8. These additional pathways can increase the probability of geminate recombination or nonemissive decay before productive free-triplet formation. Within this series, **TPT-DPH**_**3**_ provides the most favorable balance between triplet-pair formation and efficient downhill migration to (T··T) configuration, making it the most effective architecture for free-triplet generation in this **DPH** hetero-oligomer family. These trends indicate that chain extension beyond the dimer introduces a downhill coordinate that stabilizes separated triplet-pair character and reduces radiative singlet recovery. Consistent with this picture, the PLQY drops substantially upon introduction of the energetic gradient, from 82% in **PT-DPH**_**2**_ to 53% and 54% in **TPT-DPH**_**3**_ and **TPPT-DPH**_**4**_, respectively.

Importantly, the remaining substantial PLQY in **TPT-DPH**_**3**_ and **TPPT-DPH**_**4**_ does not indicate inefficient singlet fission. The transient absorption data show efficient formation of ^1^(TT) in both hetero-oligomers. Rather, the residual emission reflects partial reversibility of the coupled S*/^1^(TT) manifold. Population that enters the triplet-pair manifold can return to emissive singlet character through back-transfer or triplet-pair recombination, giving rise to regenerated or delayed fluorescence. Although the steady-state PLQY cannot be quantitatively decomposed into prompt and delayed components without absolute time-gated PLQY measurements, the multiexponential time-resolved PL decays support this mixed prompt/regenerated-emission picture. Thus, high ^1^(TT) and free-triplet yields can coexist with substantial PLQY when singlet fission is partially reversible.

### Magnetic Field Effect on iSF of Energy-Gradient Hetero-Oligomers

To probe whether the delayed-rise species is consistent with formation of a weakly exchange-coupled separated triplet pair, we measured magnetic-field-dependent nanosecond transient absorption (nsTA) and photoluminescence (PL) in the energy-gradient hetero-oligomers (Figure 5). Low-field magnetic-field effects are expected to be negligible for strongly exchange-coupled ^1^(TT) pairs, for which related DPH systems show a triplet exchange interaction of 1–2 meV,^49^ but they are expected to become pronounced when the triplet pair evolves into a more weakly exchange-coupled (T··T) configuration.^16,27,32,48,50^

**Figure 5:**
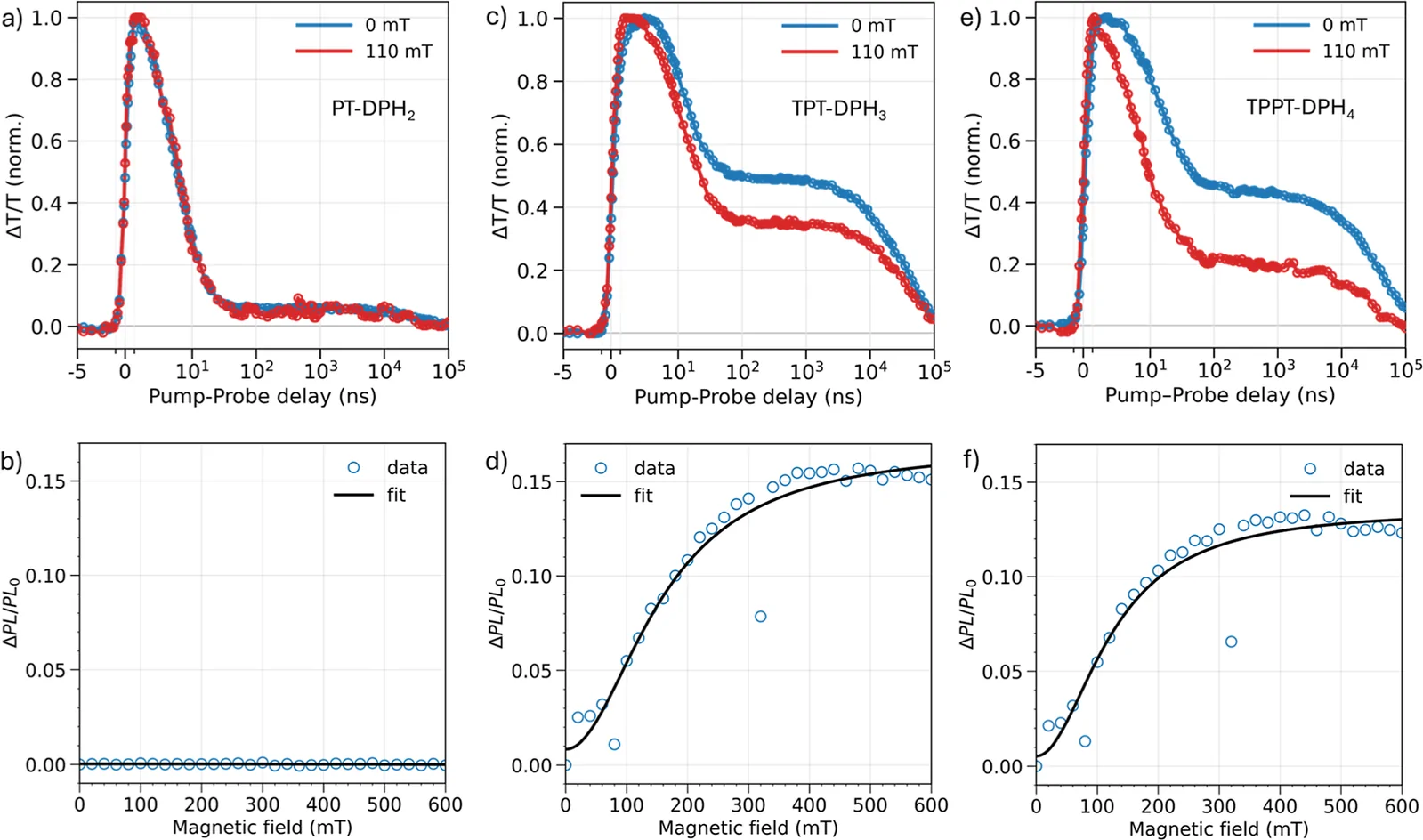
Magnetic-field dependence of iSF dynamics on energy-gradient hetero-oligomers. (a,c,e) Normalized nanosecond transient absorption kinetics, Δ*T*/*T*, measured in the triplet-pair region for **PT-DPH**_**2**_ (dimer), **TPT-DPH**_**3**_ (trimer), and **TPPT-DPH**_**4**_ (tetramer) at 0 mT (blue) and 110 mT (red). (b,d,f) Magnetic field dependence of the relative photoluminescence change, ΔPL/PL_0_, for PT-DPH_2_ (dimer), TPT-DPH_3_ (trimer), and TPPT-DPH_4_ (tetramer). Solid lines are fitted to a standard empirical saturation model.

In the dimer (**PT-DPH**_**2**_), the nsTA kinetics and the free triplet amplitude remain essentially unchanged upon application of a magnetic field (Figures 5 and S16). The PL intensity is likewise field independent over the full field range examined, indicating that any (T··T) contribution in the dimer is minimal under these conditions. This behavior is consistent with triplet-pair dynamics dominated by strongly exchange-coupled, ^1^(TT) states that either undergo geminate recombination or return reversibly to the singlet manifold without accessing a weakly exchange-coupled regime. This interpretation is further supported by the high photoluminescence quantum yield of ϕ_PL_ = 82% measured for **PT-DPH**_**2**_.

In contrast, both the trimer and the tetramer exhibit clear magnetic-field effects in nsTA and PL (Figures 5 and S17, S18). An applied field of 110 mT modifies the long-time TA amplitude and kinetics, consistent with magnetic-field-sensitive spin evolution within the weakly exchange-coupled triplet-pair manifold. Specifically, the applied field lifts the degeneracy of the triplet-pair spin sublevels through the Zeeman effect, reducing the efficiency of spin-mixing-driven triplet separation.^51,52^ Consequently, the (T··T) population undergoes faster geminate recombination, and the amplitude of free-triplet formation is reduced.

Accordingly, the free-triplet yield decreases from 89% to 64% in the trimer and from 73% to 37% in the tetramer. In parallel, the PL shows a pronounced magneto-PL response, with ϕ_PL_ increasing monotonically as the field strength increases (vide infra). Together, these results indicate that a significant fraction of the excited-state population in the trimer and tetramer passes through the weakly exchange-coupled (T··T) manifold, whose spin evolution is perturbed by the applied field, enhancing singlet return and radiative recombination.

The magnetic-field dependence of PL intensity is well described by a Lorentzian-like saturation profile (Figure 5 and Section S1.9).^36,53^ The trimer, **TPT-DPH**_**3**_, exhibits a pronounced magneto-PL response that saturates at approximately 16% with a characteristic field scale of approximately 159 mT. **TPPT-DPH**_**4**_ shows a similar but slightly smaller response, saturating at approximately 13%, with a field scale of approximately 125 mT. The fact that both the nsTA and PL signals change on the same low-field scale (∼0.1 to 0.2 T) indicates a common underlying spin-dependent mechanism.

At these field strengths, the Zeeman energy is only on the order of 0.01 meV, so the observation of sizable MFEs at such low fields is consistent with weak intertriplet exchange coupling, as expected for a spatially separated (T··T) state rather than a strongly exchange-coupled ^1^(TT) pair.^54^ Although delayed-fluorescence quantum beating has been observed in crystalline singlet-fission materials such as tetracene,^55,56^ no clear beating is resolved in the present room-temperature solution measurements (Figures S5 and S6). Thus, the assignment of the separated weak-exchange (T··T) manifold is based on the combined fsTA/nsTA, PL, and magnetic-field evidence. The absence of a negative low-field magneto-PL signature indicates that the measured PL does not reflect a pure Merrifield-type response of triplet pair.^16,35,48^ Rather, it reflects field-dependent triplet-pair recombination superimposed on background PL and recombination from a more weakly exchange-coupled or spatially separated (T··T) state.^16,51,53,57^ In addition, rapid rotational diffusion and orientational averaging in toluene at room temperature may further attenuate the low-field negative feature.^27,54,58^

Interestingly, the magnetic-field-induced suppression of free-triplet yield is more pronounced in the tetramer than in the trimer, whereas the corresponding PL enhancement is similar in both oligomers. This mismatch indicates that the field-suppressed population in **TPPT-DPH**_**4**_ is not redirected exclusively into emissive singlet return but is instead lost in part through nonemissive decay pathways. This result is consistent with the tetramer accessing a more weakly coupled separated triplet-pair manifold, in which the applied field more strongly shifts the balance away from free-triplet formation and toward nonemissive decay. Thus, increasing oligomer length changes not only the extent of triplet-pair separation, but also the fate of the separated pair.

### Energy-Gradient Control of Triplet-Pair Fate

To isolate the effect of energetic asymmetry from oligomer length, we compare the energy-gradient heterotrimer **TPT-DPH**_**3**_ with the previously reported homotrimer **L-(mDPH)**_**3**_, which we denote here as **PPP-DPH**_**3**_ to align with the P/T sequence nomenclature used throughout this work (Figure 6b).^16^ Because both systems share the same chain length and chromophore framework, differences in postfission dynamics can be attributed primarily to the altered triplet-energy landscape introduced by terminal **T-DPH** substitution. The initial SF rate is broadly similar in the two trimers; the ^1^(TT) formation time constant is ≈ 19.75 ps for **TPT-DPH**_**3**_ and 18.00 ps for reference **PPP-DPH**_**3**_. This similarity indicates that terminal substitution perturbs the initial interchromophore electronic coupling only modestly.

**Figure 6:**
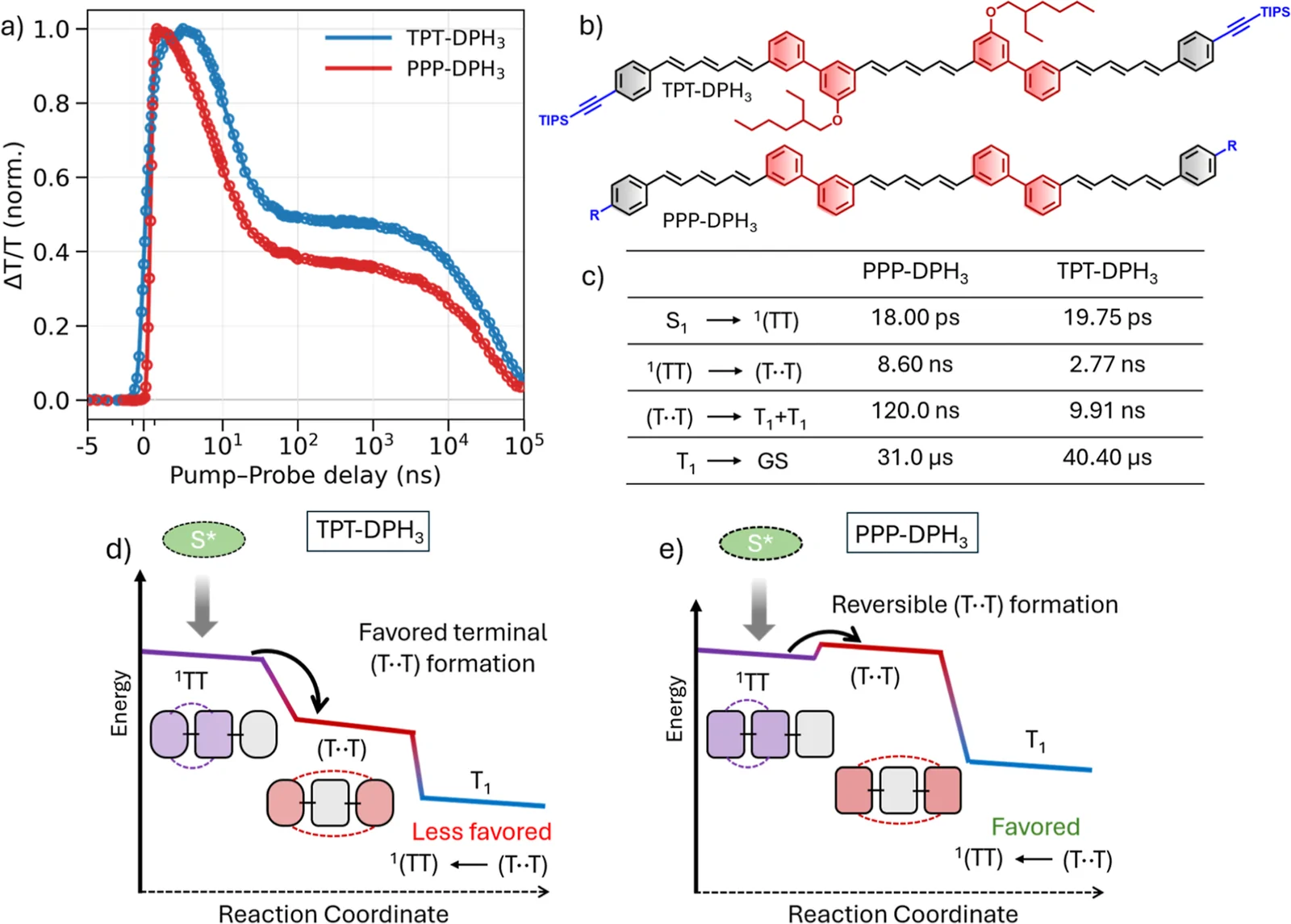
**Comparison of energy-gradient trimer against a homotrimer reference**. (a) Normalized long-time triplet-derived population traces (Δ*T*/*T*) comparing the reference PPP-DPH_3_ trimer and the energy-gradient TPT-DPH_3_ trimer (b) molecular structures of the energy gradient heterotrimer (TPT-DPH_3_) and the homotrimer (PPP-DPH_3_), respectively, (c) apparent kinetic parameters extracted from the simplified kinetic analysis; these values are used to compare the two trimers rather than to assign unique microscopic elementary steps. (d,e) Schematic energy-landscape comparison for triplet-pair evolution in PPP-DPH_3_ and TPT-DPH_3_.

The key difference instead lies in the energetics of the resulting triplet-pair states. In **TPT-DPH**_**3**_, the initially formed bound ^1^(TT) state is associated with an adjacent **T–P** chromophore pair. Using the monomer triplet energies (Figure 1b) and an illustrative intertriplet exchange coupling *J*, this heteropair ^1^(TT) state of **TPT-DPH**_**3**_ is expected to be stabilized below the separated (T··T) reference by approximately 2*J* (Figure S19). However, a separated (T··T) configuration with the two-triplet localized on the two terminal **T-DPH** sites lies lower in energy because it places population on the lower-energy **T-DPH** sites, while largely suppressing exchange stabilization (Figure 6d).

By contrast, in the homotrimer **PPP-DPH**_**3**_, spatially separated triplet-pair configurations are still accessible, as shown previously.^16^ However, the initially formed bound ^1^(TT) state resides on an adjacent **P–P** pair, and because all three chromophores are energetically equivalent, formation of the separated (T··T) configuration gains no compensating site-energy advantage. As a result, separation from ^1^(TT) to (T··T) is expected to be mildly uphill by roughly 2*J* (a few meV, Figures 6e and S19).^48,59^ This does not prevent triplet-pair separation in **PPP-DPH**_**3**_, but it makes the separated-pair manifold more reversible, allowing population to return more readily to adjacent ^1^(TT)-like configurations where back-transfer to the singlet manifold or geminate recombination remains competitive. Thus, relative to **PPP-DPH**_3_, the energy-gradient trimer **TPT-DPH**_**3**_ possesses a clear thermodynamic driving force that favors net population of the separated triplet-pair state. Although the effective J for a heteropair may differ somewhat from that of the homopair in **PPP-DPH**_**3**_, this would alter the exchange stabilization of ^1^(TT) by only a few meV and is therefore negligible relative to the much larger ∼60 meV triplet-energy bias driving downhill relaxation.

As a result, ^1^(TT) population decays substantially faster in the energy-gradient heterotrimer (*k* = 0.36 ns^–1^) than in the homotrimer (*k* = 0.12 ns^–1^, Figure S20). Likewise, the rate of free-triplet formation is faster in **TPT-DPH**_**3**_ than in **PPP-DPH**_**3**_, demonstrating that the (T··T) manifold in the energy-gradient architecture more efficiently feeds long-lived T_1_ population (Figure 6c). Because the triplet-pair and isolated-triplet photoinduced absorption features overlap, the kinetic parameters in Figure 6c should be regarded as apparent rates describing the dominant spectral evolution, rather than unique microscopic rate constants for individual elementary steps. The simplified kinetic scheme therefore captures the net postfission evolution used to compare.

**PPP-DPH**_**3**_ and **TPT-DPH**_**3**_, but does not separately resolve every parallel pathway, including geminate recombination, back-transfer, and formation or decay of isolated T_1_ population. Consistent with these accelerated postfission steps, the free-triplet yield increases from 68% for **PPP-DPH**_**3**_ to 89% for **TPT-DPH**_**3**_, with both values evaluated using the same triplet-plateau analysis (Supporting Information Section 1.8). Together, these results show that introducing a downhill triplet-energy while retaining the same chromophore core redirects a larger fraction of the postfission population away from geminate loss and back transfer and toward productive separated triplet states (Figure 6a,c).

## Conclusion

In this work, we establish an energy-gradient design principle for controlling the fate of correlated triplet pairs in **DPH** hetero-oligomers. Terminal TIPS substitution introduces a modest downhill triplet-energy gradient that leaves initial ^1^(TT) formation largely intact in hetero-oligomers, but it promotes subsequent evolution into a weakly coupled (T··T) state in **TPT-DPH**_**3**_ and **TPPT-DPH**_**4**_, but not in **PT-DPH**_**2**_. This shift in postfission dynamics in the longer hetero-oligomers is accompanied by a large increase in free-triplet yield, reaching 89% in the energy-gradient trimer. Benchmarking against the homotrimer **PPP-DPH**_**3**_ further isolates the role of energetic asymmetry, showing that the enhanced triplet separation arises primarily from the altered triplet-energy landscape rather than from chain length alone. Together, these results demonstrate that a modest triplet-energy offset of ∼60 meV is sufficient to redirect triplet-pair fate away from reversible ^1^(TT) ⇄ (T··T) cycling, geminate recombination, and back-transfer, and toward effective separation and long-lived free-triplet formation, without sacrificing the high triplet energy required for device-relevant applications.

This shallow-gradient strategy also highlights an important design trade-off. A small triplet-energy offset preserves high triplet energy, but does not deeply stabilize the separated triplet-pair state, allowing back-transfer, reverse triplet–triplet annihilation or delayed fluorescence to remain competitive. Future implementations should therefore combine small-offset energy gradients with efficient triplet-harvesting pathways, for example through triplet transfer to acceptors, coupling to semiconductor interfaces, or modest additional energetic bias at the final harvesting site. In this form, energy-gradient engineering offers a simple and transferable design principle for singlet-fission materials that favor productive triplet-pair separation without requiring large energetic penalties or exotic chromophore combinations.

## Supplementary Material



## Notes

It contains materials and methods; computational details; steady-state and time-resolved spectroscopy with global analysis; additional figures and tables; full synthetic procedures and characterization data (PDF)
