# Supplementary material for: An Energy-Gradient Strategy for Triplet-Pair Separation in Singlet-Fission Oligomers
Source: J Am Chem Soc. 2026 Aug 14;148(33):35973–82. doi: 10.1021/jacs.6c09940 (PMC13523692; doi:10.1021/jacs.6c09940)
Supplement: Supplementary file 1 [file ja6c09940_si_001.pdf]

# An Energy-Gradient Strategy for Triplet-Pair Separation in Singlet-Fission Oligomers.

Ebin Sebastian†1, Stephanie Montanaro†2, Oliver Millington[2], Ashish Sharma[1], Anastasia Leventis[2], Julia Osmólska[1], Daniel G. Congrave[3], Akshay Rao*1 and Hugo Bronstein*2

*[1]Cavendish Laboratory, University of Cambridge, J. J. Thomson Avenue, Cambridge, CB3 0HE, UK.*

*[2]Department of Chemistry, University of Cambridge, Lensfield Road, Avenue, Cambridge, CB2 1EW, UK.*

*[3]Chemistry Research Laboratory, University of Oxford, Oxford, OX1 3TA, UK.*

† These authors contributed equally

*Email: hab60@cam.ac.uk E-mail: ar525@cam.ac.uk

## Supporting Information

# Table of Contents

**Section 1: Materials and Methods ........ 2**

1.1 Computational Analysis ........ 2
1.2 Steady-state absorption and photoluminescence ........ 2
1.3 Time-resolved photoluminescence ........ 3
1.4 Photoluminescence quantum efficiency (PLQE) ........ 3
1.5 Femtosecond transient absorption (fsTA) ........ 3
1.6 Nanosecond transient absorption (nsTA) ........ 3
1.7 Global Analysis ........ 4
1.8. Estimation of Yields of Triplet-Pair States and Isolated Triplets. ........ 4
1.9. Magnetic-field-dependent photoluminescence (MagPL) analysis ........ 5

**Section 2: Tables ........ 7**

**Section 3: Figures ........ 8**

**Section 4: Syntheses and Characterisation ........ 20**

**Reference ........ 35**

## Section 1: Materials and Methods

Glassware was routinely dried in an oven set to 200 °C before use. Flash column chromatography purifications were performed on an autocolumn (Biotage® Isolera) using pre-packed silica gel cartridges purchased from Biotage®. Column conditions were obtained by analytical thin layer chromatography (TLC) using precoated glass backed silica gel plates (Merck Kieselgel 60 F254 0.20 mm). Visualisation was achieved throughout using UV light (254, 365 nm). Nuclear magnetic resonance spectroscopy was performed on a Bruker 400 MHz Avance III HD Spectrometer, a Bruker 500 MHz Avance III HD Spectrometer or a Bruker 700 MHz TXO Cryoprobe Spectrometer. All spectra taken on these machines were recorded at ambient temperature in the stated solvent with residual protic solvent used as the internal standard. 1H NMR data is reported as: chemical shift (multiplicity, coupling constant, integration). 13C NMR data is reported as: chemical shift. Chemical shifts (δ) are quoted in parts per million (ppm) relative to residual solvent (CDCl3: δ(1H) = 7.26 ppm, δ(13C) = 77.16 ppm); Standard abbreviations are used to indicate multiplicities and peak forms: s = singlet, d = doublet, t = triplet, q = quartet, p = pentet, sx = sextet, sp = septet, m = multiplet, br = broad and associated combinations thereof. Coupling constants (J) are quoted to the nearest 0.1 Hz and are assumed to be JH-H unless otherwise stated. Mass spectra were obtained using a Waters LCT, Finnigan MAT 900XP or Waters MALDI micro MX spectrometer by the mass spec team at the Department of Chemistry, University of Cambridge.

All commercial chemicals were of ≥95% purity and were used as received without further purification. Anhydrous solvents were purchased from Sigma Aldrich or Acros Organics and used as received for synthesis. Once synthesized, final DPH materials were stored under argon at ~4 °C or in a nitrogen glovebox at ambient temperature. All optical measurements were carried out in 1 mm pathlength quartz cuvettes. Samples were prepared in a nitrogen-atmosphere glovebox using degassed, anhydrous solvents. Cuvettes were sealed prior to measurements with a PTFE cap, PTFE tape, and parafilm, to ensure the absence of oxygen inside the samples.

### 1.1 Computational Analysis

Geometry optimizations of all molecular system under study were carried out using density functional theory (DFT) as implemented in the Gaussian 16 program package[1]. The B3LYP functional combined with the cc-pvdz basis set was employed for all optimizations. Vertical excitation energies and oscillator strengths were calculated employing time-dependent DFT (TD-DFT) at the B3LYP functional and cc-pvdz level of theory.

### 1.2 Steady-state absorption and photoluminescence

Steady-state absorption across the UV/Vis/NIR spectral regions were carried out using a Shimadzu UV3600Plus spectrometer. Steady-state photoluminescence spectra were measured using an Edinburgh Instruments FLS1000-DD-stm spectrometer, comprised of a 450-xenon lamp for excitation and a FLS1000 double excitation and double emission monochromator. The emissions were detected using a PMT-980 detector.

## 1.3 Time-resolved photoluminescence

Time-resolved photoluminescence was carried out on an Edinburgh Instruments FLS1000-DD-stm spectrometer. The time-resolved photoluminescence of the molecules (ns timescale) was measured using time correlated single photon counting (TCSPC). Samples were excited with a pulsed 375 nm EPL-375, diode laser at a 2 MHz repetition rate (~60 ps pulsewidth). Pump scatter from the laser excitation was filtered out using an appropriate Thorlabs long-pass filter in the emission path. The photoluminescence was directed through an FLS1000 double emission monochromator and detected by a PMT-980 detector in TCSPC mode. The instrument response was determined using light scattered off a piece of scratched glass at the excitation wavelength, giving a value of 137 ps.

## 1.4 Photoluminescence quantum efficiency (PLQE)

PLQE measurements were carried out inside a Spectralon coated integrating sphere following the procedure of de Mello et al.[2] Measurements were taken at room temperature using a temperature and current controlled 375 nm Thorlabs continuous wave laser diode as the excitation source. Light from the experiment was collected using an optical fiber connected to an Andor Kymera 328i Spectrometer housing a DU420A Silicon CCD detector. Setup calibration was performed using a Bentham 610 QTH calibration source. PLQE calculations were performed as described by de Mello et al.[2]

## 1.5 Femtosecond transient absorption (fsTA)

Femtosecond transient absorption (TA) measurements were performed on a HARPIA system (Light Conversion). The fundamental output of a PHAROS femtosecond laser (1030 nm, 10 kHz; Light Conversion) was split to provide pump and probe paths. Tunable pump pulses were generated using an ORPHEUS optical parametric amplifier (Light Conversion) operated in a collinear geometry. For the probe, UV–blue, white-light continuum (≤ ~520 nm) was produced by first frequency-doubling the 1030 nm beam to 515 nm in a β-$BaB_2O_4$ (BBO) crystal and then focusing the 515 nm pulses into a sapphire plate. For longer-wavelength coverage (> ~520 nm; visible–NIR), the 1030 nm fundamental was focused directly into sapphire to generate the supercontinuum. Pump and probe were spatially and temporally overlapped at the sample, with the delay controlled by the HARPIA delay line. To suppress rotational-diffusion artifacts, the pump polarization was set to the magic angle (54.7°) relative to the probe. Transient signals were recorded as ΔA (λ, t).

## 1.6 Nanosecond transient absorption (nsTA)

A 532 nm pump beam was generated by the second harmonic, respectively, of a Q-switched Nd:YVO4 (1 ns pump length, Advanced Optical Technologies Ltd AOT-YVO-25QSPX) laser. A LEUKOS Disco 1 UV low timing jitter supercontinuum laser (STM-1-UV) was used to generate the probe beam. The probe beam was split by a 50% beam splitter into a reference and probe, and both were focused and directed through the sample – only the probe beam interacted, i.e. was overlapped, with the pump beam. This allows for correction of any shot-to-shot fluctuations. A pair of line image sensors (Hamamatsu, G11608) mounted on a spectrograph (Andor Solis, Shamrock SR-303i) were used to detect the signal, using a custom-built board from Entwickslungsbüro Stresing to read out the signal. The delay was controlled electronically for all nanosecond transient absorption measurements, and every second pump pulse is omitted. The average fractional differential transmission (ΔT/T) of the probe is calculated after each time delay following the collection of 1000 shots.

## 1.7 Global Analysis

Global analyses of the fsTA and nsTA spectra were performed using the Glotaran software.[3,4] The fitting workflow explicitly accounts for the instrument response function (IRF) and the chirp of the white-light continuum, enabling simultaneous fitting of all probe wavelengths to extract dispersion-corrected spectra and kinetic parameters.

First, the spectra were analysed using a sequential (global) model to obtain evolution-associated difference spectra (EADS/EAS). In this approach, all wavelengths are fitted concurrently with a chain of exponentially decaying components, yielding time constants and the corresponding EADS that describe how the spectrum evolves. Importantly, EADS/EAS are model-dependent spectral components and the associated time constants do not necessarily correspond to distinct physical/chemical species; rather, they provide a compact description of spectral evolution on different timescales.

To obtain a more mechanistic description of the singlet-fission dynamics, the fsTA data of **PT-DPH$_2$**, **TPT-DPH$_3$** and **TPPT-DPH$_4$** were subsequently analysed using a target model in which the population dynamics are constrained by an explicit kinetic network. The model for **PT-DPH$_2$** comprised $S_1 \rightleftarrows {}^1$(TT)→GS for, while those for **TPT-DPH$_3$** and **TPPT-DPH$_4$** comprised $S_1 \rightleftarrows {}^1$(TT)→(T··T). Target analysis provides species-associated spectra (SAS) and microscopic rate constants for each compartment in the kinetic network, allowing the transient spectral signatures to be assigned to specific intermediates, subject to the assumptions of the chosen model.[5]

The sequential analysis of both fsTA and nsTA datasets was used to guide and validate the overall kinetic interpretation. In contrast, target analysis was restricted to the fsTA window, where the relevant population interconversion dynamics are most clearly resolved. The nsTA data for **PT-DPH$_2$** were instead fitted using a non-sequential model.

### 1.8. Estimation of Yields of Triplet-Pair States and Isolated Triplets.

To quantify the degree of triplet-pair formation within the reversible singlet fission (SF) manifold, the early-time dynamics are treated as a two-state interconversion between the initially prepared singlet excited state ($S_1$) and the correlated triplet-pair state ($^1$TT):[5]

$$S_1 \underset{k_{-SF}}{\overset{k_{SF}}{\leftrightarrows}} {}^1(\mathrm{TT}) \qquad \text{(S1)}$$

Here, $k_{SF}$ is the forward rate constant for $S_1 \rightarrow {}^1$(TT), and $k_{-SF}$ is the back reaction rate constant for $^1$(TT) → $S_1$. Under the simplifying approximation that decay of $S_1$ to the ground state is slow compared with the reversible $S_1 \rightleftharpoons {}^1$(TT) equilibration, the equilibrium constant for the SF interconversion is given by:

$$K_{SF} = \frac{[TT]_{eq}}{[S_1]_{eq}} = \frac{k_{SF}}{k_{-SF}} \qquad \text{(S2)}$$

Therefore, the singlet fission yield given by,

$$\phi_{TT} = \frac{[TT]_{eq}}{[TT]_{eq}+[S_1]_{eq}} = \frac{K_{SF}}{K_{SF}+1} \qquad \text{(S3)}$$

For **PT-DPH$_2$**, $k_{SF}$ = 0.0437 ps$^{-1}$ and $k_{-SF}$ = 0.00731 ps$^{-1}$, for **TPT-DPH$_3$**, $k_{SF}$ = 0.0506 ps$^{-1}$ and $k_{-SF}$ = 0.0049 ps$^{-1}$ and for **TPPT-DPH$_4$**, $k_{SF}$ = 0.0477 ps$^{-1}$ and $k_{-SF}$ = 0.0094 ps$^{-1}$. Hence singlet fission is $\phi_{TT}$ = 85.68 % (**PT-DPH$_2$**), $\phi_{TT}$ = 91.19 % (**TPT-DPH$_3$**) and $\phi_{TT}$ = 83.55 % (**TPPT-DPH$_4$**).

Isolated triplet quantum yield was calculated by assuming that absorption cross section of a triplet-pair state is equal to the absorption cross section of two triplets. This is equivalent to treating the singlet fission yields 85.68 % (**PT-DPH$_2$**), 91.19 % (**TPT-DPH$_3$**) and 83.55 % (**TPPT-DPH$_4$**) as equivalent to a peak triplet yield of 171.36 % (**PT-DPH$_2$**), 182.38 % (**TPT-DPH$_3$**) and 167.10 % (**TPPT-DPH$_4$**). The yield of isolated triplets may then be determined by comparing the height of the 'triplet plateau' that is associated with those states to the height of the peak of the triplet/triplet-pair PIA signal. The plateau intensities are 6.0 % for **PT-DPH$_2$**, 49.0 % for **TPT-DPH$_3$** and 44.0 % for **TPPT-DPH$_4$**. This would indicate isolated triplet yields of ~10.28 %, ~ 89.36 % and ~73.52% respectively.

For comparison with the energy-gradient heterotrimer **TPT-DPH$_3$**, the free-triplet yield of the previously reported homotrimer **L-(mDPH)$_3$**,[5] denoted here as **PPP-DPH$_3$**, was estimated using the same triplet-plateau procedure. In the previous analysis of **L-(mDPH)$_3$,** the singlet-fission yield was estimated from the reversible $S_1 \rightleftharpoons {}^1(TT)$ kinetics using $k_{SF}$ = 1/18 ps = 0.056 ps$^{-1}$ and $k_{-SF}$ = 1/100 ps = 0.010 ps$^{-1}$, giving $\Phi_{TT} \approx$ 85%. Treating this 85% triplet-pair yield as an effective peak triplet yield of 170%, and using the same assumption that the absorption cross section of a triplet-pair state is equal to that of two isolated triplets, the isolated-triplet yield was obtained by comparing the long-time triplet plateau with the peak triplet/triplet-pair PIA amplitude. For **PPP-DPH$_3$**, the triplet plateau reaches approximately 40% of the peak amplitude, giving an isolated-triplet yield of approximately 170% × 0.40 = 68%. This value is used as the homotrimer reference yield for comparison with **TPT-DPH$_3$** in the main text.

### 1.9. Magnetic-field-dependent photoluminescence (MagPL) analysis

Raw MagPL data were processed using a custom Python routine. The negative-field branch (−600 to 0 mT) was reflected onto the positive-field axis, exploiting the assumed symmetry PL(B) = PL(−B). Repeated measurements at the same magnetic field were averaged prior to fitting. The PL intensity was normalised to the zero-field value $PL_0$ as

$$\frac{\Delta PL}{PL_0} = \frac{PL(B) - PL_0}{PL_0} \tag{S4}$$

where $PL_0$ is the mean intensity for |B| ≤ 5 mT, or an interpolated value if no experimental point fell within this window.

Normalised traces were fitted over 0–600 mT by nonlinear least squares (lmfit) using a one-component saturating function,[6–8]

$$y(B) = y_0 + A_1 \frac{B^2}{B^2 + B_1^2},$$

was used as the default fitting model. Where the field dependence showed both narrow- and broad-field features, a two-component form,[6–8]

$$y(B) = y_0 + A_1 \frac{B^2}{B^2 + B_1^2} + A_2 \frac{B^2}{B^2 + B_2^2}$$

Here $y_0$ is a field-independent offset, $A_i$ are the component amplitudes (total relative PL change), and $B_i$ are the characteristic half-fields (constrained to positive values). Initial parameters were estimated from the experimental trace. These functions serve as empirical descriptors of the MagPL lineshape and are not intended as mechanistic models of the spin dynamics. All MagPL plots in the manuscript show experimental points alongside continuous best-fit curves of $\Delta PL/PL_0$ obtained by this procedure.

## Section 2: Tables

**Table S1**: Oscillator strength for different transitions of **P-DPH**, and **T-DPH** calculated at TD-b3lyp/cc-pvdz level of theory.

| Molecule | Excitation | | Energy (eV) | Wavelength (nm) |
|---|---|---|---|---|
| | State | Oscillator Strength | | |
| **P-DPH** | $S_1$ | 2.26 | 3.12 | 397.41 |
| | $S_2$ | 0.00 | 3.68 | 337.20 |
| | $S_3$ | 0.15 | 3.92 | 315.90 |
| | $S_4$ | 0.01 | 3.98 | 311.19 |
| **T-DPH** | $S_1$ | 2.78 | 2.91 | 425.11 |
| | $S_2$ | 0.01 | 3.53 | 350.68 |
| | $S_3$ | 0.02 | 3.71 | 334.39 |
| | $S_4$ | 0.05 | 3.90 | 317.70 |

**Table S2**: UV-vis and Fluorescence Spectroscopic Data of **T-DPH, P-DPH, PT-DPH$_2$, TPT-DPH$_3$**, **TPPT-DPH$_4$,** measured in Toluene at Room Temperature.

| | T-DPH | P-DPH | PT-DPH$_2$ | TPT-DPH$_3$ | TPPT-DPH$_4$ |
|---|---|---|---|---|---|
| $\lambda_{Abs}^{max}$, nm | 379.0 | 365.6 | 366.0 | 380.0 | 379.0 |
| $\lambda_{PL}^{max}$, nm | 451.0 | 432.5 | 450.0 | 450.0 | 450.0 |

## Section 3: Figures

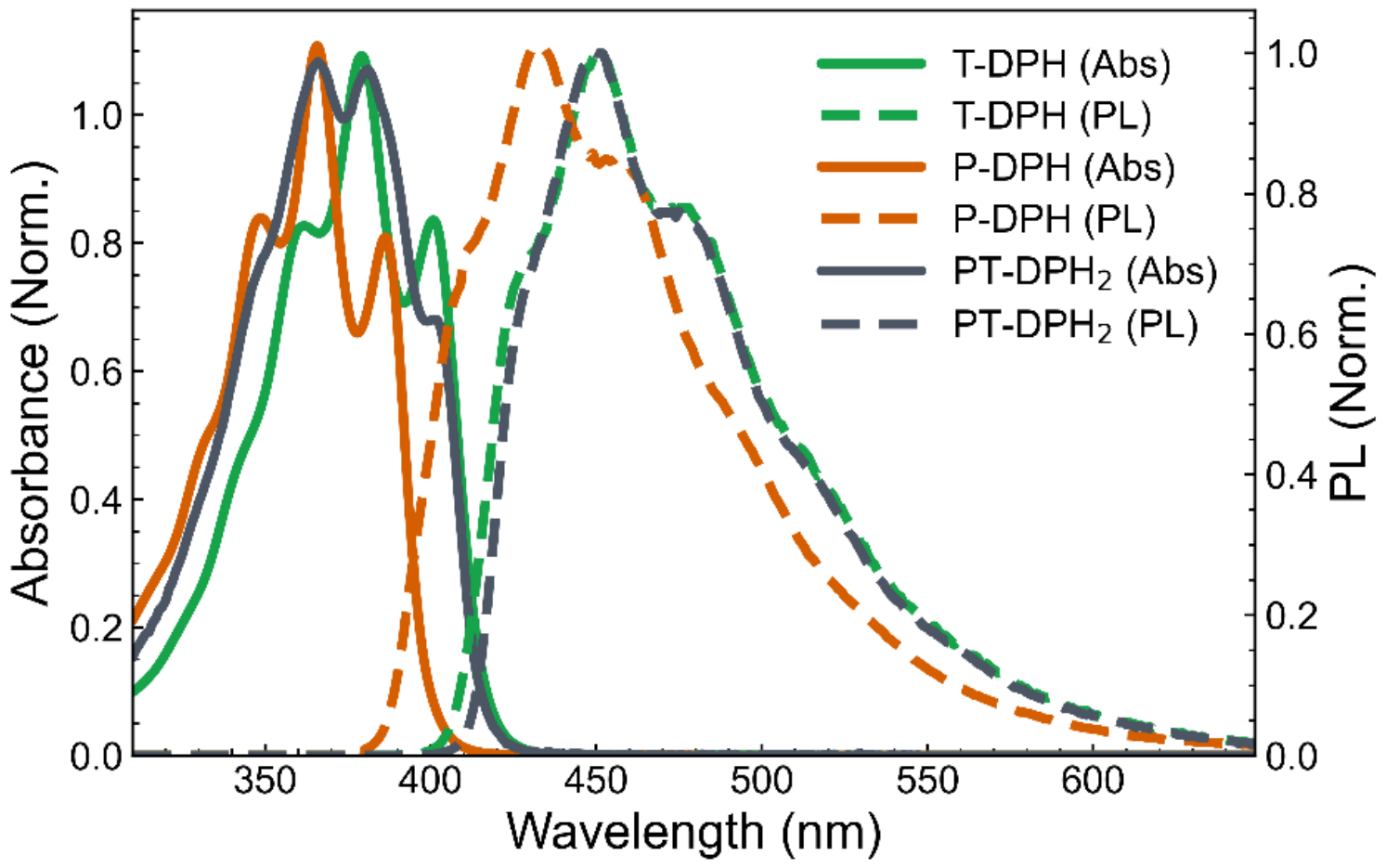


**Figure S1**: Normalized absorption (solid) and PL (dashed) spectra of **T-DPH**, **P-DPH**, and **PT-DPH$_2$** in toluene at room temperature (~15 µM; 1 mm cuvette). PL excitation: 370 nm.

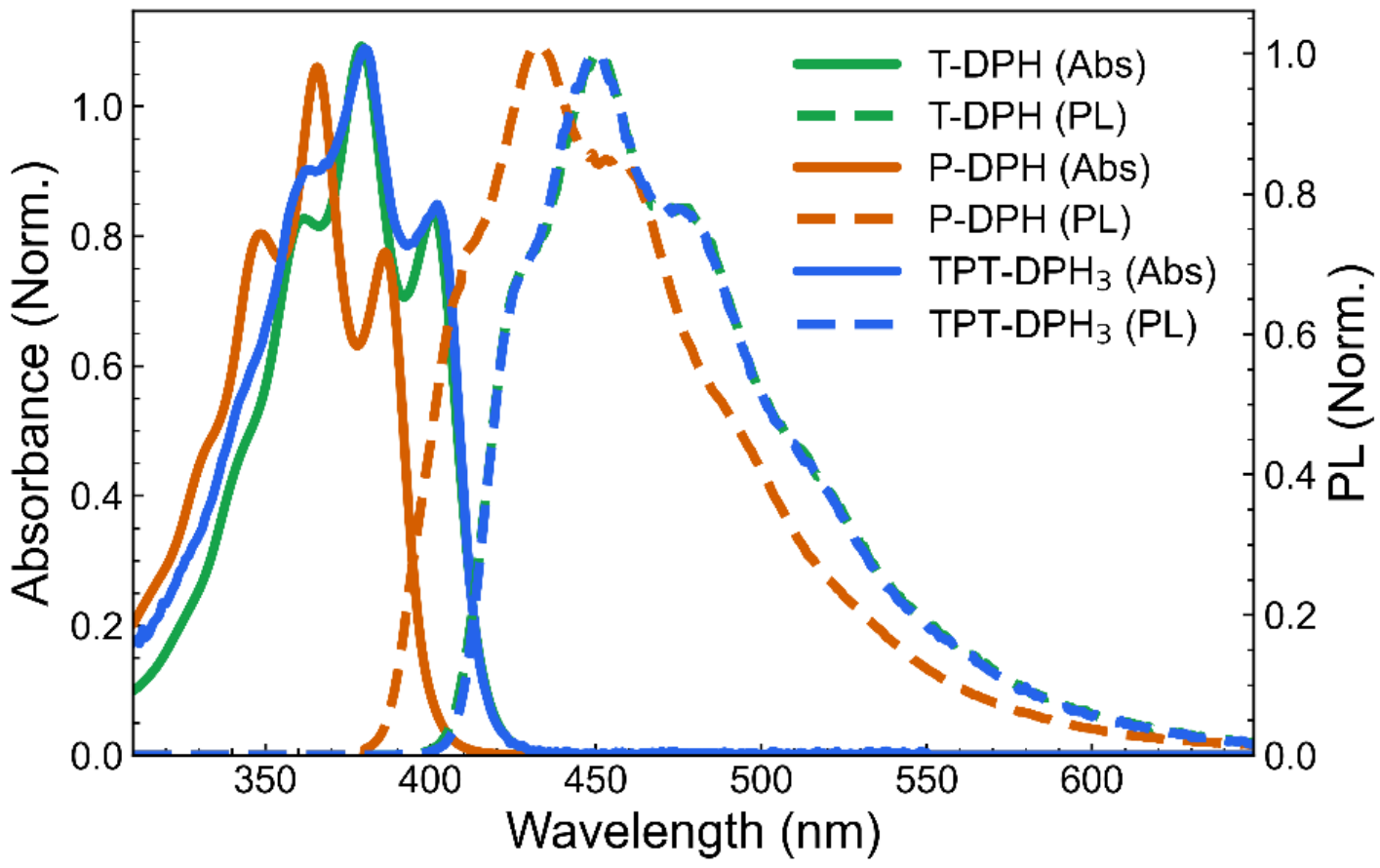


**Figure S2:** Normalized absorption (solid) and PL (dashed) spectra of **T-DPH**, **P-DPH**, and **TPT-DPH$_3$** in toluene at room temperature (~15 µM; 1 mm cuvette). PL excitation: 370 nm.

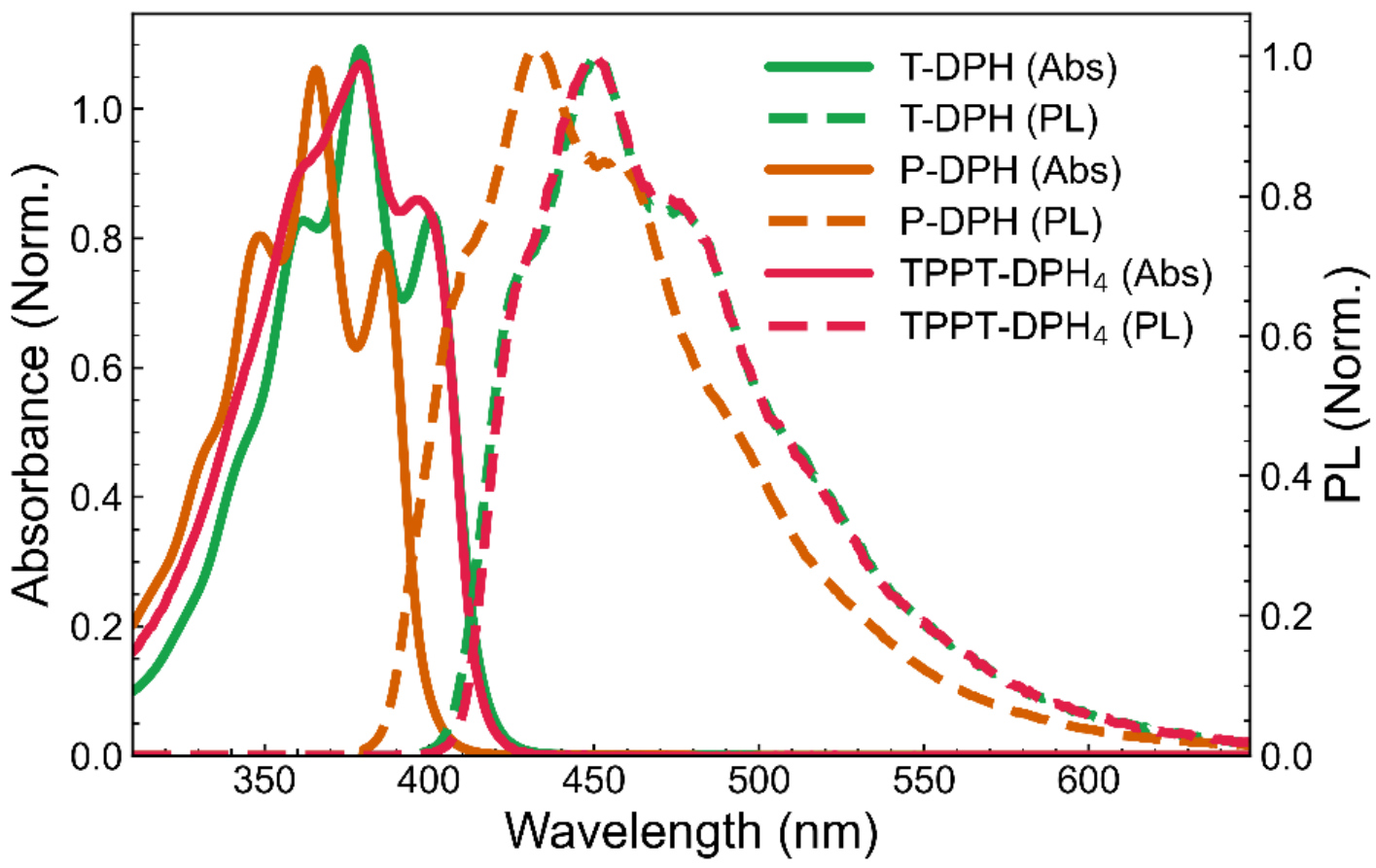


**Figure S3:** Monomer and dimer comparison in dilute solution. Normalized absorption (solid) and PL (dashed) spectra of T-DPH, P-DPH, and TPPT-DPH$_4$ in toluene at room temperature (~15 µM; 1 mm cuvette). PL excitation: 370 nm.

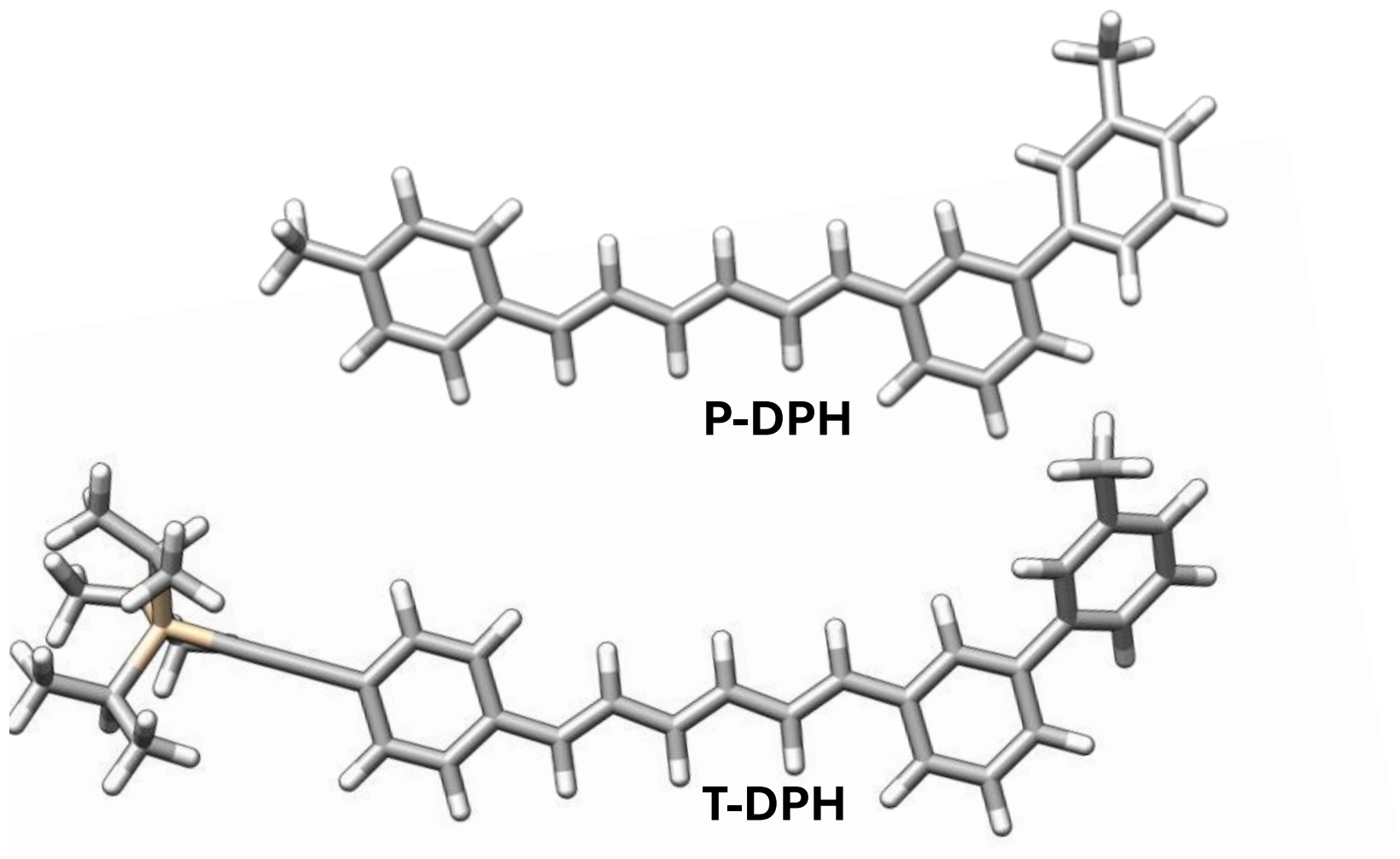


**Figure S4**. Energy-minimized structures of P-DPH (top), and T-DPH (bottom) calculated at the b3lyp/cc-pvdz level of theory.

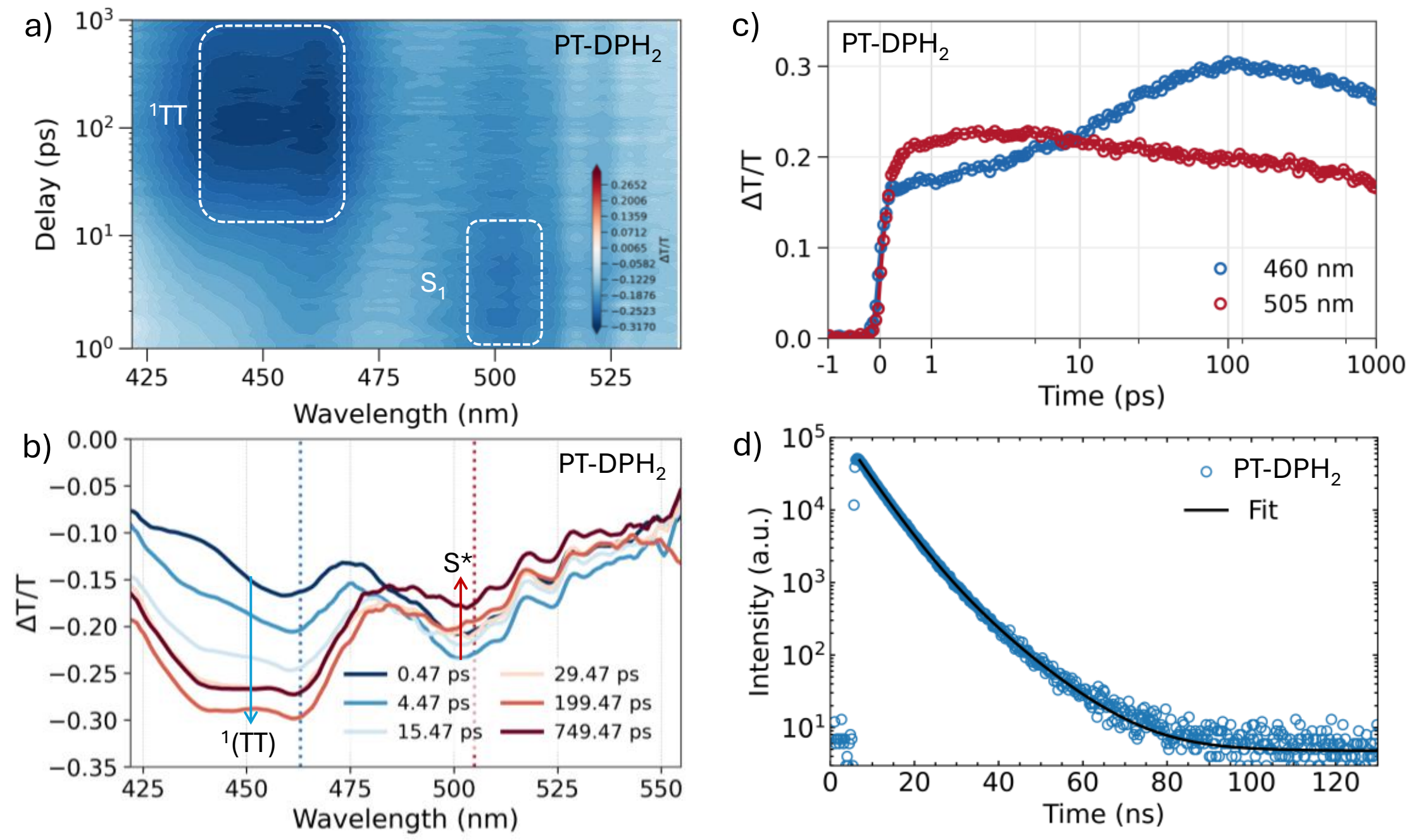


**Figure S5**. a) fsTA map of PT-DPH$_2$ in toluene following 355 nm excitation, showing the early-time singlet manifold (S*) and the growth of the correlated triplet-pair band ($^1$TT). b) Selected fsTA spectra of PT-DPH$_2$ at representative pump–probe delays, illustrating the redistribution of spectral weight from the singlet region into the triplet-pair region. c) kinetic traces for PT-DPH$_2$ extracted from the S* and triplet-pair windows. d) Time-resolved fluorescence decay profiles of PT-DPH$_2$ in dilute toluene solution ($\lambda_{exci}$=375 nm, collection at 450 nm).

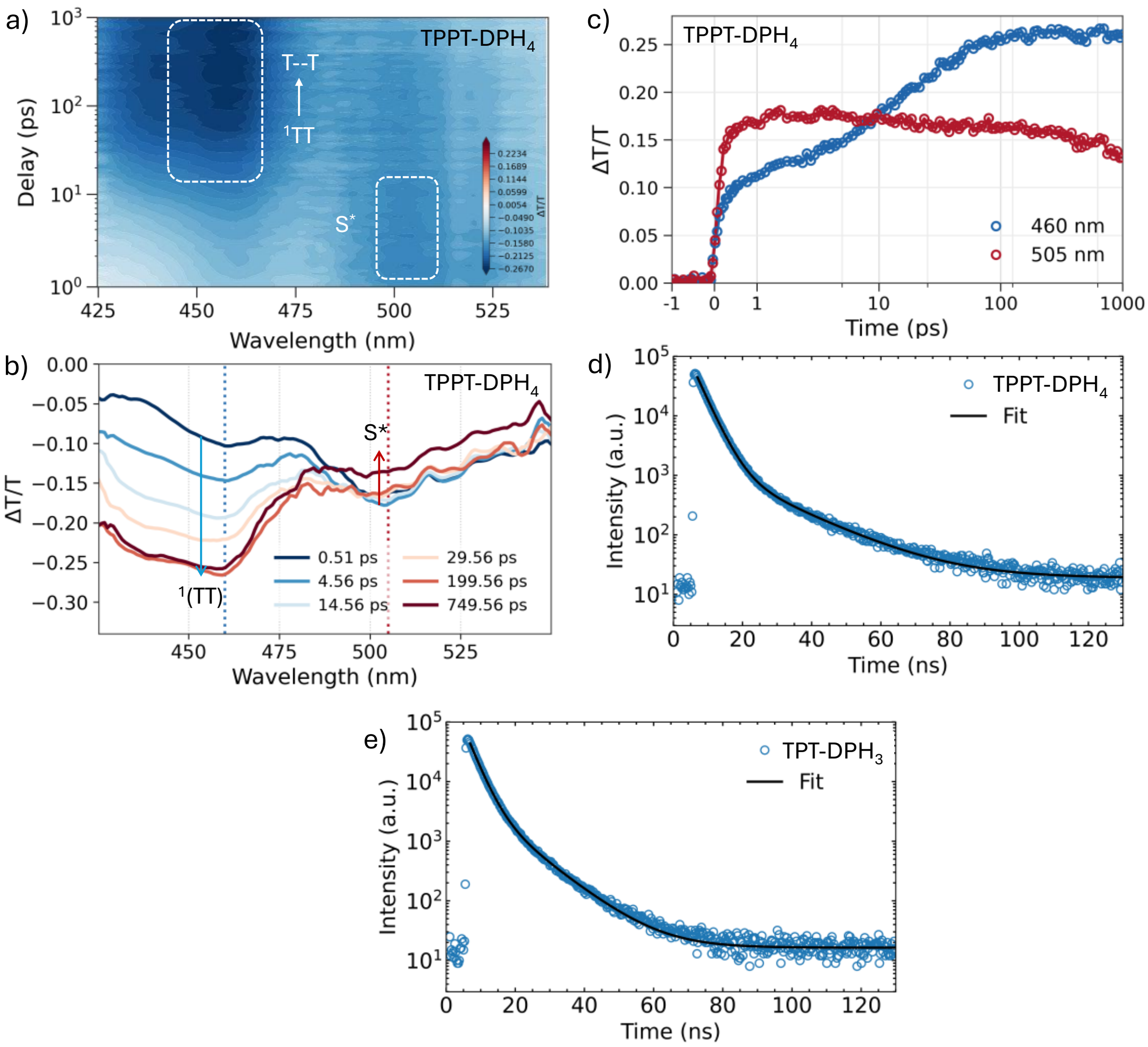


**Figure S6**. Femtosecond transient absorption reveals two-step triplet-pair evolution in TPPT-DPH$_4$ a) fsTA map of TPPT-DPH$_4$ in toluene following 355 nm excitation, showing the early-time singlet manifold (S*) and the growth of the correlated triplet-pair band ($^1$TT), followed by the emergence of more weakly exchange-coupled triplet-pair contribution (T··T). b) Selected fsTA spectra of TPPT-DPH$_4$ at representative pump–probe delays, illustrating the redistribution of spectral weight from the singlet region into the triplet-pair region. c) kinetic traces for TPPT-DPH$_4$ extracted from the S* and triplet-pair windows. Time-resolved fluorescence decay profiles of TPPT-DPH$_4$ (d) and TPT-DPH$_3$ (e) in dilute toluene solution ($\lambda_{exci}$=375 nm, collection at 450 nm).

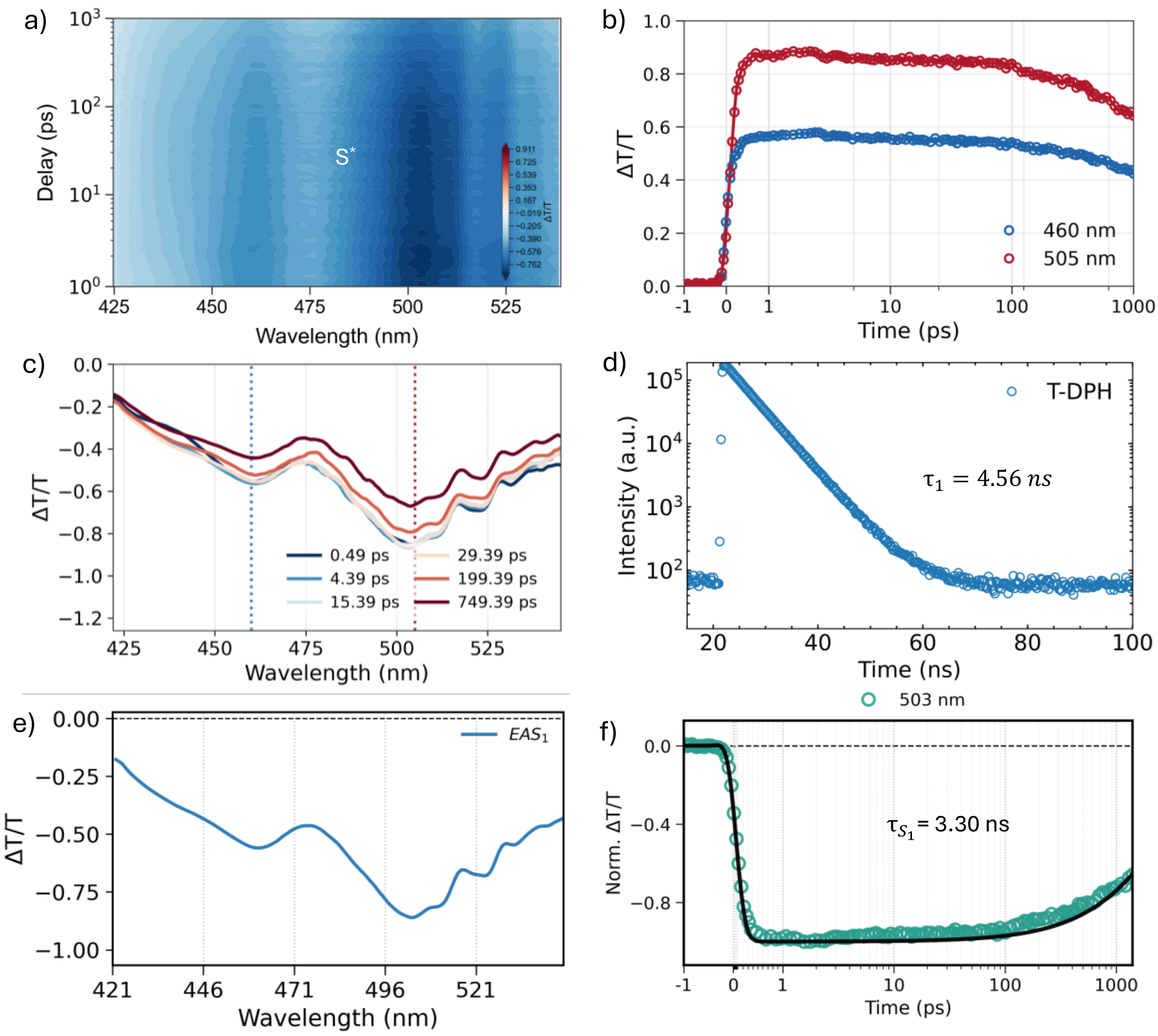


**Figure S7.** a) fsTA map of T-DPH in toluene following 355 nm excitation, showing the early-time singlet manifold (S*). b) Kinetic traces for T-DPH extracted from 460 nm and 505 nm. c) Selected fsTA spectra of T-DPH at representative pump–probe delays, illustrating the redistribution of spectral weight from the singlet region. d) Time-resolved fluorescence decay profiles of T-DPH in dilute toluene solution ($\lambda_{exci}$=375 nm, collection at 450 nm). e) Evolution-associated difference spectra (EADS) obtained from the global analysis for the $S_1$. f) Representative transient absorption kinetic traces at selected wavelengths, shown as symbols, together with the corresponding global fits shown as solid lines.

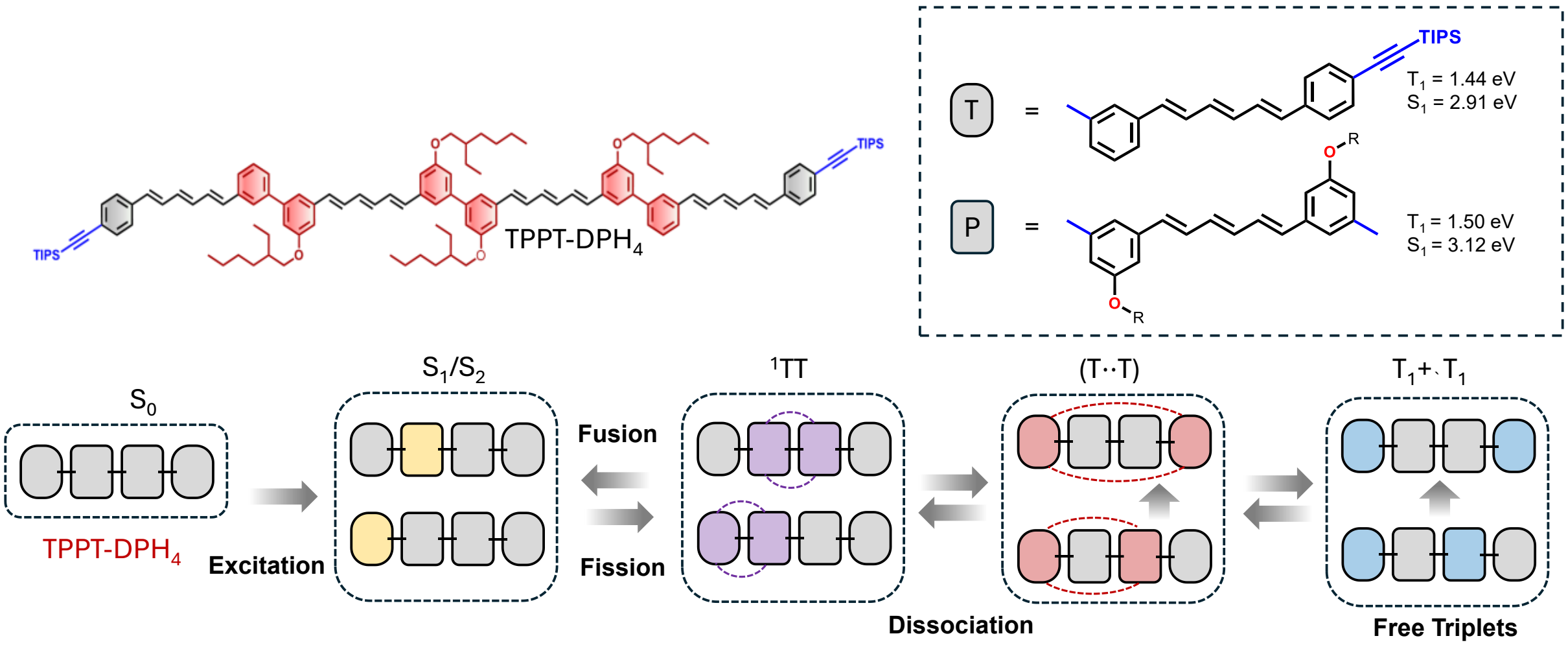


**Figure S8**: Schematic representation of triplet-pair evolution in TPPT-DPH$_4$. Following initial $^1$(TT) formation, triplet population can access multiple local triplet-pair configurations, including T–P and P–P arrangements, before reaching the terminally separated (T··T) state. This broader configurational landscape enables triplet-pair separation relative to the dimer but also introduces competing redistribution and recombination pathways that reduce the free-triplet yield relative to TPT-DPH$_3$.

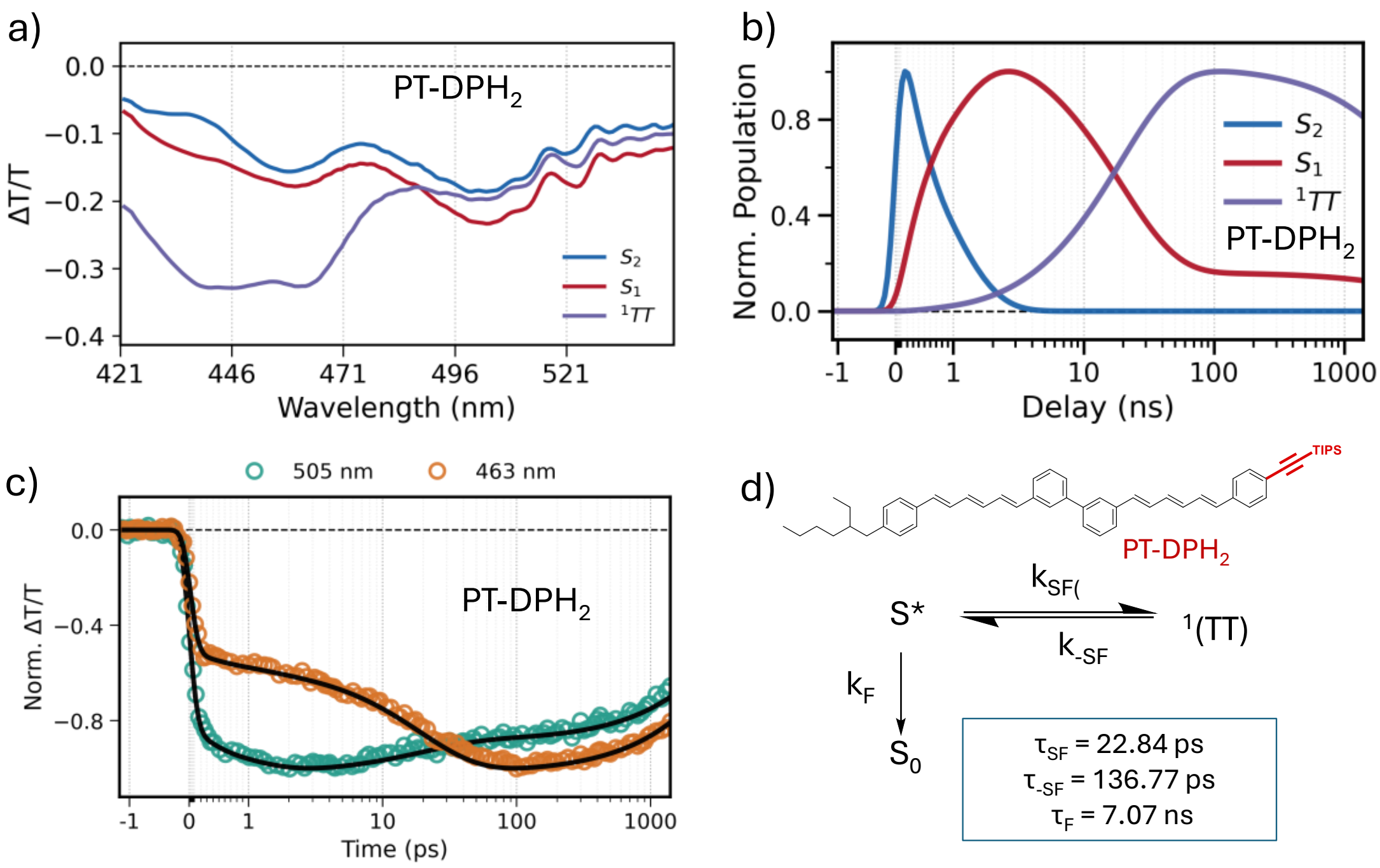


**Figure S9. Global target analysis of the transient absorption data of PT-DPH$_2$.** a) Species-associated difference spectra (SADS) obtained from the target analysis for the $S_2$, $S_1$, and $^1TT$ states. In the manuscript, the $S_2$ and $S_1$ states are collectively referred to as the $S^*$ state. b) Time-dependent population profiles of the corresponding states derived from the target-analysis model. c) Representative transient absorption kinetic traces at selected wavelengths, shown as symbols, together with the corresponding global fits shown as solid lines. d) Kinetic model used for the global analysis of PT-DPH$_2$.

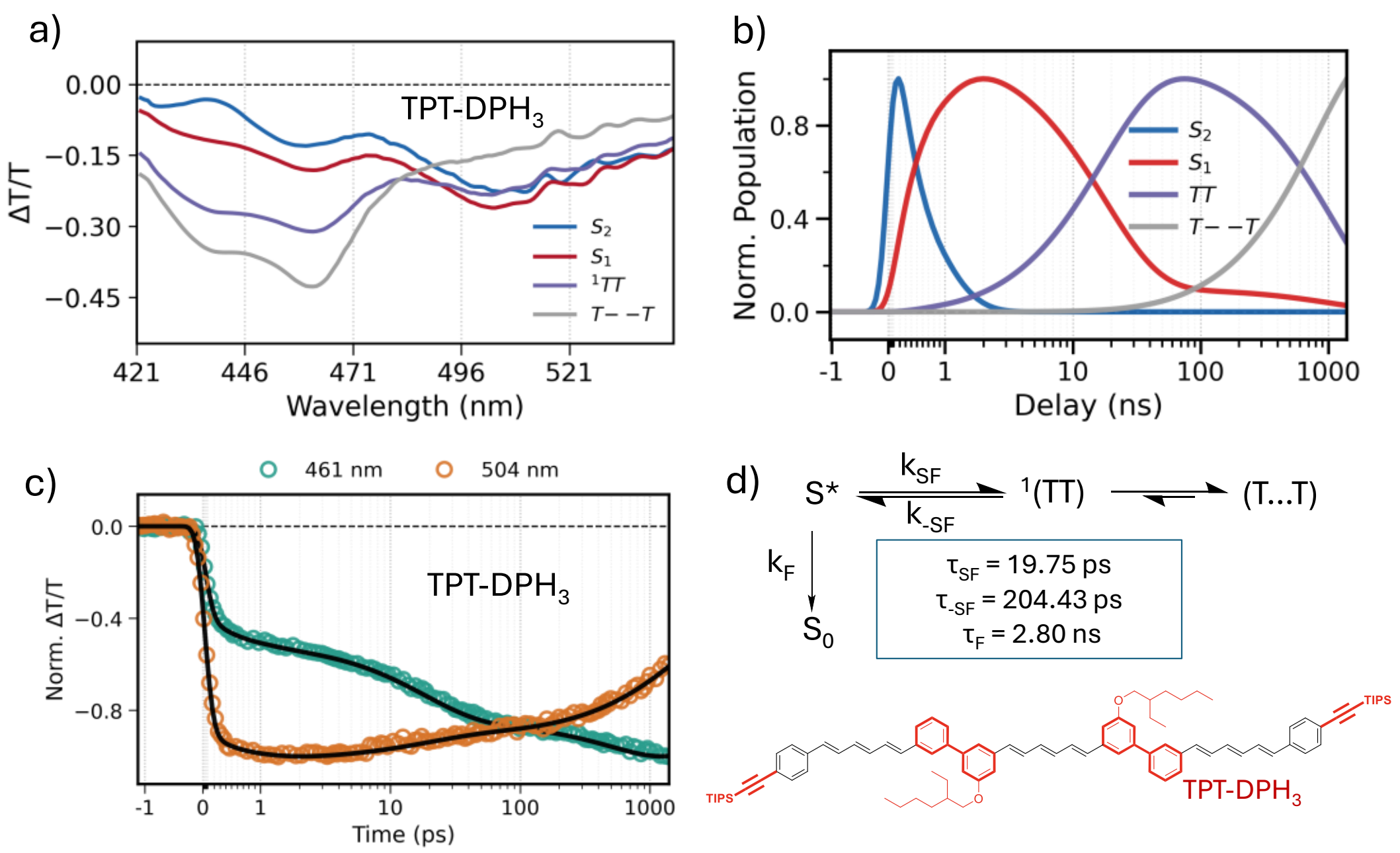


**Figure S10. Global target analysis of the transient absorption data of TPT-DPH₃.** a) Species-associated difference spectra (SADS) obtained from the target analysis for the $S_2$, $S_1$, and $^1TT$ states. In the manuscript, the $S_2$ and $S_1$ states are collectively referred to as the $S*$ state. b) Time-dependent population profiles of the corresponding states derived from the target-analysis model. c) Representative transient absorption kinetic traces at selected wavelengths, shown as symbols, together with the corresponding global fits shown as solid lines. d) Kinetic model used for the global analysis of TPT-DPH$_3$.

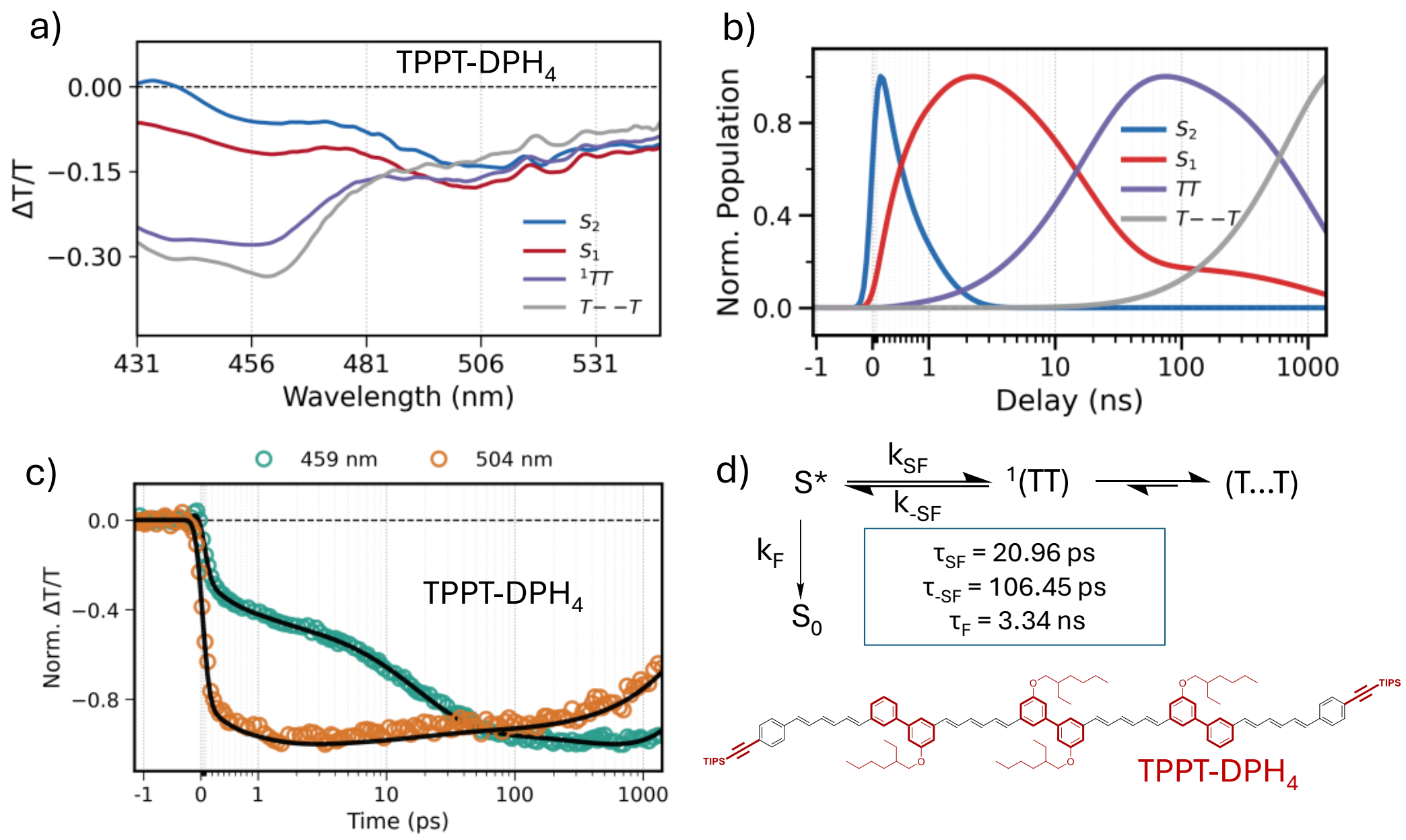


**Figure S11. Global target analysis of the transient absorption data of TPPT-DPH₄.** a) Species-associated difference spectra (SADS) obtained from the target analysis for the $S_2$, $S_1$, and $^1TT$ states. In the manuscript, the $S_2$ and $S_1$ states are collectively referred to as the $S*$ state. b) Time-dependent population profiles of the corresponding states derived from the target-analysis model. c) Representative transient absorption kinetic traces at selected wavelengths, shown as symbols, together with the corresponding global fits shown as solid lines. d) Kinetic model used for the global analysis of TPPT-DPH$_4$.

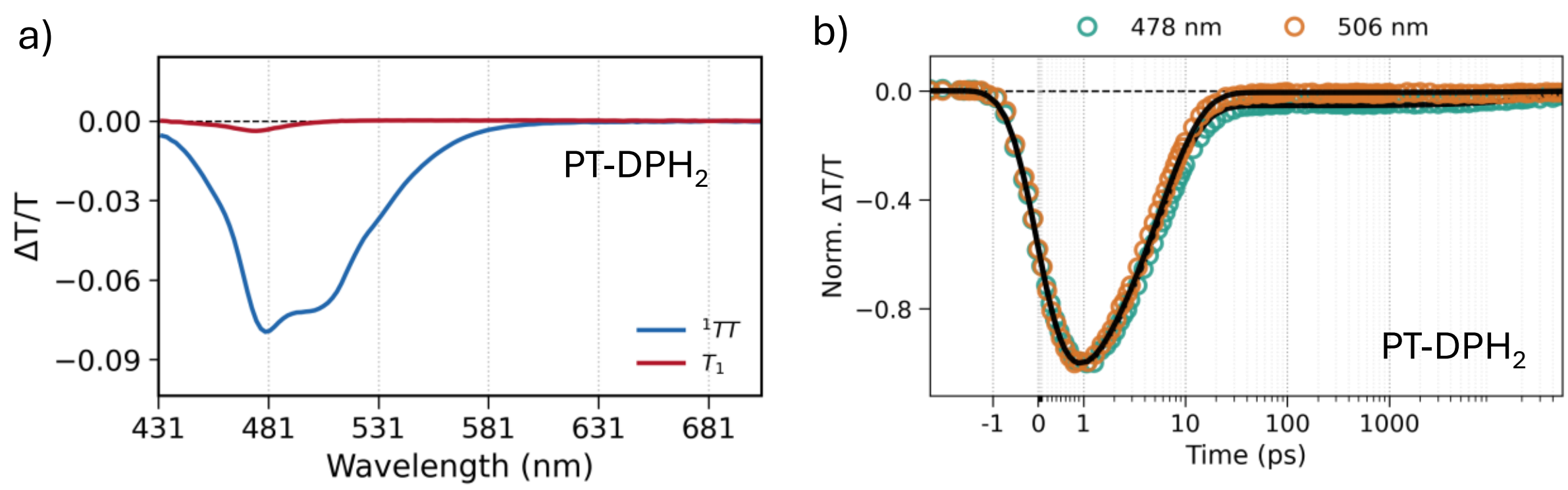


**Figure S12: Global analysis of nanosecond transient absorption data of PT-DPH$_2$.** a) Decay-associated difference spectra (DADS) obtained from the global analysis using non-sequential model. b) Representative transient absorption kinetic traces at selected wavelengths, shown as symbols, together with the corresponding global fits shown as solid lines.

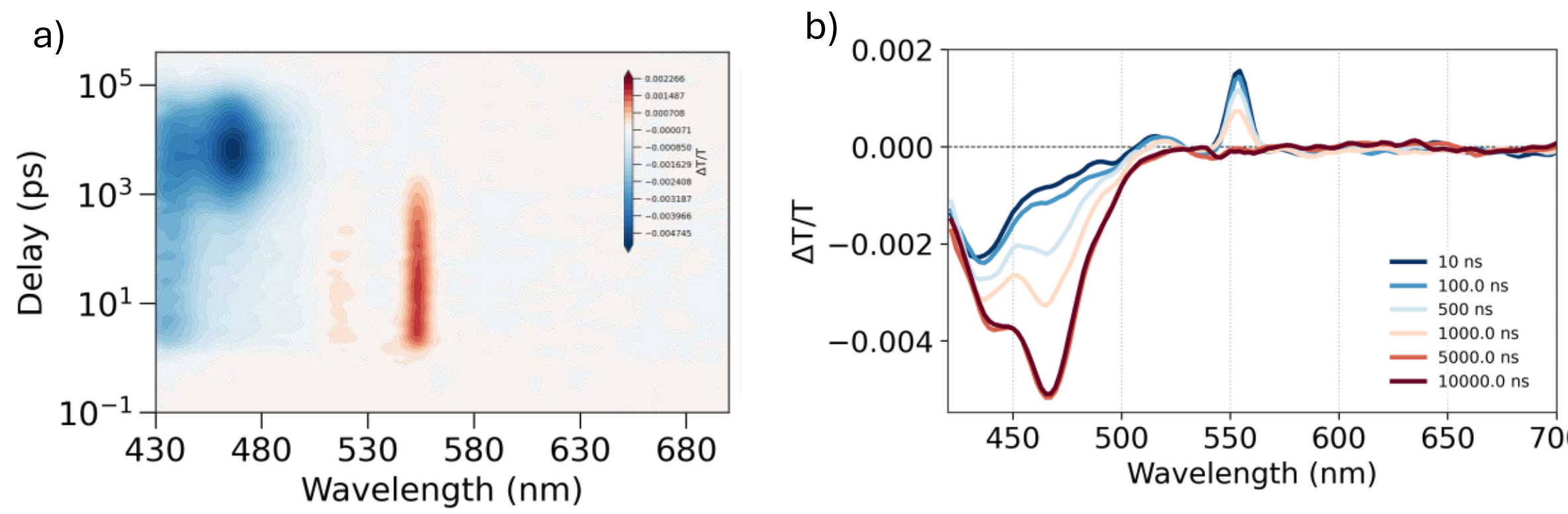


**Figure S13:** Nanosecond transient absorption (nsTA) data of sensitized triplet T-DPH in toluene following 532 nm excitation (500 µM T-DPH with 120 µM PdOEP).

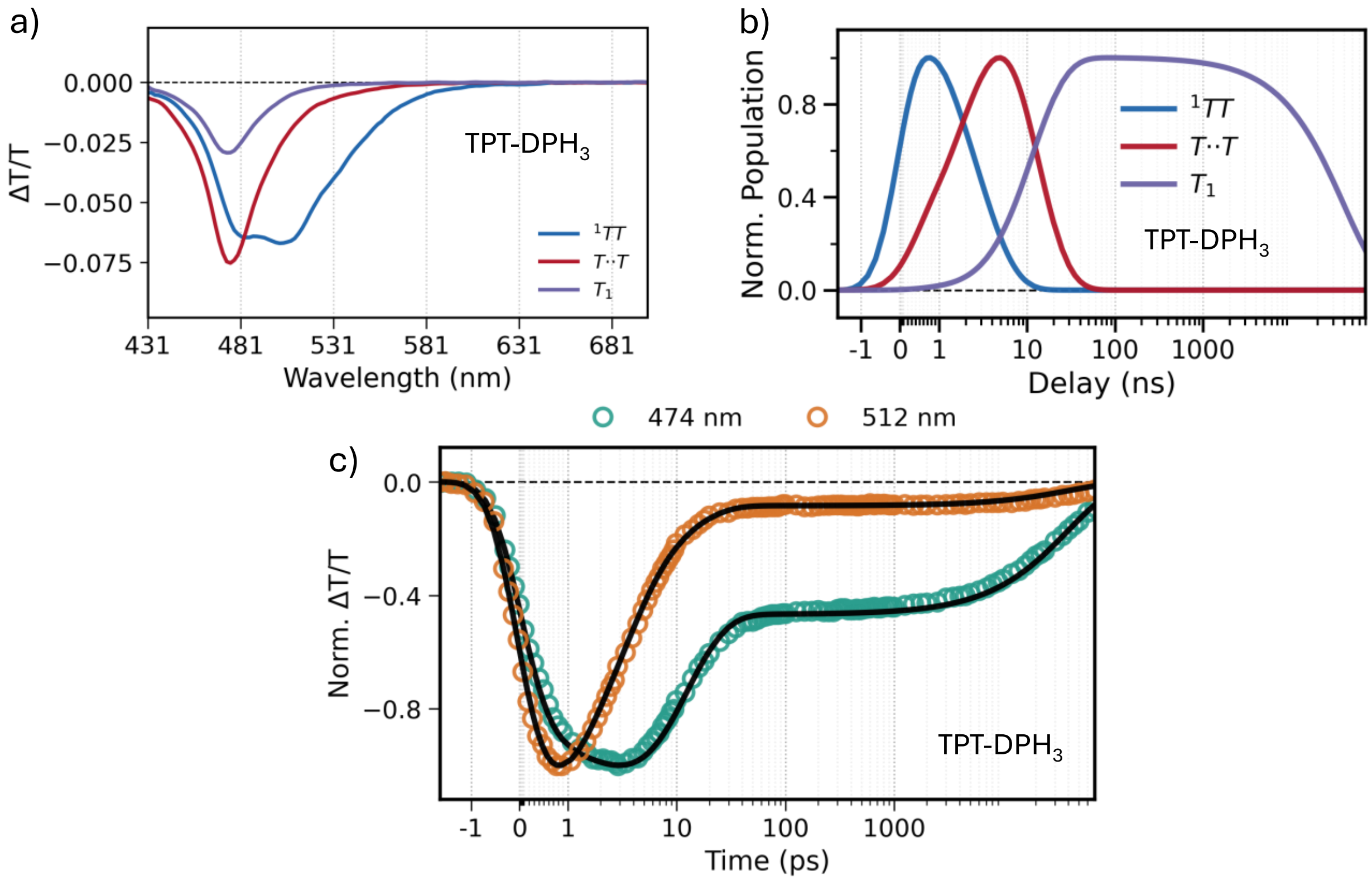


**Figure S14: Global analysis of nanosecond transient absorption data of TPT-DPH$_3$.** a) Evolution-associated difference spectra (EADS) obtained from the global analysis using sequential model. b) Time-dependent population profiles of the corresponding states derived from the target-analysis model. c) Representative transient absorption kinetic traces at selected wavelengths, shown as symbols, together with the corresponding global fits shown as solid lines.

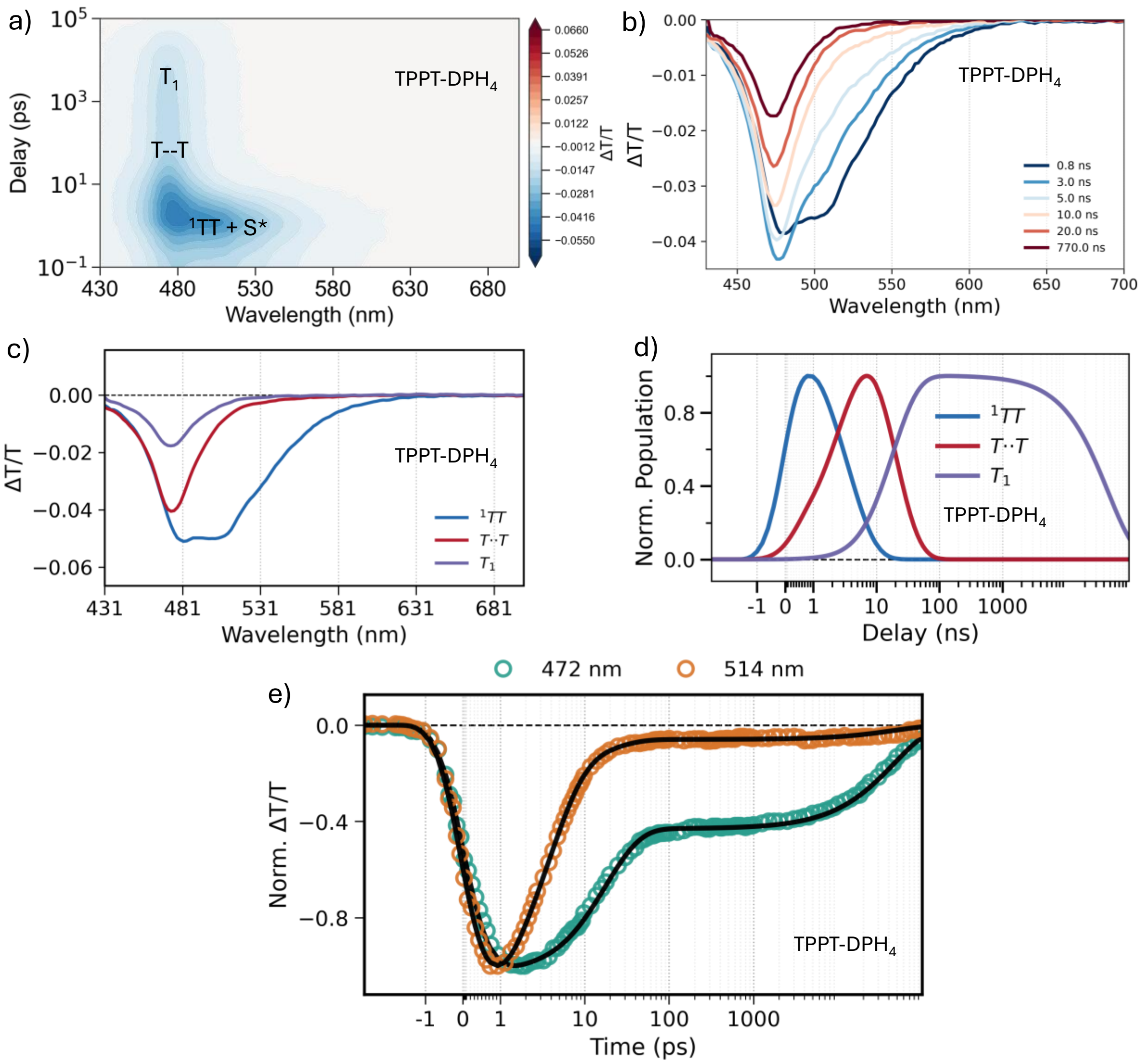


**Figure S15: Nanosecond transient absorption (nsTA) data and corresponding global and target analysis of TPPT-DPH4.** (a) nsTA map of TPPT-DPH4 (tetramer) in toluene following 355 nm excitation. (b) Selected nsTA spectra at representative delay times for the tetramer. (c) Evolution-associated difference spectra (EADS) obtained from the global analysis using a sequential model. (d) Time-dependent population profiles of the corresponding states derived from the target-analysis model. (e) Representative transient absorption kinetic traces at selected wavelengths, shown as symbols, together with the corresponding global fits shown as solid lines.

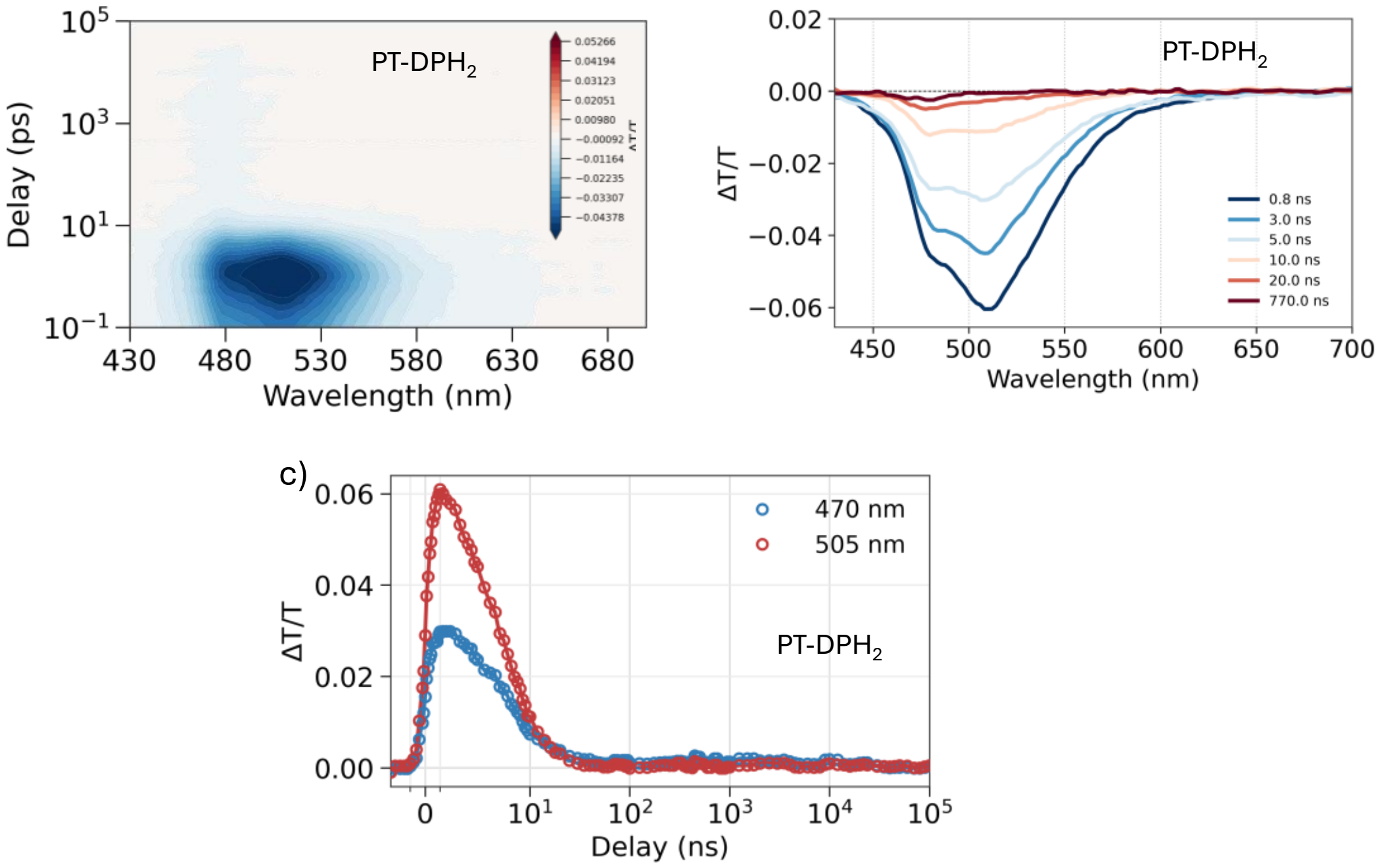


**Figure S16: Nanosecond transient absorption (nsTA) data of PT-DPH2 in toluene at room temperature under a magnetic field of 110 mT.** (a) nsTA map of PT-DPH$_2$ in toluene following 355 nm excitation. (b) Selected nsTA spectra at representative delay times for the PT-DPH$_2$. (c) Representative transient absorption kinetic traces at selected wavelengths, shown as symbols, together with the corresponding global fits shown as solid lines.

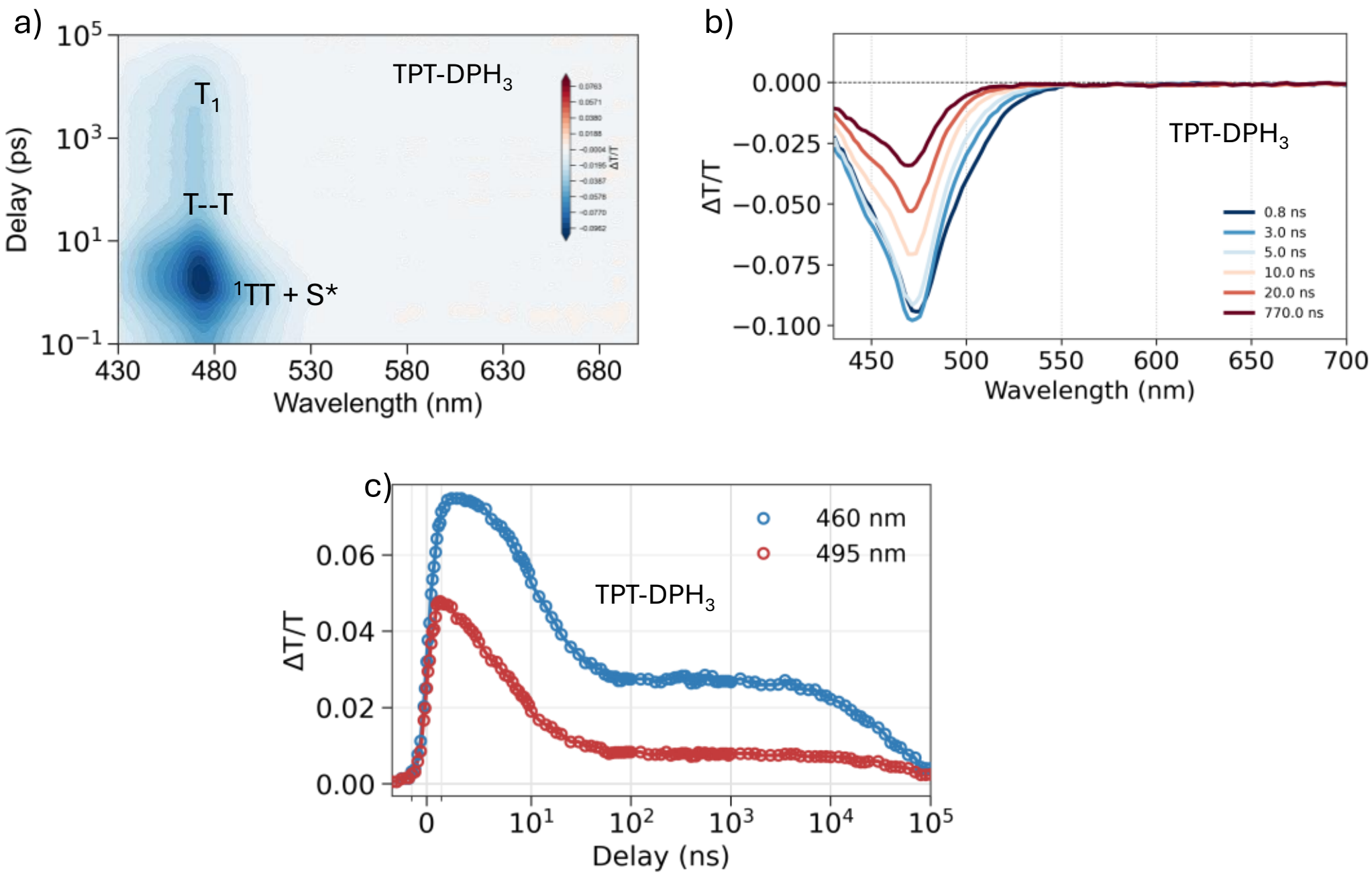


**Figure S17: Nanosecond transient absorption (nsTA) data of TPT-DPH$_3$ in toluene at room temperature under a magnetic field of 110 mT.** (a) nsTA map of TPT-DPH$_3$ in toluene following 355 nm excitation. (b) Selected nsTA spectra at representative delay times for the TPT-DPH$_3$. (c) Representative transient absorption kinetic traces at selected wavelengths, shown as symbols, together with the corresponding global fits shown as solid lines.

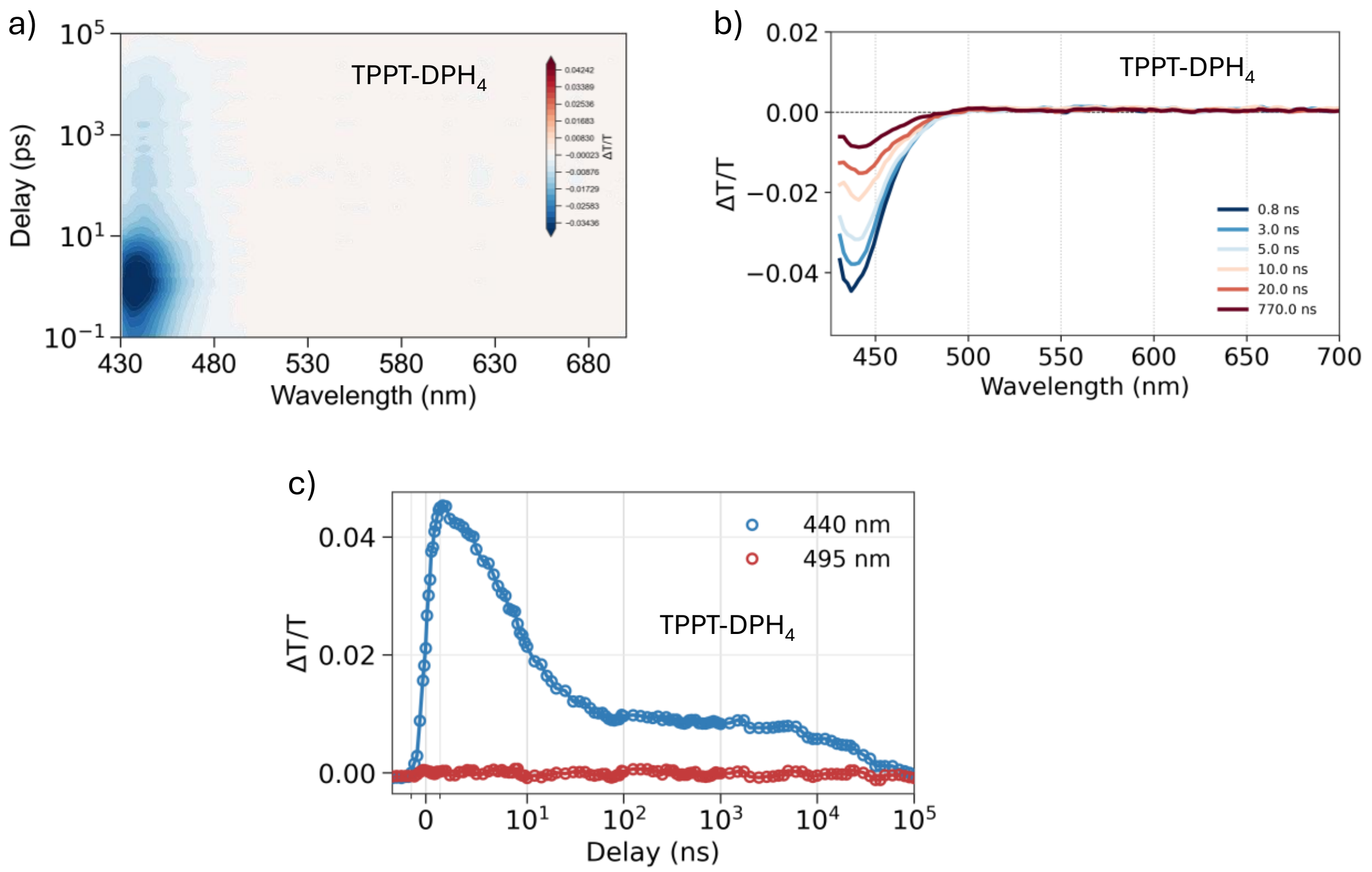


**Figure S18: Nanosecond transient absorption (nsTA) data of TPPT-DPH4 in toluene at room temperature under a magnetic field of 110 mT.** (a) nsTA map of TPPT-DPH4 in toluene following 355 nm excitation. (b) Selected nsTA spectra at representative delay times for the TPPT-DPH4. (c) Representative transient absorption kinetic traces at selected wavelengths, shown as symbols, together with the corresponding global fits shown as solid lines.

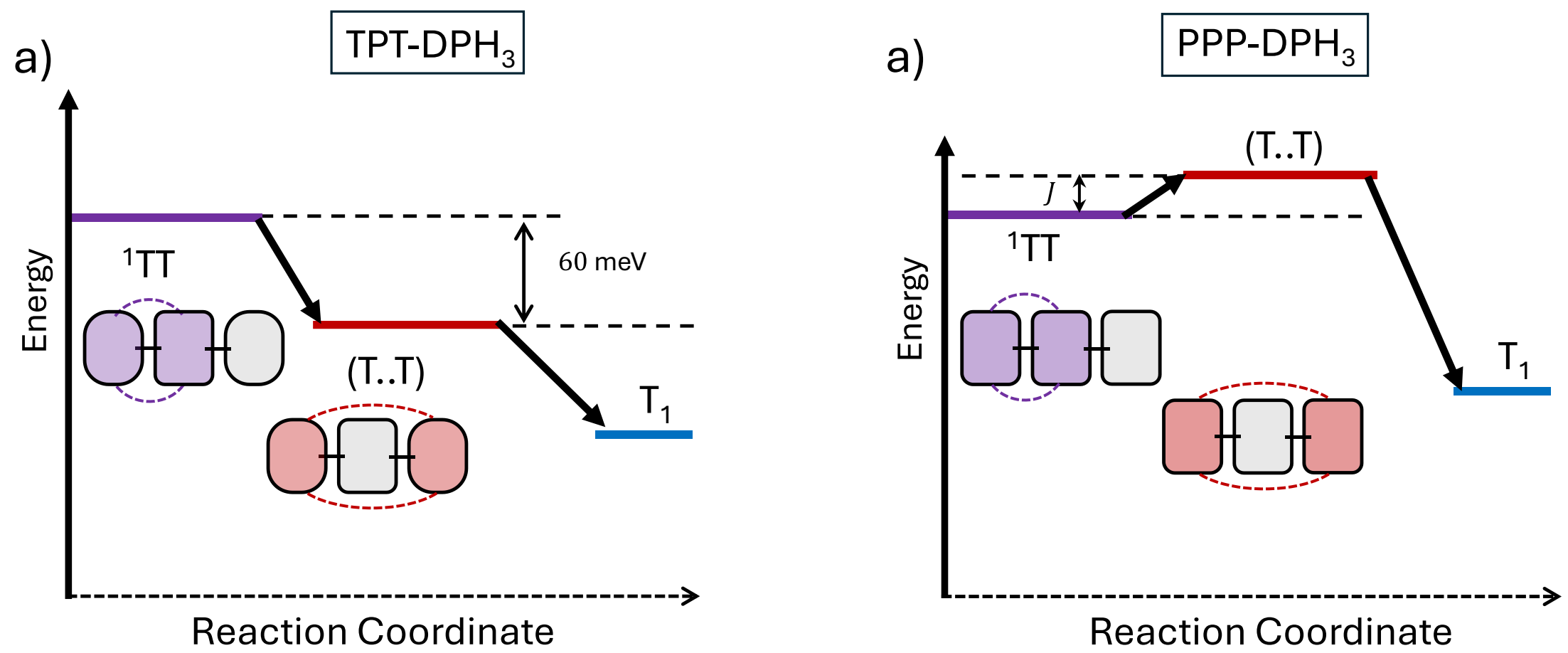


**Figure S19:** Schematic illustration of the proposed excited-state pathways in the TPT-DPH3 and PPP-DPH3. The left and right panels compare the evolution of the initially generated excited state into intermediate spin-correlated states and the final triplet-product state.

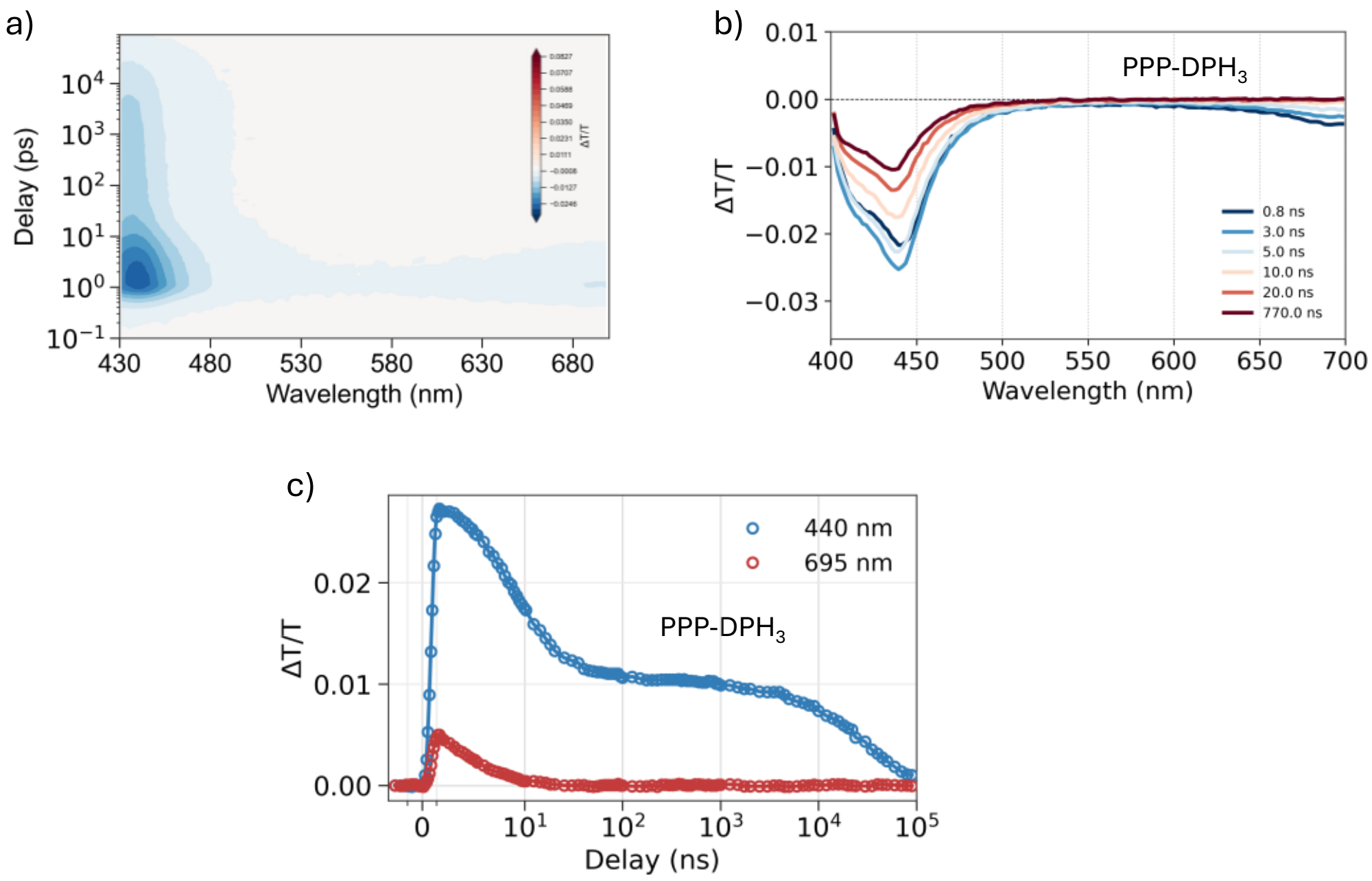


**Figure S20**. **Nanosecond transient absorption (nsTA) data of PPP-DPH$_3$.** (a) nsTA map of PPP-DPH$_3$ (homo-trimer) in toluene following 355 nm excitation. (b) Selected nsTA spectra at representative delay times for the PPP-DPH$_3$. (c) Representative transient absorption kinetic traces at selected wavelengths, shown as symbols, together with the corresponding global fits shown as solid lines. PPP-DPH$_3$ has been reported previously; see ref 5.

## Section 4: Syntheses and Characterisation

Tetraethyl but-2-ene-1,4-diyl(E)-bis(phosphonate),[9] 2-(3-((1E,3E,5E)-6-(4-(2-ethylhexyl)phenyl)hexa-1,3,5-trien-1-yl)phenyl)-4,4,5,5-tetramethyl-1,3,2-dioxaborolane[5] and P-DPH[10] were prepared as previously reported.

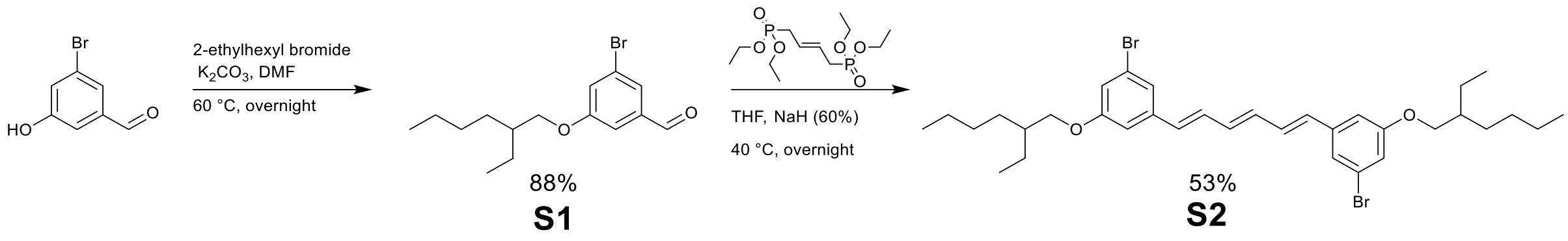


**Scheme S1:** Synthesis of the S1 and S2.

### 4.1. Synthesis of 3-bromo-5-((2-ethylhexyl)oxy)benzaldehyde (S1):

3-bromo-5-hydroxybenzaldehyde (3.00 g, 15 mmol, 1 eq.), K2CO3 (4.14 g, 30 mmol, 2 eq.), and DMF (45 mL) were added to a dried 250 mL flask. 2-ethylhexyl bromide (4.35 g, 22.5 mol, 1.5 eq.) was added and the reaction was heated to 60 °C overnight. The reaction solution was slowly poured into 1 M dilute hydrochloric acid solution, then diluted with DCM (~75 mL) and brine (~50 mL). The organic layer was separated and washed with 0.05 M hydrochloric acid (3 x 100 mL), dried ($MgSO_4$) and the solvent removed *in vacuo*. The crude material was then purified by flash column chromatography (eluent: DCM/ *n*-hexane gradient from 0:100 → 10:90 v/v) to obtain the product as a colourless oil (4.13 g, 88%).

**S1**

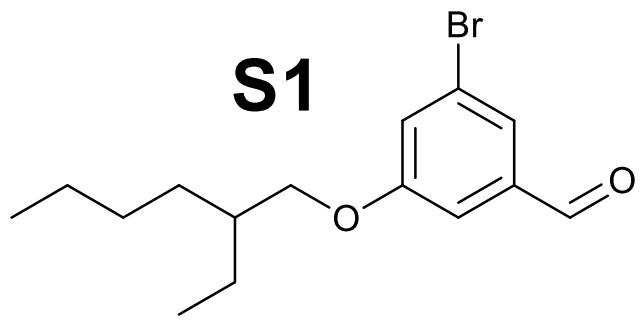

**¹H NMR** (500 MHz, $CDCl_3$) δ 9.90 (s, 1H), 7.56 (t, *J* = 1.5 Hz, 1H), 7.31 (m, 2H), 3.88 (dd, *J* = 5.7, 2.0 Hz, 2H), 1.78 – 1.68 (m, 1H), 1.53 – 1.23 (m, 8H), 0.99 – 0.84 (m, 6H).

### 4.2. Synthesis of (1E,3E,5E)-1,6-bis(3-bromo-5-((2-ethylhexyl)oxy)phenyl)hexa-1,3,5-triene (S2):

To an oven-dried flask under argon was added tetraethyl but-2-ene-1,4-diyl(E)-bis(phosphonate) (250 mg, 0.77 mmol, 1 eq.) and anh. THF (10 mL). Sodium hydride (74 mg, 1.85 mmol, 2.4 eq.) was then added, and the resulting mixture was allowed to stir at RT for 30 mins (cloudy grey mixture). To this, a pre-dissolved solution of 3-bromo-5-((2-ethylhexyl)oxy)benzaldehyde (600 mg, 1.90 mmol, 2.5 eq.) in anh. THF (10 mL), was added dropwise over 5 mins and then left to stir for 30 mins at RT. The mixture was then heated to 40 °C and left to stir for 24 h. The resulting emulsion was poured over ice water (60 mL), and acidified with 1M HCl (3 mL). The mixture was extracted with DCM and the organic phases were separated and dried over $MgSO_4$, and concentrated *in vacuo*. The yellow oil was dry loaded onto silica and purified *via* column chromatography (*n*-hexane). The fractions containing product were combined and concentrated *in vacuo,* to give the product as viscous yellow oil (264 mg, 53%).

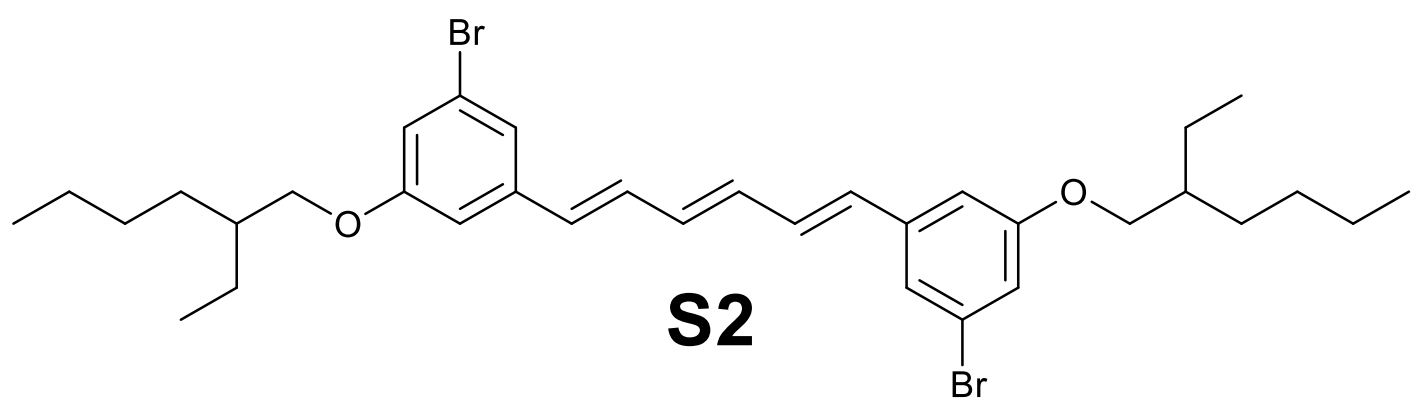


**S2**

**$^{1}$H NMR** (500 MHz, $CDCl_3$) δ 7.14 (t, *J* = 1.5 Hz, 2H), 6.92 (t, *J* = 2.0 Hz, 2H), 6.89 – 6.74 (m, 4H), 6.58 – 6.40 (m, 4H), 3.83 (dd, *J* = 5.7, 2.5 Hz, 4H), 1.75 – 1.64 (m, 2H), 1.59 – 1.25 (m, 16H), 0.98 – 0.84 (m, 12H).

**$^{13}$C NMR** (126 MHz, $CDCl_3$) δ 160.4, 140.2, 134.1, 131.8, 130.5, 123.2, 121.7, 116.9, 111.7, 70.9, 39.5, 30.6, 29.2, 24.0, 23.2, 14.2, 11.3.

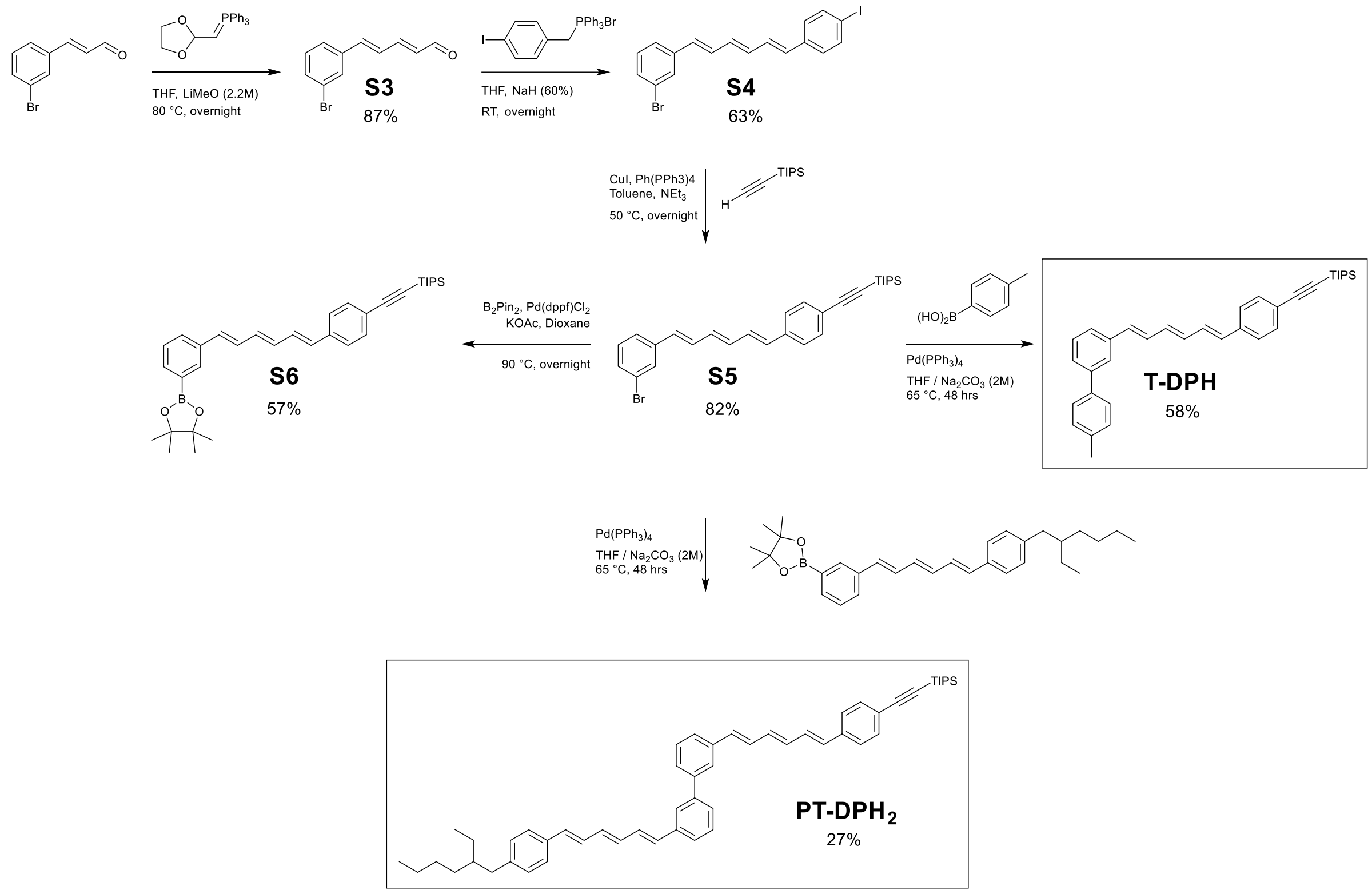


**Scheme S2:** Synthesis of the T-DPH and PT-$DPH_2$.

### 4.3. Synthesis of (2E,4E)-5-(3-bromophenyl)penta-2,4-dienal (S3):

To an oven-dried flask under an argon atmosphere and equipped with a dropping funnel and reflux condenser was added (1,3-dioxolan-2-ylmethyl)triphenylphosphonium bromide (37.0 g, 86.0 mmol, 2.6 eq.), and anh. THF (~200 mL) via cannula. To this was added lithium methoxide solution in methanol (2.2 M, 37.5 mL, 82.5 mmol, 2.5 eq.). The suspension was heated to reflux (80 °C) for 30 mins. The suspension was cooled to RT to this was added (E)-3-(3-bromophenyl)acrylaldehyde (7.00 g, 33 mmol, 1 eq.). The mixture was heated to reflux and allowed to stir for 24 h. The reaction was cooled to RT, at which point 10% aq. HCl solution (100 mL) was added. Stirring was continued for 1 hr to hydrolyse intermediate acetals to the all-trans aldehydes. Colour change observed was from cloudy cream to clear and pale yellow. The organic layer was then

separated, and the aqueous layer extracted with DCM. Combined organic layers were subsequently washed with water, sat. aq. sodium bicarbonate solution and brine, before being dried (MgSO4) and concentrated in vacuo to give the product as a yellow oil, (6.90 g, 87%).

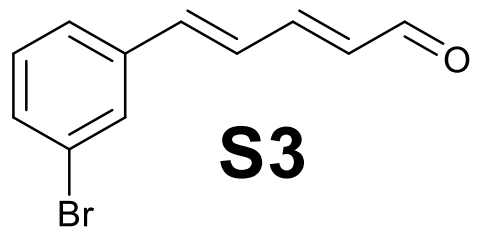


**S3**

**$^1$H NMR** (500 MHz, $CDCl_3$) δ 9.63 (d, *J* = 7.8 Hz, 1H), 7.64 (t, *J* = 1.8 Hz, 1H), 7.46 (ddd, *J* = 7.9, 1.9, 1.0 Hz, 1H), 7.44 – 7.38 (m, 1H), 7.30 – 7.18 (m, 2H), 7.04 – 6.85 (m, 2H), 6.29 (dd, *J* = 15.2, 7.9 Hz, 1H).

**4.4. Synthesis of 1-bromo-3-((1E,3E,5E)-6-(4-iodophenyl)hexa-1,3,5-trien-1-yl)benzene (S4):**

Sodium hydride (60 % dispersion in mineral oil, 940 mg, 23.4 mmol, 1.85 eq.) was suspended in dry THF (60 mL) and cooled to 0 °C using an ice bath, under argon. Under a positive flow of argon 4-iodobenzyl triphenylphosphonium bromide (8.8 g, 15.8 mmol, 1.25 eq.) was added as a single solid portion. The ice bath was removed, and the suspension was stirred at RT for 15 mins. Colour change from the immediately formed yellow suspension to orange was observed. A solution of **S3** (3.0 g, 12.7 mmol, 1.0 eq.) in dry THF (60 mL) was prepared and added dropwise and the mixture stirred overnight, in the dark. The mixture was poured over ice/water, acidified with 0.1 M HCl (25 mL) the resulting precipitate collected via filtration. The solid material was recrystalised from chloroform, filtered, dried under suction. The product was obtained as a yellow needle (3.47 g, 63%).

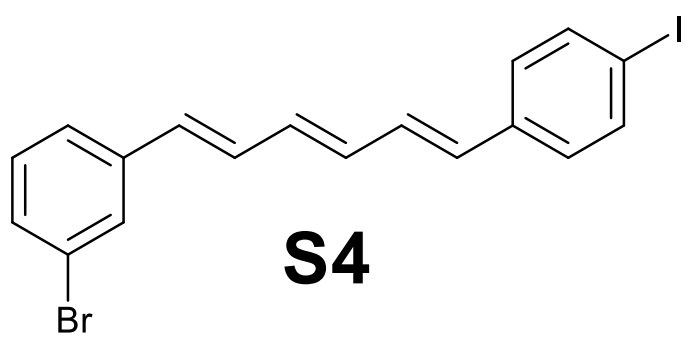


**S4**

**$^1$H NMR** (400 MHz, $CDCl_3$) δ 7.68 – 7.61 (m, 2H), 7.56 (t, *J* = 1.8 Hz, 1H), 7.33 (ddt, *J* = 10.8, 7.9, 1.6 Hz, 2H), 7.23 – 7.11 (m, 3H), 6.93 – 6.80 (m, 2H), 6.58 – 6.44 (m, 4H).

**4.5. Synthesis of ((4-((1E,3E,5E)-6-(3-bromophenyl)hexa-1,3,5-trien-1-yl)phenyl)ethynyl)triisopropylsilane (S5):**

To **S4** (850 mg, 1.95 mmol, 1 eq.), CuI (7 mg, 0.04 mmol, 0.02 eq.), Ph(PPh3)4 (45 mg, 0.04 mmol, 0.02 eq.) was added dry toluene (60 mL) and triethylamine (14 mL). To this solution (Triisopropylsilyl)acetylene (462 mg, 2.54 mmol, 1.3 eq.) was added dropwise. The reaction was heated to 50 °C overnight, then concentrated in vacuo and the residue dry loaded onto silica and purified *via* column chromatography (eluent: n-hexane:DCM 80:20 v/v). The fractions containing product were combined and concentrated *in vacuo,* to give the product a bright yellow solid (786 mg, 82%).

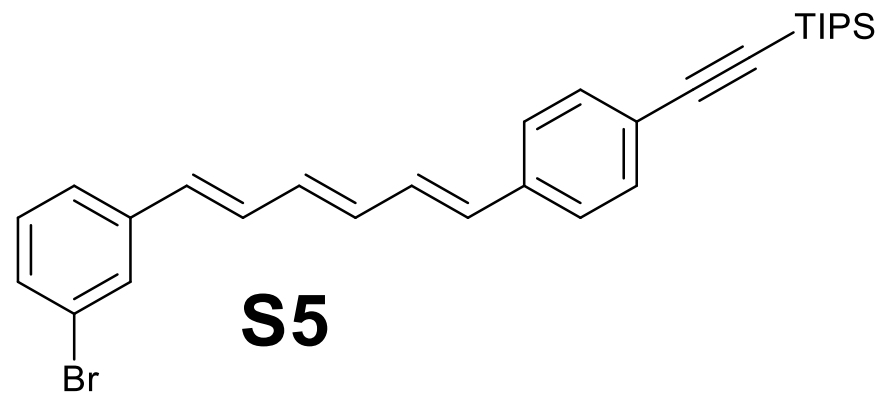

**S5**

**$^{1}$H NMR** (400 MHz, $CDCl_3$) δ 7.56 (t, *J* = 1.8 Hz, 1H), 7.42 (d, *J* = 8.4 Hz, 2H), 7.39 – 7.28 (m, 4H), 7.18 (t, *J* = 7.8 Hz, 1H), 6.94 – 6.81 (m, 2H), 6.65 – 6.44 (m, 4H), 1.13 (s, 21H).

**4.6. Synthesis of triisopropyl((4-((1E,3E,5E)-6-(3-(4,4,5,5-tetramethyl-1,3,2-dioxaborolan-2-yl)phenyl)hexa-1,3,5-trien-1-yl)phenyl)ethynyl)silane (S6):**

**S5** (780 mg, 1.59 mmol, 1.0 eq.), bis(pinacolato)diboron (484 mg, 1.90 mmol, 1.2 eq.), Pd(dppf)Cl2 (37 mg, 0.05 mmol, 0.03 eq.), potassium acetate (421 mg, 4.29 mmol, 2.7 eq.) and stir bar were added to a Schlenk flask, which was evacuated and backfilled with argon 5 times. Dry dioxane (40 mL) was added, the reaction mixture was lowered into a preheated oil bath and stirred at 90 °C in the dark for 24 h. The reaction mixture was allowed to cool to RT and then the solvent removed in vacuo. The crude material was dissolved in DCM, dry loaded onto silica and purified by flash column chromatography (eluent: DCM/ n-hexane 30:70 v/v). After removal of the solvent, the material was sonicated in MeOH, filtered and dried under suction to obtain the product as a yellow powder (490 mg, 57%).

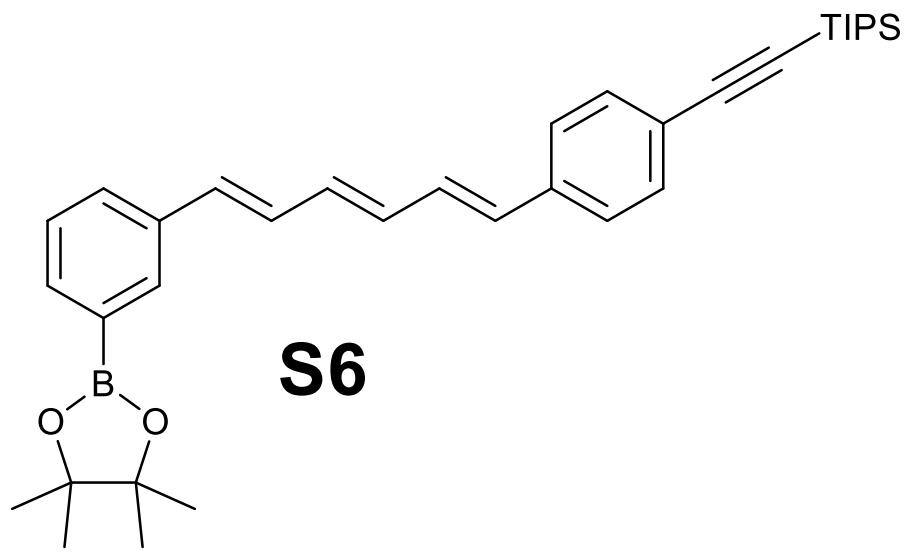

**S6**

**$^{1}$H NMR** (400 MHz, $CDCl_3$) δ 7.87 (s, 1H), 7.67 (d, *J* = 7.3 Hz, 1H), 7.51 (d, *J* = 7.8 Hz, 1H), 7.42 (d, *J* = 8.1 Hz, 2H), 7.33 (dd, *J* = 8.0, 6.4 Hz, 3H), 7.04 – 6.84 (m, 2H), 6.71 – 6.45 (m, 4H), 1.36 (s, 12H), 1.13 (s, 21H).

**4.7. Synthesis of ((4-((1E,3E,5E)-6-(3'-((1E,3E,5E)-6-(4-(2-ethylhexyl)phenyl)hexa-1,3,5-trien-1-yl)-[1,1'-biphenyl]-3-yl)hexa-1,3,5-trien-1-yl)phenyl)ethynyl)triisopropylsilane (PT-DPH$_2$):**

A 10 mL microwave vial was charged with a stir bar, **S5** (57 mg, 0.12 mmol, 1.0 eq.), **2-(3-((1E,3E,5E)-6-(4-(2-ethylhexyl)phenyl)hexa-1,3,5-trien-1-yl)phenyl)-4,4,5,5-tetramethyl-1,3,2-dioxaborolane** (60 mg, 0.13 mmol, 1.1 eq.) and Pd(PPh3)4 (7 mg, 0.06 mmol, 5 mol % ) and flushed under argon. THF (2 mL) and an aqueous solution of Na2CO3 (2M, 0.12 mL) were separately degassed and then added to the solid reagents. The reaction mixture was lowered into a preheated oil bath and heated at 65 °C in the dark for 2 days. The reaction mixture was carefully acidified with 1 M HCl (~ 2 mL) with stirring and then diluted with DCM (~50 mL) and brine (~30 mL). The organic layer was separated and washed with brine (~ 30 mL), dried (MgSO4) and the solvent remove in vacuo. The crude material was then purified by flash column

chromatography (eluent: DCM/ n-hexane 1:9 v/v). Following removal of the solvent the columned material was sonicated in methanol and filtered to obtain the product as a yellow powder (26 mg, 27%).

**PT-DPH$_2$**

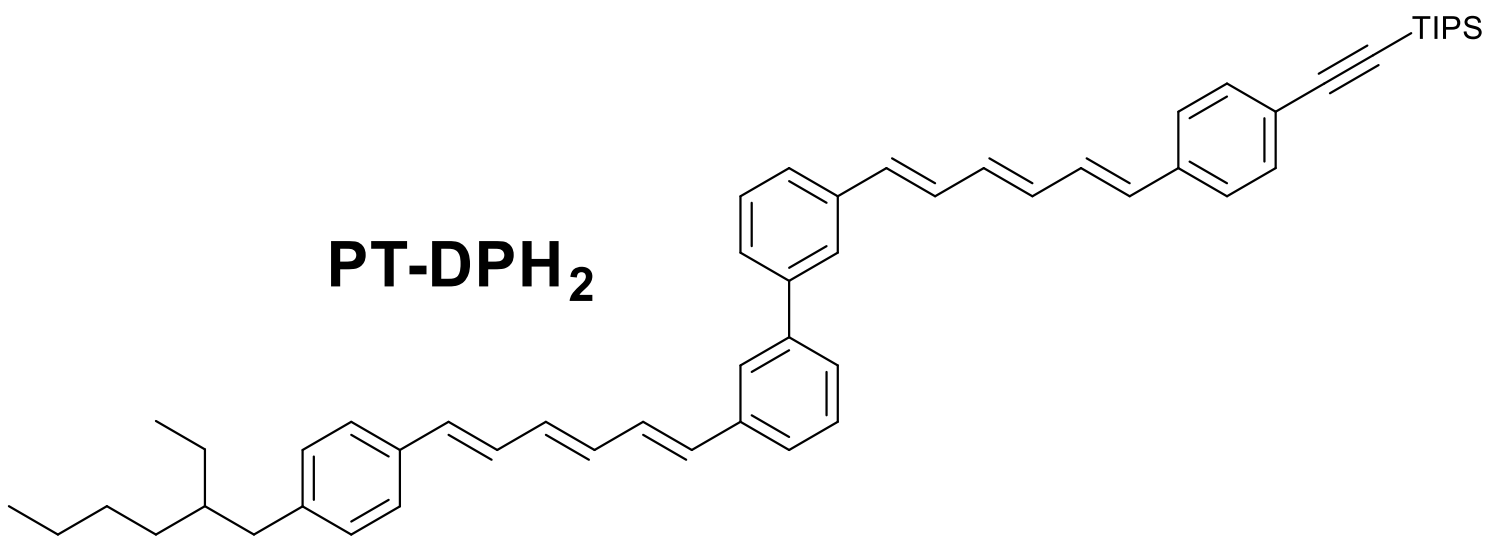


**$^{1}$H NMR** (500 MHz, $CDCl_3$) δ 7.63 (dt, *J* = 3.9, 1.7 Hz, 2H), 7.50 – 7.38 (m, 8H), 7.34 (dd, *J* = 8.3, 6.7 Hz, 4H), 7.11 (d, *J* = 7.9 Hz, 2H), 7.04 – 6.79 (m, 4H), 6.76 – 6.45 (m, 8H), 2.60 – 2.44 (m, 2H), 1.65 – 1.54 (m, 1H), 1.36 – 1.18 (m, 8H), 1.13 (s, 21H), 0.94 – 0.79 (m, 6H).

**$^{13}$C NMR** (126 MHz, $CDCl_3$) δ 141.7, 141.6, 141.5, 138.0, 137.8, 137.3, 134.7, 134.2, 134.2, 133.6, 133.0, 132.9, 132.9, 132.3, 132.1, 132.1, 130.0, 129.7, 129.5, 129.4, 129.1, 129.1, 128.2, 126.6, 126.4, 126.2, 126.1, 125.4, 125.3, 125.3, 125.2, 122.4, 107.4, 91.6, 41.1, 39.9, 32.3, 28.8, 25.4, 23.0, 18.7, 14.1, 11.3, 10.8.

**HRMS**(m/z) Found $[M+H]^+$ = 754.4901 $C_{55}H_{66}Si$ requires 754.4928, Δ = -3.68 ppm

**4.8. Synthesis of triisopropyl((4-((1E,3E,5E)-6-(4'-methyl-[1,1'-biphenyl]-3-yl)hexa-1,3,5-trien-1-yl)phenyl)ethynyl)silane (T-DPH):**

A 10 mL microwave vial was charged with a stir bar, **S5** (100 mg, 0.20 mmol, 1.0 eq.), p-tolyl boronic acid (36 mg, 0.26 mmol, 1.3 eq.) and Pd(PPh3)4 (12 mg, 0.01 mmol, 5 mol % ) and flushed under argon. THF (6 mL) and an aqueous solution of Na2CO3 (2M, 0.6 mL) were separately degassed and then added to the solid reagents. The reaction mixture was lowered into a preheated oil bath and heated at 65 °C in the dark for 2 days. The reaction mixture was carefully acidified with 1 M HCl (~ 2 mL) with stirring and then diluted with DCM (~50 mL) and brine (~30 mL). The organic layer was separated and washed with brine (~ 30 mL), dried (MgSO4) and the solvent remove in vacuo. The crude material was then purified by flash column chromatography (eluent: DCM/ n-hexane 1:9 v/v). Following removal of the solvent the columned material was sonicated in methanol and filtered to obtain the product as a yellow powder (58 mg, 58%).

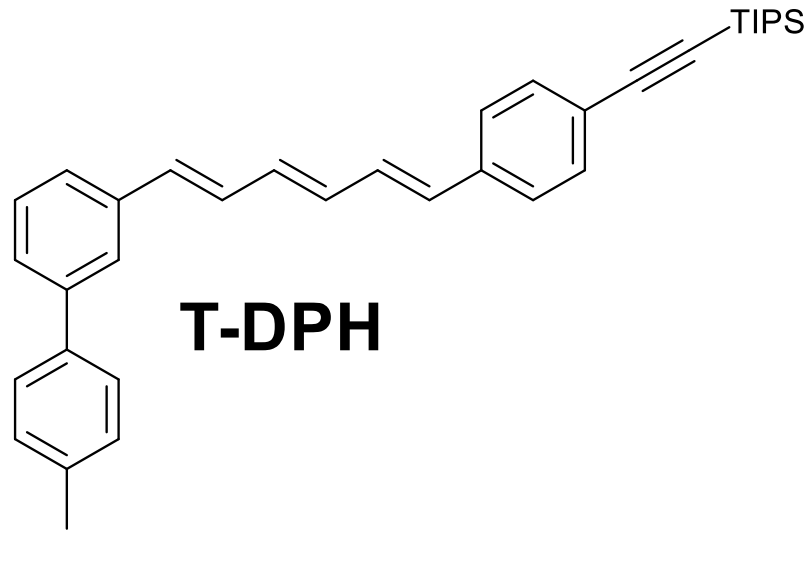


**T-DPH**

**$^{1}$H NMR** (400 MHz, $CDCl_3$) δ 7.61 (s, 1H), 7.51 (d, *J* = 7.9 Hz, 2H), 7.47 – 7.31 (m, 7H), 7.27 (d, *J* = 7.0 Hz, 2H), 7.02 – 6.82 (m, 2H), 6.75 – 6.44 (m, 4H), 2.41 (s, 3H), 1.13 (s, 21H).

**$^{13}$C NMR** (126 MHz, $CDCl_3$) δ 141.8, 138.3, 137.9, 137.5, 137.4, 134.4, 133.6, 133.2, 132.5, 132.2, 130.1, 129.6, 129.5, 129.2, 127.1, 126.5, 126.2, 125.3, 125.2, 122.6, 107.4, 91.8, 21.3, 18.8, 11.5.

**HRMS** (m/z) Found [M+H]+ = 502.3043 $C_{36}H_{42}Si$ requires 502.3050, ∆ = -1.53 ppm

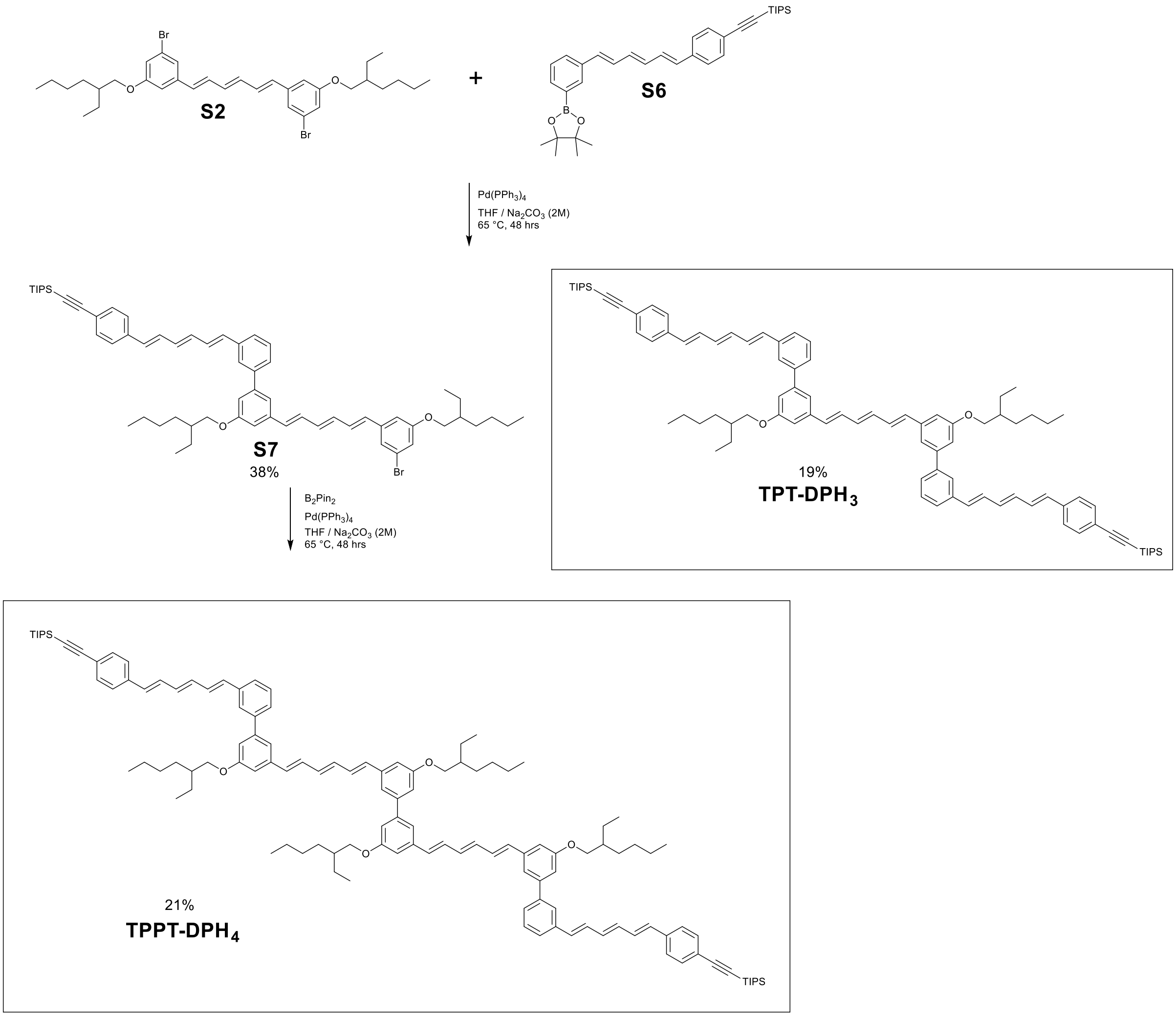


**Scheme S3:** Synthesis of TPT-$DPH_3$ and TPPT-$DPH_4$.

**4.9. Synthesis of (1E,3E,5E)-1,6-bis(5-((2-ethylhexyl)oxy)-3'-((1E,3E,5E)-6-(4((triisopropylsilyl)ethynyl) phenyl) hexa-1,3,5-trien-1-yl)-[1,1'-biphenyl]-3-yl) hexa-1,3,5-triene (TPT-$DPH_3$):**

A 25 mL microwave vial was charged with a stir bar, **S6** (200 mg, 0.37 mmol, 1.0 eq.), **S2** (240 mg, 0.37 mmol, 1.0 eq.), and Pd(PPh3)$_4$ (40 mg, 0.03 mmol, 0.1 eq.) and flushed under argon. THF (11 mL) and an aqueous solution of $Na_2CO_3$ (2M, 1.2 mL) were separately degassed and then added to the solid reagents. The reaction mixture was lowered into a preheated oil bath and heated at 65 °C in the dark for 2 days. The reaction mixture was carefully acidified with 1 M HCl (~ 4 mL) with stirring and then diluted with DCM (~100 mL) and brine (~75 mL). The organic layer was separated and washed with brine (~ 30 mL), dried ($MgSO_4$) and the solvent remove in vacuo. The crude material was then purified by flash column chromatography

(eluent: DCM/ n-hexane gradient from 0:100 to 20:80 v/v) to give two fractions, the first **S7** and the second **TPT-DPH₃**. Following removal of the solvent from the fractions the materials were sonicated in MeOH and the solids filtered to obtain **S7** (140 mg, 38%) and **TPT-DPH₃** (90 mg, 19 %).

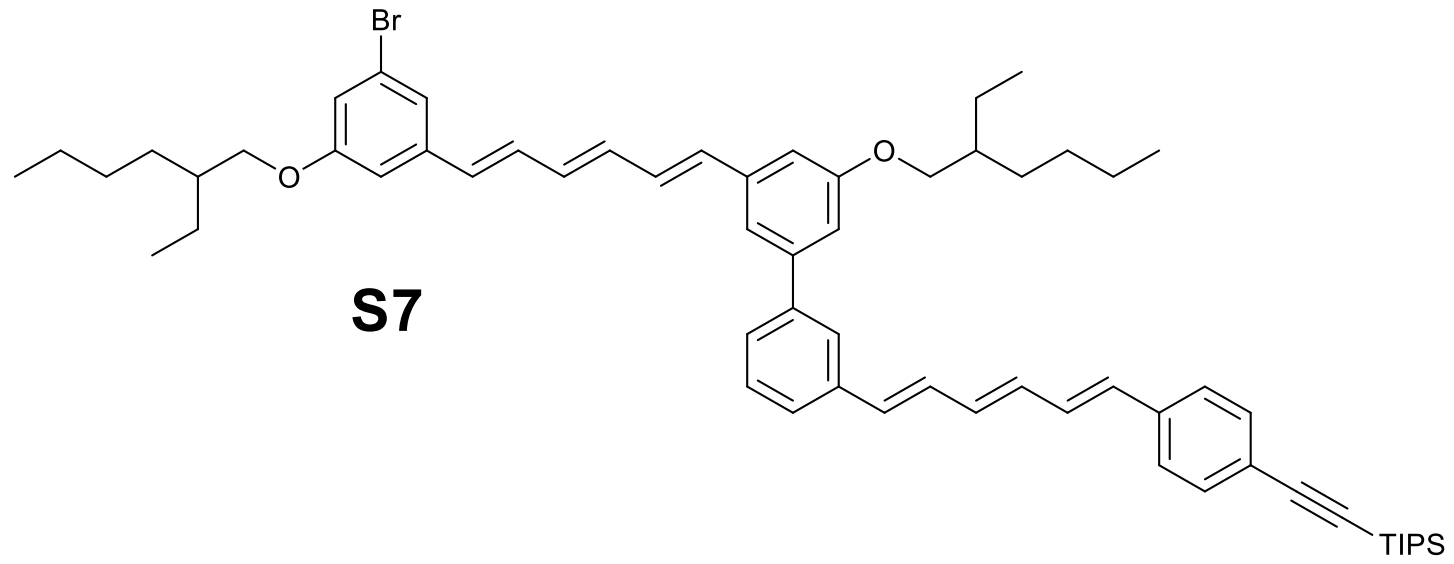


**¹H NMR** (500 MHz, $CD_2Cl_2$) δ 7.67 (s, 1H), 7.56 – 7.34 (m, 7H), 7.26 (s, 1H), 7.16 (s, 1H), 7.09 – 6.85 (m, 8H), 6.78 – 6.46 (m, 8H), 3.95 (dd, *J* = 5.8, 2.6 Hz, 2H), 3.86 (dd, *J* = 5.7, 2.0 Hz, 2H), 1.82 – 1.69 (m, 2H), 1.53 – 1.24 (m, 16H), 1.14 (s, 21H), 1.04 – 0.85 (m, 12H).

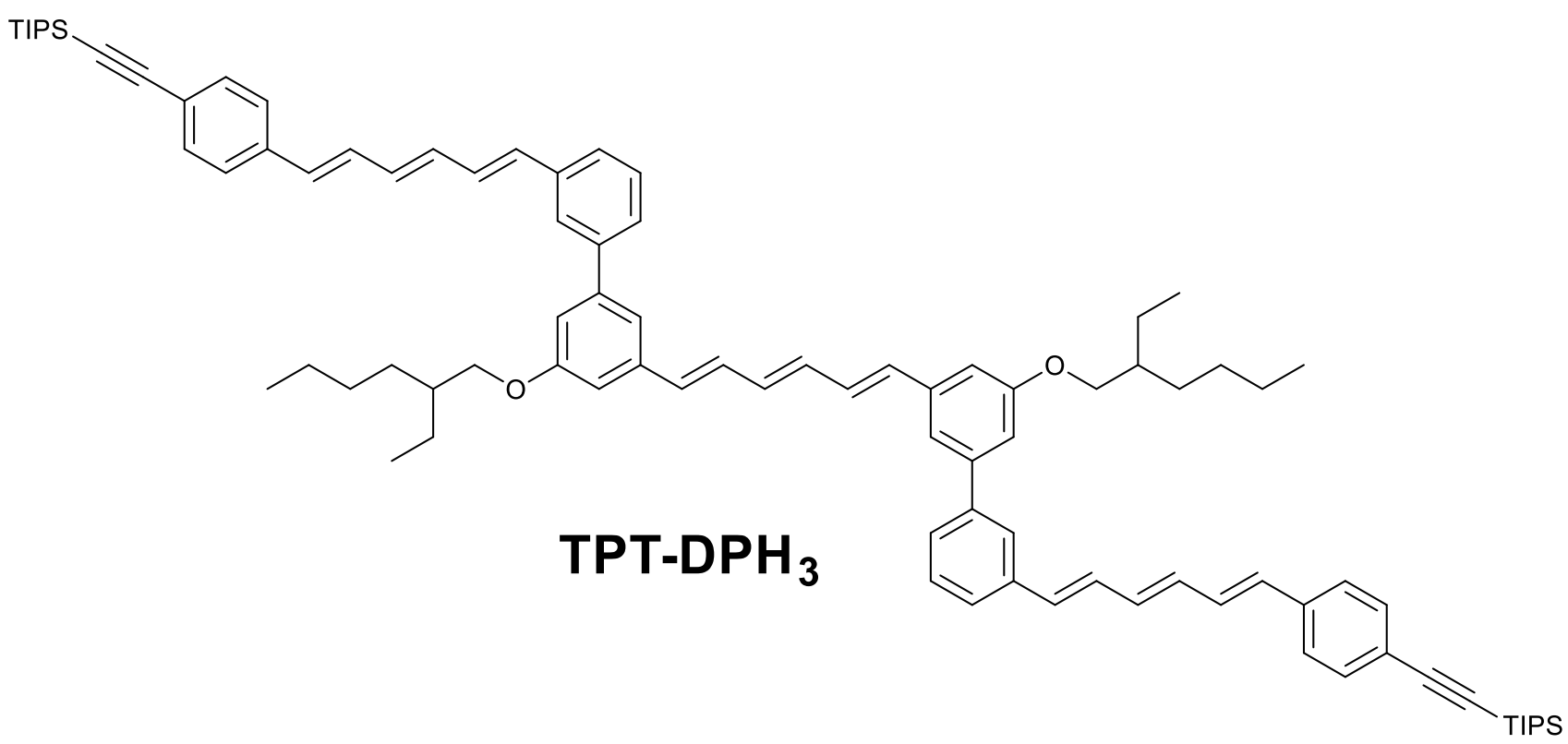


**¹H NMR** (500 MHz, $CD_2Cl_2$) δ 7.67 (t, *J* = 1.8 Hz, 2H), 7.54 – 7.33 (m, 14H), 7.26 (t, *J* = 1.5 Hz, 2H), 7.08 – 6.90 (m, 10H), 6.76 – 6.53 (m, 12H), 4.01 – 3.90 (m, 4H), 1.77 (h, *J* = 6.1 Hz, 2H), 1.54 – 1.30 (m, 16H), 1.14 (s, 42H), 1.00 – 0.88 (m, 12H).

**¹³C NMR** (126 MHz, $CDCl_3$) δ 160.2, 141.8, 139.2, 137.9, 133.8, 133.1, 132.5, 132.2, 130.1, 129.6, 129.2, 126.8, 126.3, 125.5, 122.6, 91.8, 70.8, 39.7, 30.7, 29.3, 24.1, 23.2, 18.8, 14.3, 11.5, 11.3.

HRMS(m/z) Found $[M+H]^+$ = 1308.8539 $C_{92}H_{116}O_2Si_2$ requires 1308.8514, Δ = 1.9 ppm

**4.10. Synthesis of ((((1E,1'E,3E,3'E,5E,5'E)-(((1E,1'E,3E,3'E,5E,5'E)-(5,5'-bis((2-ethylhexyl)oxy)-[1,1'-biphenyl]-3,3'-diyl)bis(hexa-1,3,5-triene-6,1-diyl))bis(5'-((2-ethylhexyl)oxy)-[1,1'-biphenyl]-3',3-diyl))bis(hexa-1,3,5-triene-6,1-diyl))bis(4,1-phenylene))bis(ethyne-2,1-diyl))bis(triisopropylsilane) (TPPT-DPH$_4$):**

A microwave vial was charged with a stir bar, **S7** (130 mg, 0.13 mmol, 1.0 eq.), bis(pinacolato)diboron (17 mg, 0.06 mmol, 0.5 eq.), and Pd(PPh3)4 (15 mg, 0.013 mmol, 0.1 eq.) and flushed under argon. THF (10 mL) and an aqueous solution of Na2CO3 (1M, 1.0 mL) were separately degassed and then added to the solid reagents. The reaction mixture was lowered into a preheated oil bath and heated at 65 °C in the dark for 2 days. The reaction mixture was carefully acidified with 1 M HCl (~ 3 mL) with stirring and then diluted with DCM (~75 mL) and brine (~50 mL). The organic layer was separated and washed with brine (~ 20 mL), dried (MgSO4) and the solvent remove in vacuo. The crude material was then purified by flash column chromatography (eluent: DCM/ n-hexane gradient from 0:100 to 40:60 v/v). Following removal of the solvent the columned material was sonicated in hexane to obtain the product as a yellow powder (23 mg, 21%).

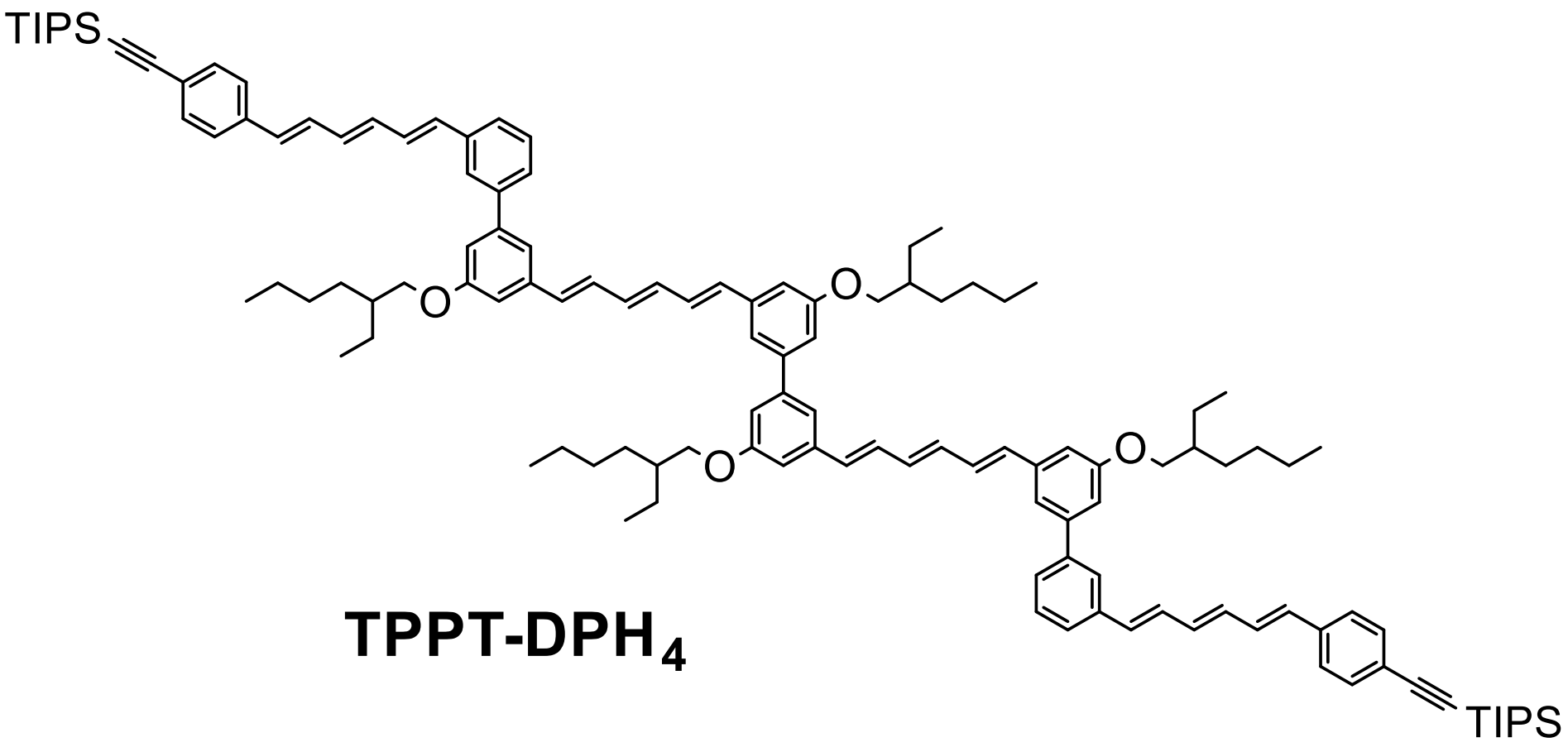


**TPPT-DPH$_4$**

**$^1$H NMR** (500 MHz, $CDCl_3$) δ 7.63 (s, 2H), 7.51 – 7.29 (m, 15H), 7.24 – 7.17 (m, 4H), 7.08 – 6.83 (m, 16H), 6.72 – 6.47 (m, 15H), 4.00 – 3.84 (m, 8H), 1.76 (h, *J* = 6.1 Hz, 4H), 1.53 – 1.28 (m, 32H), 1.13 (s, 42H), 0.99 – 0.88 (m, 24H).

**$^{13}$C NMR** (126 MHz, $CDCl_3$) δ 160.2, 160.1, 143.0, 142.9, 141.8, 139.2, 139.2, 137.9, 137.5, 134.3, 133.9, 133.9, 133.8, 133.1, 132.9, 132.9, 132.5, 132.2, 130.1, 129.8, 129.6, 129.2, 126.8, 126.3, 125.6, 125.5, 122.6, 118.3, 113.3, 113.3, 111.2, 107.4, 91.8, 70.8, 39.7, 39.7, 30.7, 29.9, 29.3, 24.1, 23.2, 18.8, 14.3, 11.5, 11.3.

**HRMS** (m/z) Found [M+H]+ = 1796.2108 $C_{126}H_{163}Si_2O_4$ requires 1796.2084, Δ = 1.28 ppm

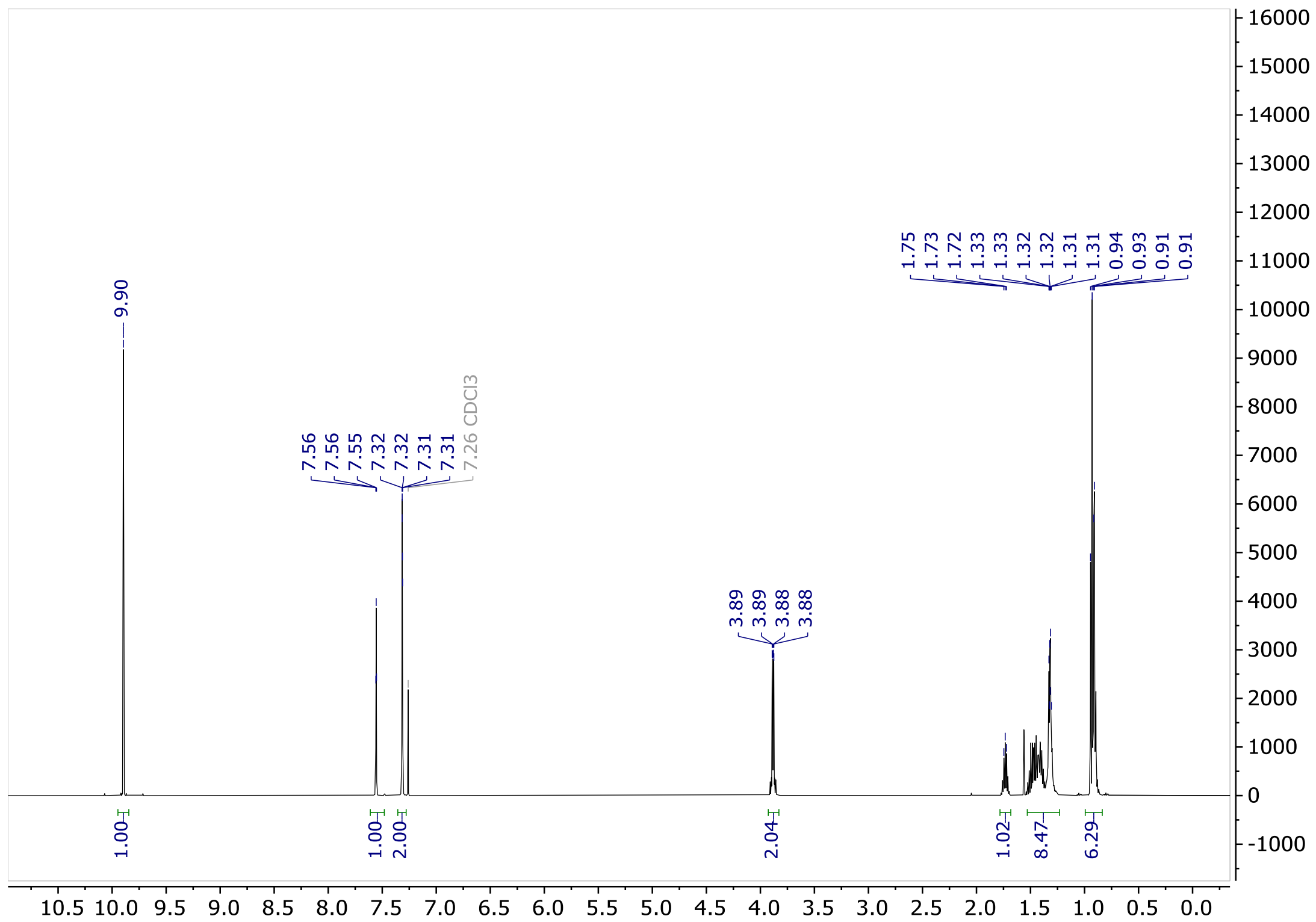

**Figure S21:** 1H NMR (500 MHz, $CDCl_3$) spectra of 3-bromo-5-((2-ethylhexyl)oxy)benzaldehyde (S1).

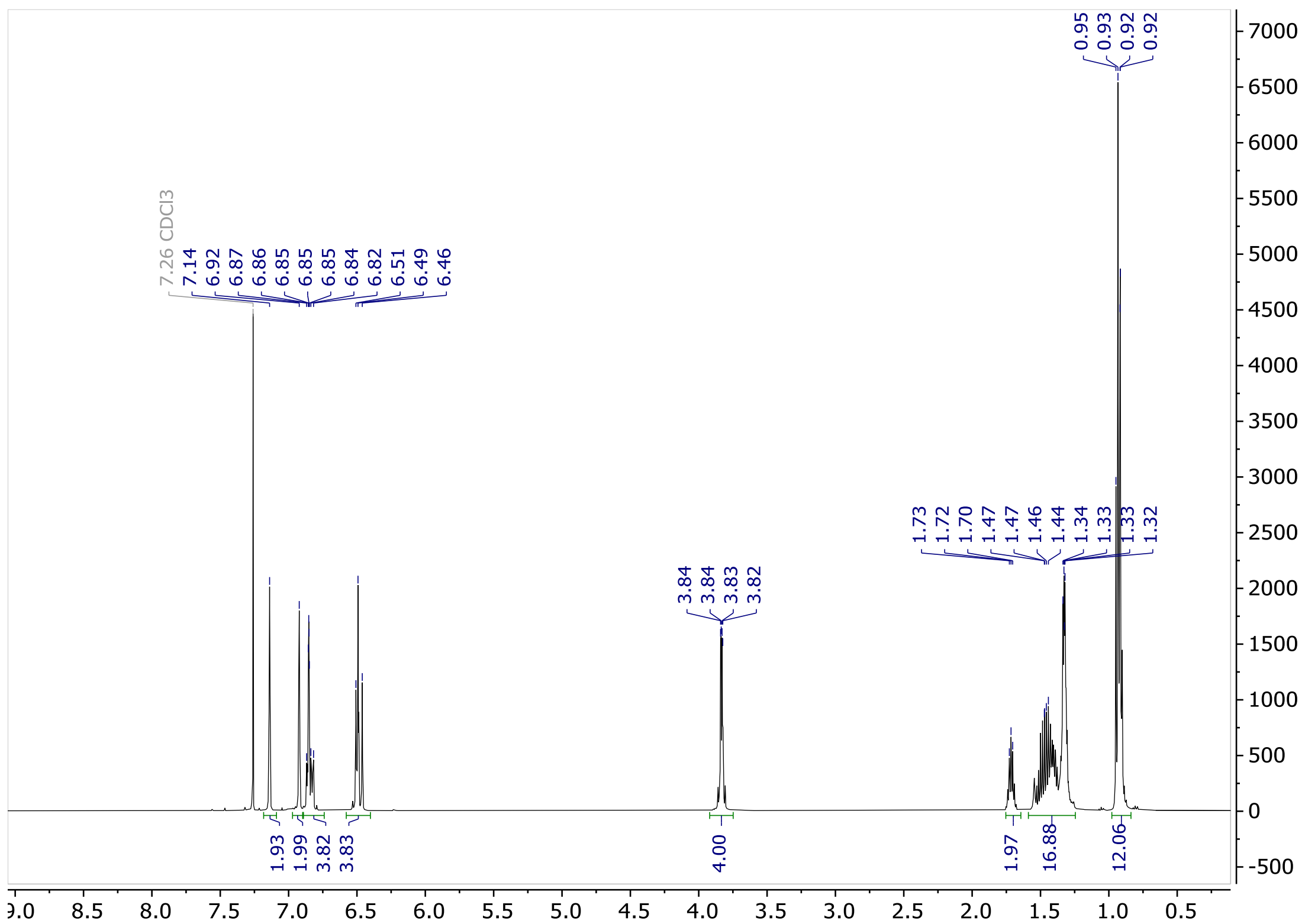

**Figure S22:** 1H NMR (500 MHz, $CDCl_3$) spectra of (1E,3E,5E)-1,6-bis(3-bromo-5-((2-ethylhexyl)oxy)phenyl)hexa-1,3,5-triene (S2).

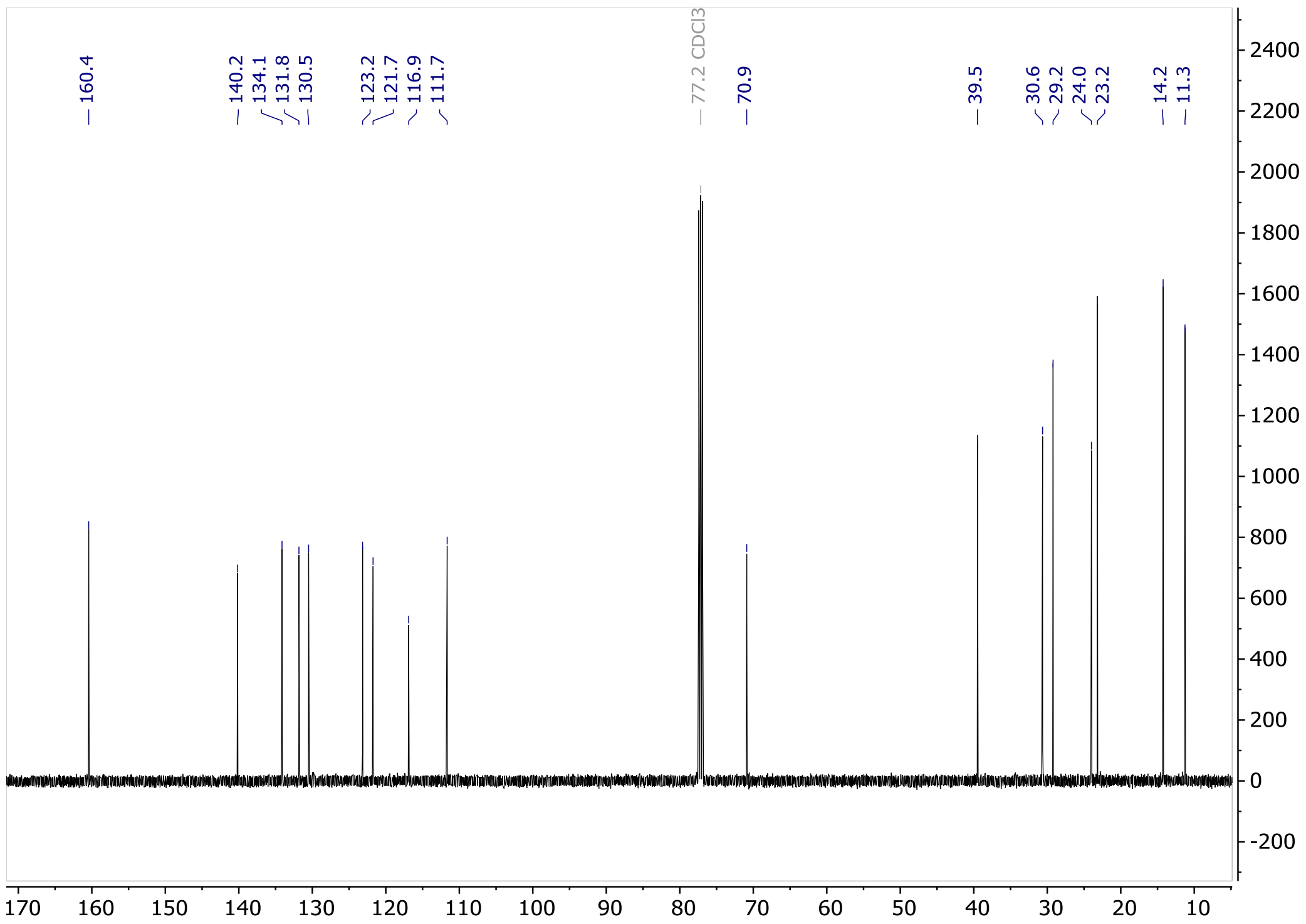


**Figure S23:** 13C NMR (126 MHz, $CDCl_3$) spectra of (1E,3E,5E)-1,6-bis(3-bromo-5-((2-ethylhexyl)oxy)phenyl)hexa-1,3,5-triene (S2).

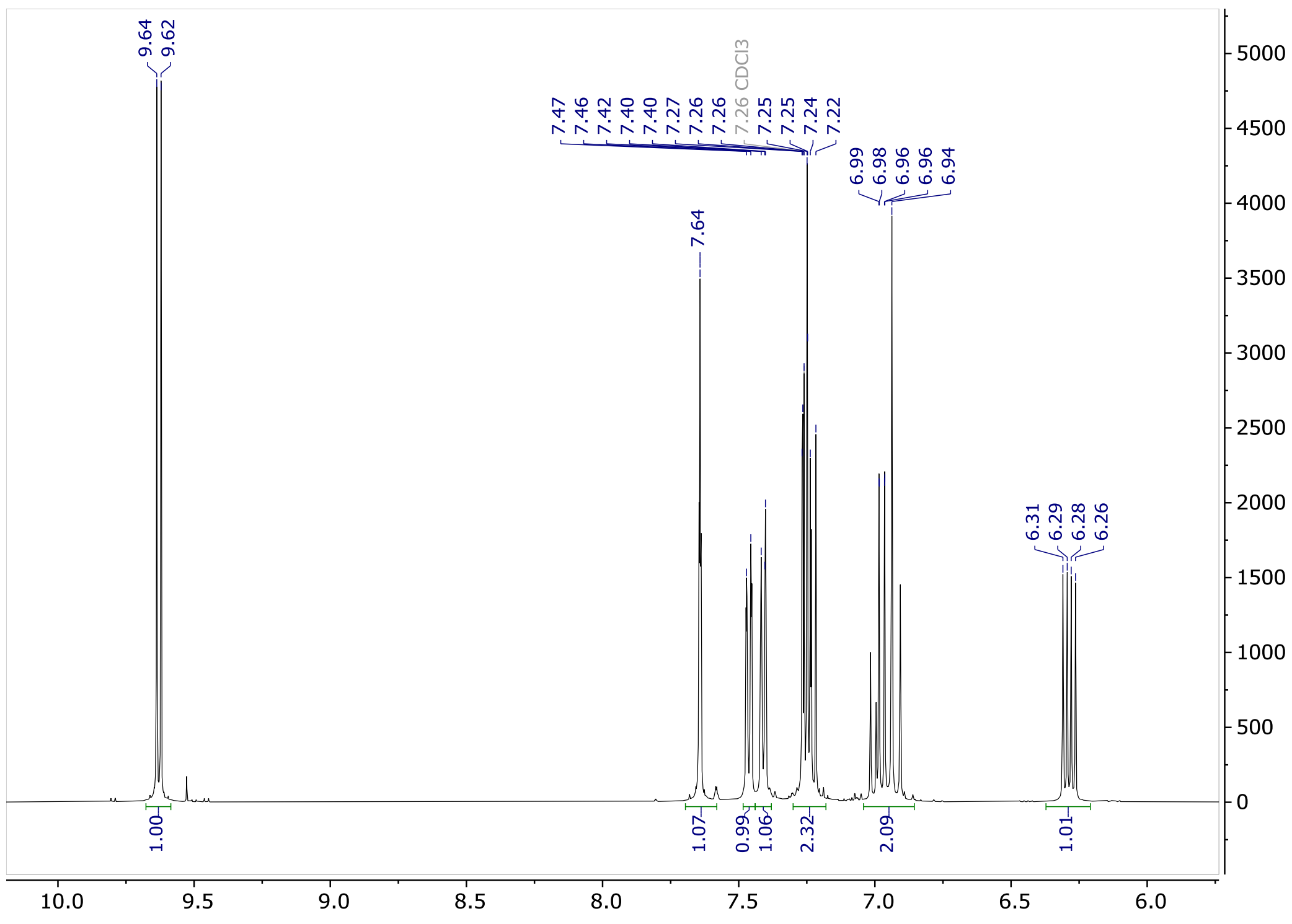


**Figure S24:** 1H NMR (500 MHz, $CDCl_3$) spectra of (2E,4E)-5-(3-bromophenyl)penta-2,4-dienal (S3).

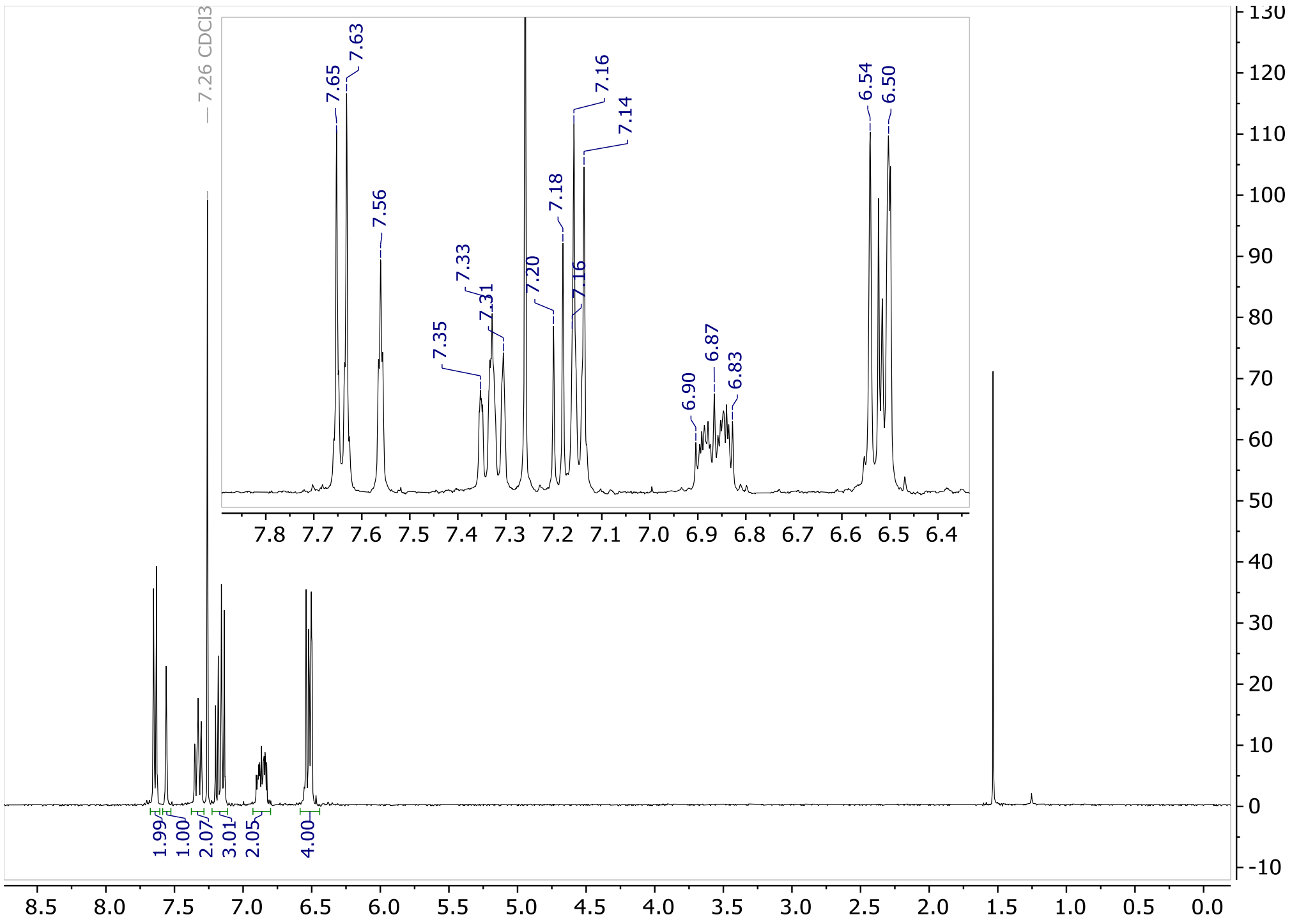

**Figure S25:** 1H NMR (500 MHz, $CDCl_3$) spectra of 1-bromo-3-((1E,3E,5E)-6-(4-iodophenyl)hexa-1,3,5-trien-1-yl)benzene (S4).

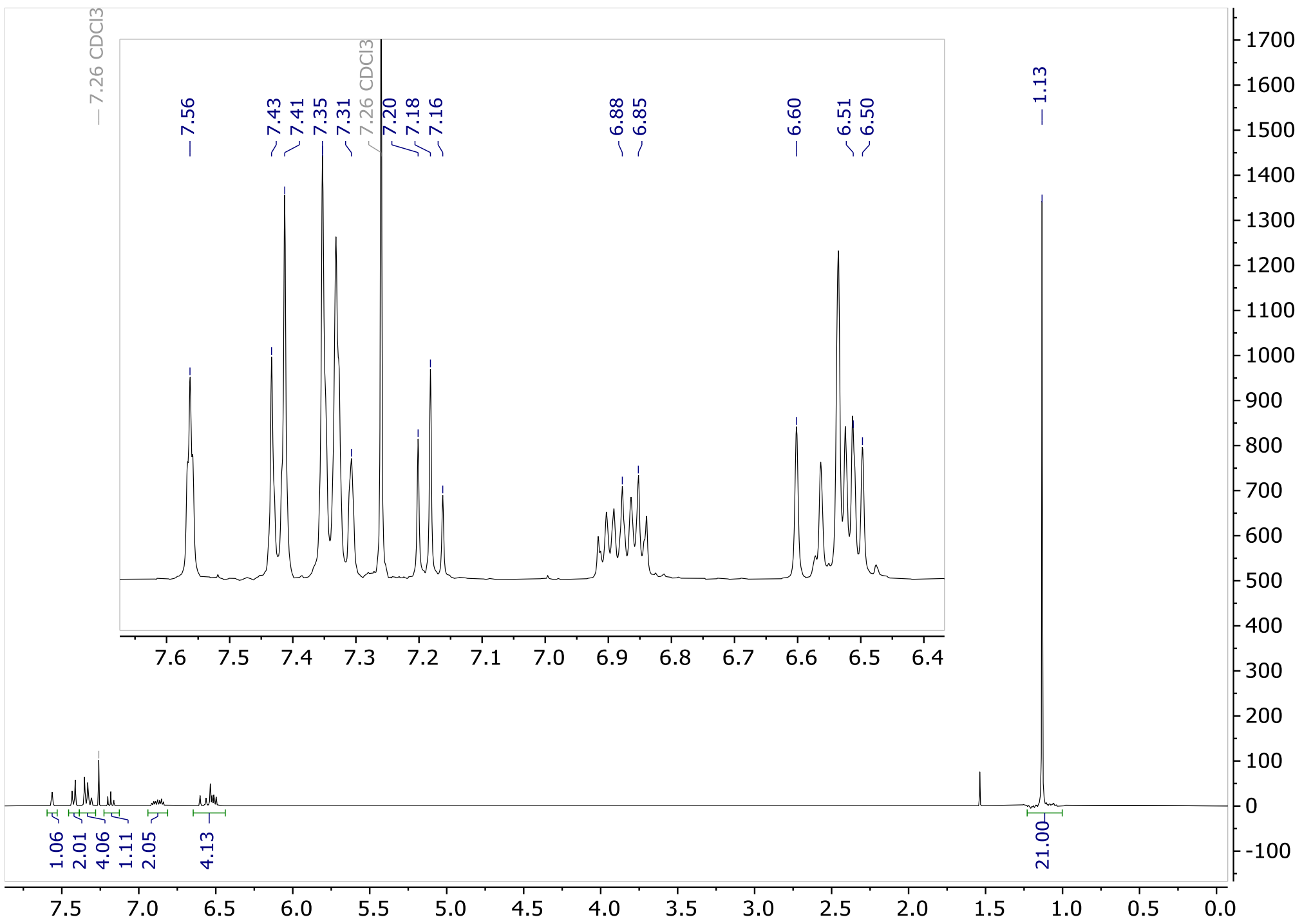

**Figure S26:** 1H NMR (500 MHz, $CDCl_3$) spectra of ((4-((1E,3E,5E)-6-(3-bromophenyl)hexa-1,3,5-trien-1-yl)phenyl)ethynyl)triisopropylsilane (S5).

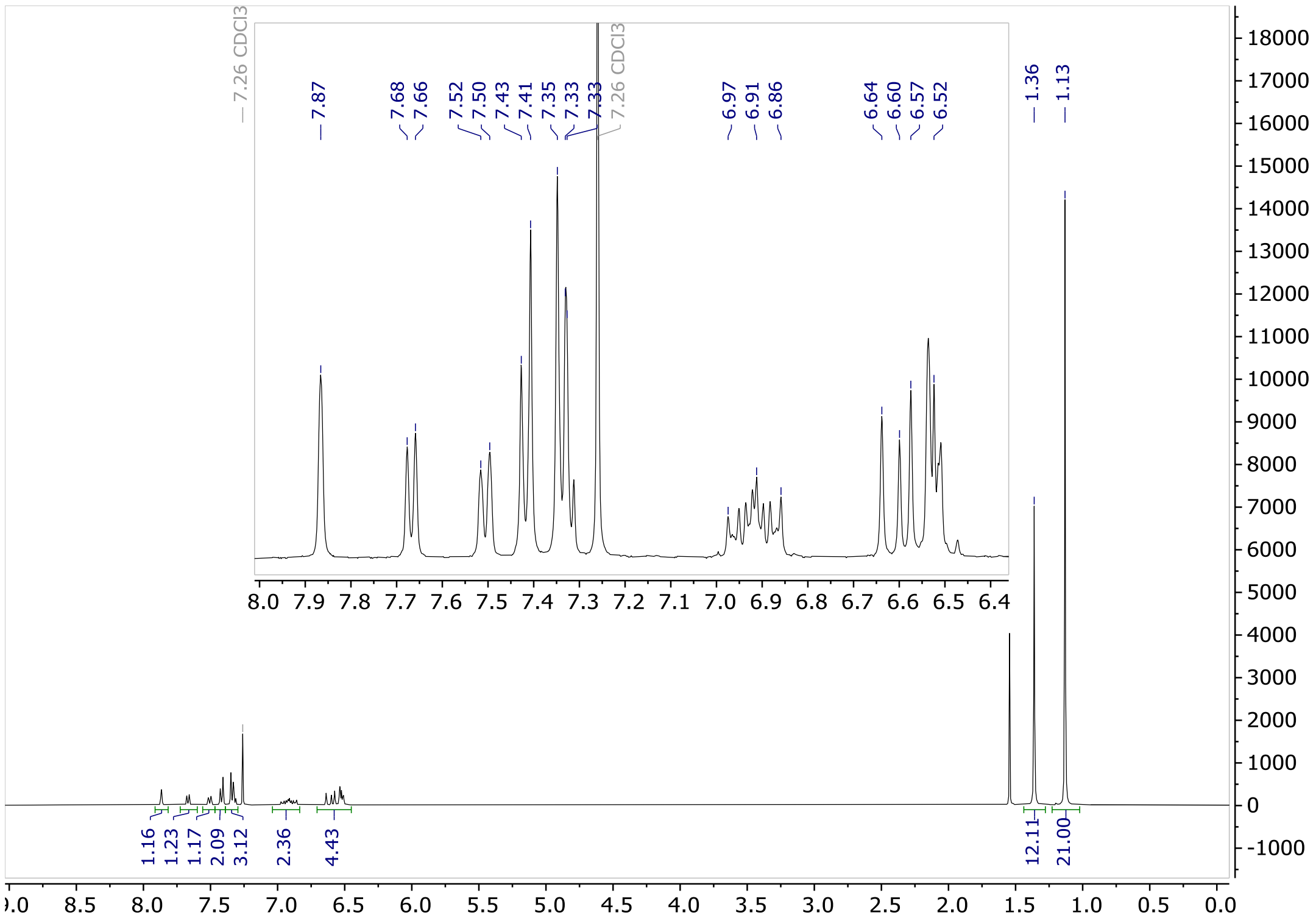

**Figure S27:** 1H NMR (500 MHz, $CDCl_3$) spectra of triisopropyl((4-((1E,3E,5E)-6-(3-(4,4,5,5-tetramethyl-1,3,2-dioxaborolan-2-yl)phenyl)hexa-1,3,5-trien-1-yl)phenyl)ethynyl)silane (S6).

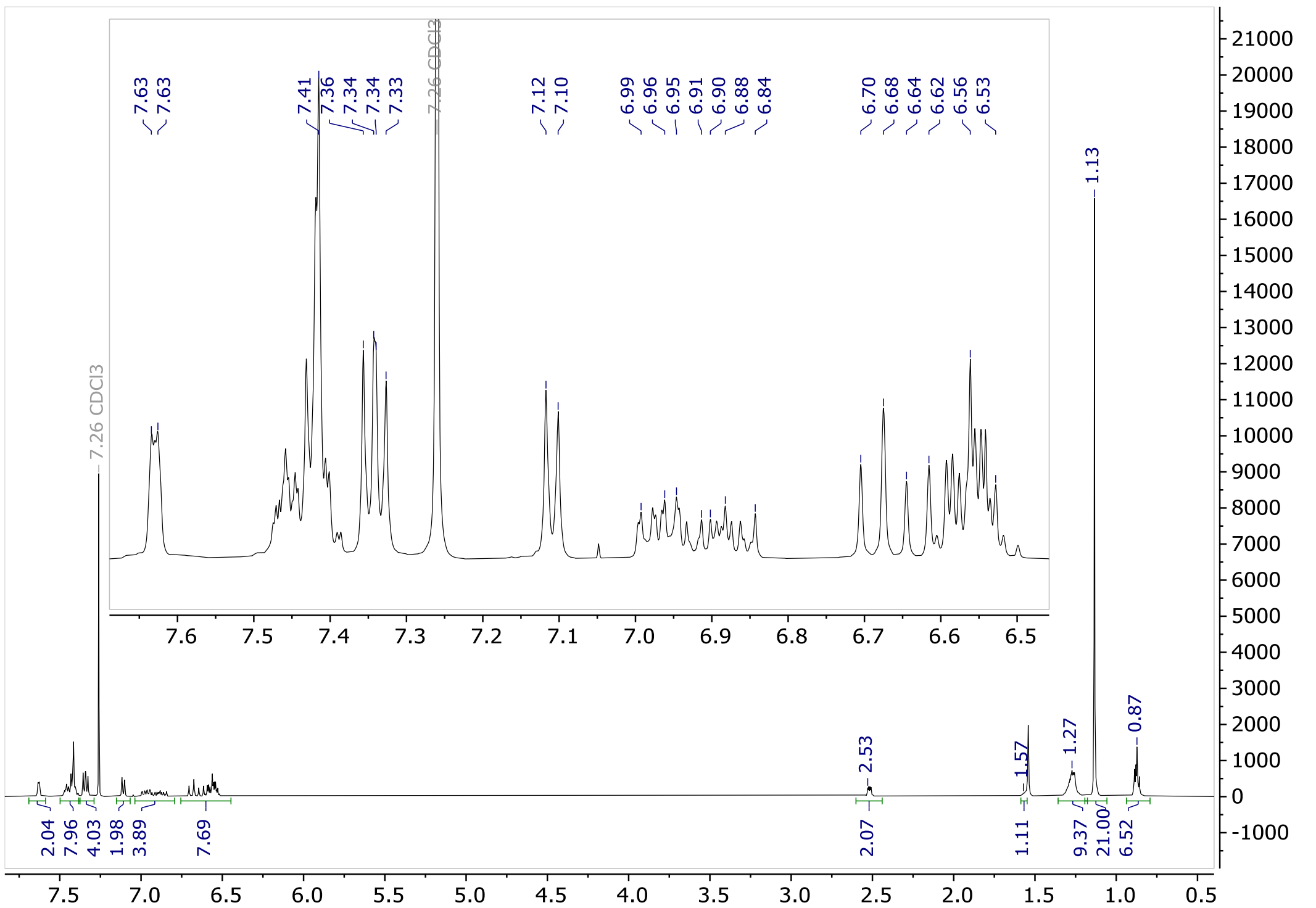

**Figure S28:** 1H NMR (500 MHz, $CDCl_3$) spectra of ((4-((1E,3E,5E)-6-(3'-((1E,3E,5E)-6-(4-(2-ethylhexyl)phenyl)hexa-1,3,5-trien-1-yl)-[1,1'-biphenyl]-3-yl)hexa-1,3,5-trien-1-yl)phenyl)ethynyl)triisopropylsilane (PT-DPH$_2$).

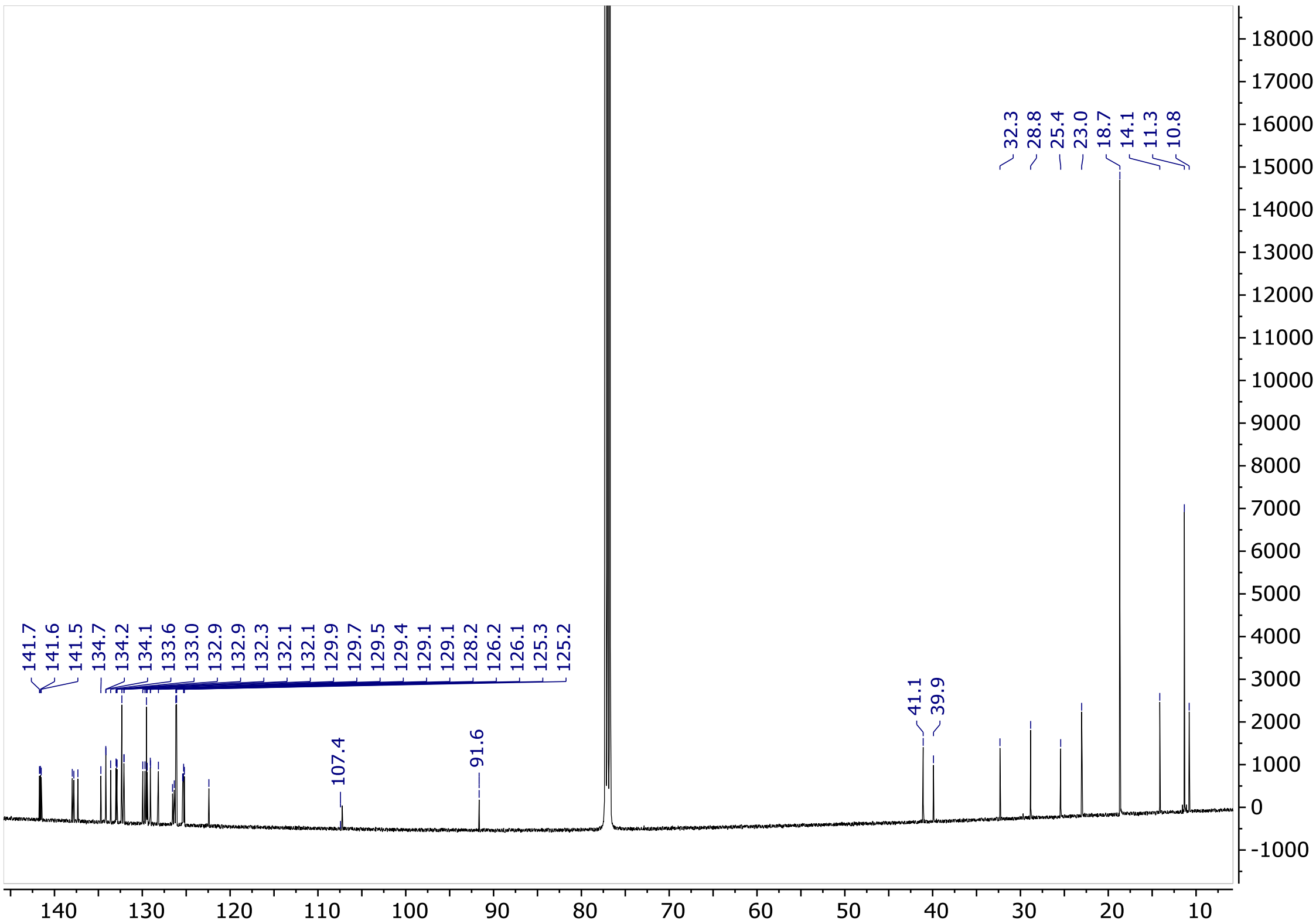

**Figure S29:** 13C NMR (126 MHz, $CDCl_3$) spectra of ((4-((1E,3E,5E)-6-(3'-((1E,3E,5E)-6-(4-(2-ethylhexyl)phenyl)hexa-1,3,5-trien-1-yl)-[1,1'-biphenyl]-3-yl)hexa-1,3,5-trien-1-yl)phenyl)ethynyl)triisopropylsilane (PT-$DPH_2$).

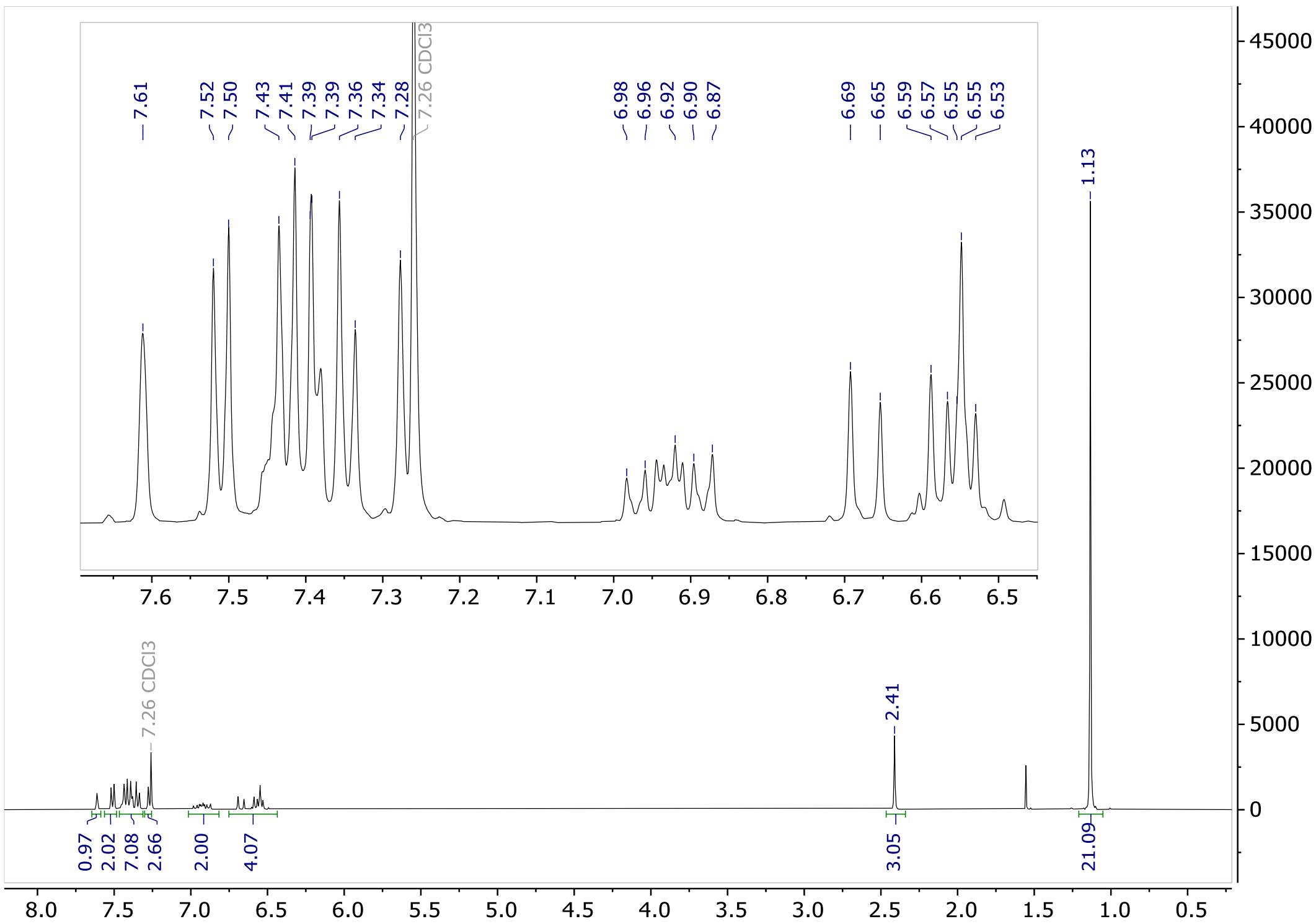

**Figure S30:** 1H NMR (500 MHz, $CDCl_3$) spectra of triisopropyl((4-((1E,3E,5E)-6-(4'-methyl-[1,1'-biphenyl]-3-yl)hexa-1,3,5-trien-1-yl)phenyl)ethynyl)silane (T-DPH).

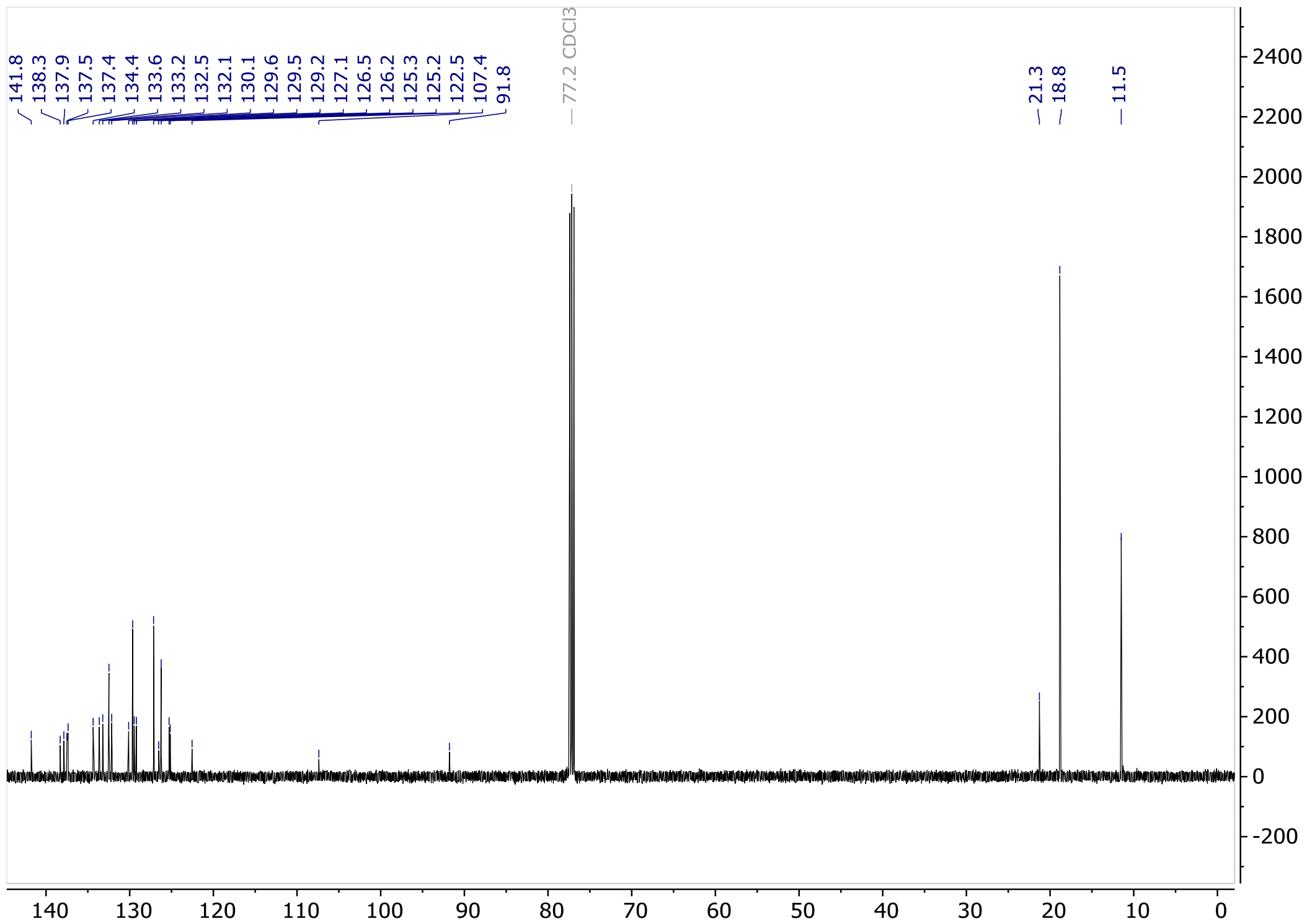


**Figure S31:** 13C NMR (126 MHz, $CDCl_3$) spectra of triisopropyl((4-((1E,3E,5E)-6-(4'-methyl-[1,1'-biphenyl]-3-yl)hexa-1,3,5-trien-1-yl)phenyl)ethynyl)silane (T-DPH).

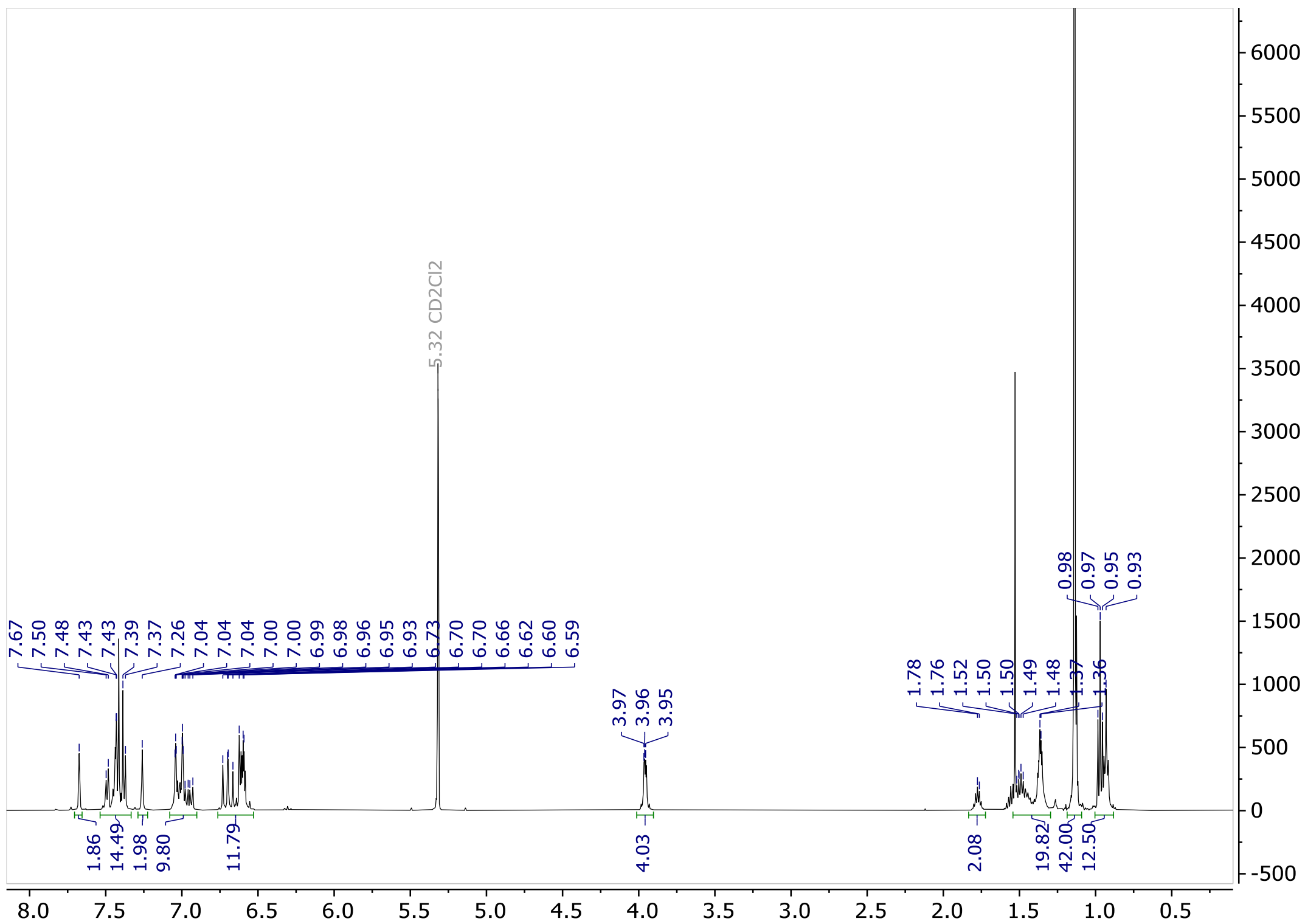


**Figure S32:** 1H NMR (500 MHz, $CDCl_3$) spectra of (1E,3E,5E)-1,6-bis(5-((2-ethylhexyl)oxy)-3'-((1E,3E,5E)-6-(4-((triisopropylsilyl)ethynyl)phenyl)hexa-1,3,5-trien-1-yl)-[1,1'-biphenyl]-3-yl)hexa-1,3,5-triene (TPT-DPH3).

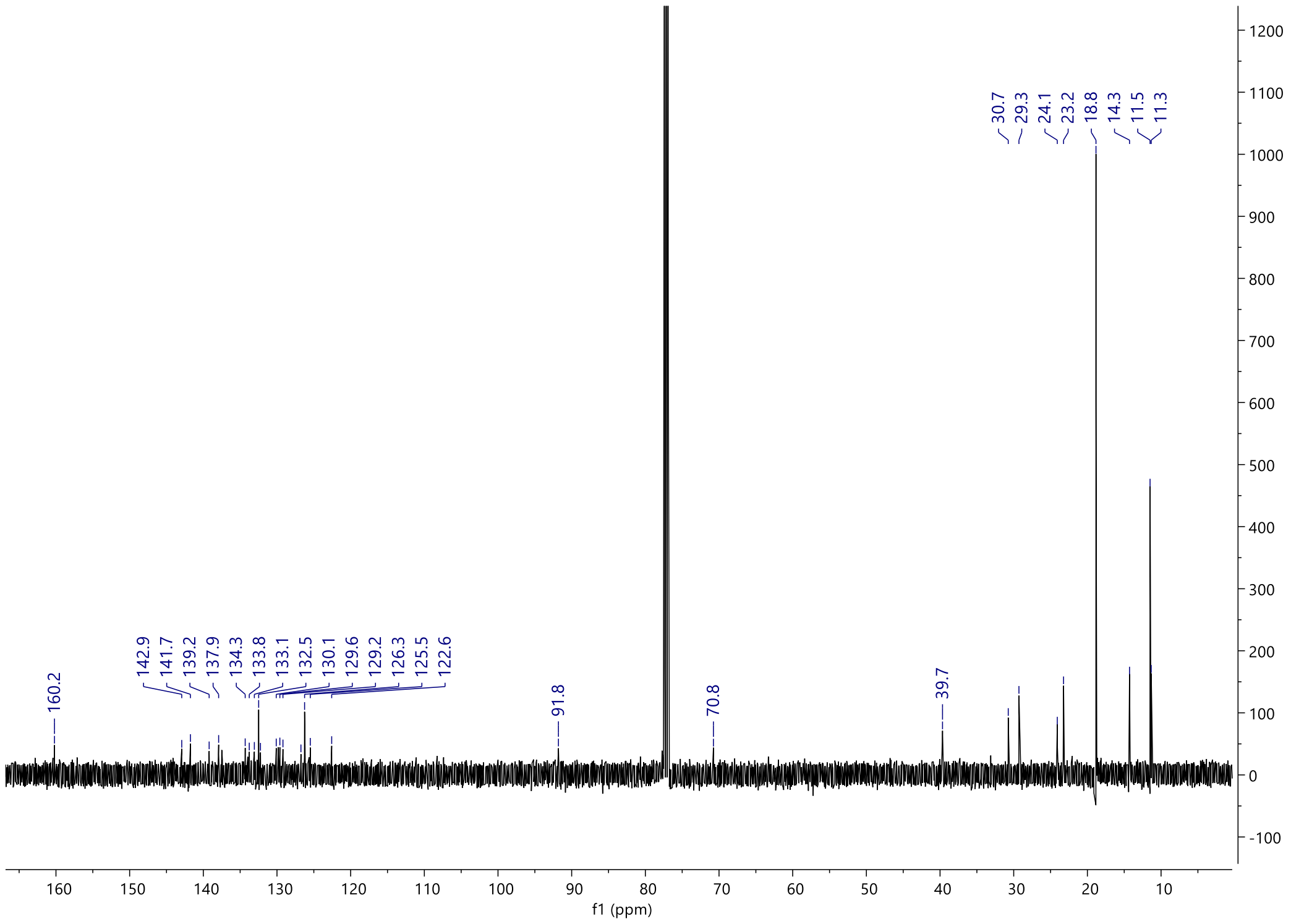

**Figure S33:** 13C NMR (126 MHz, $CDCl_3$) spectra of (1E,3E,5E)-1,6-bis(5-((2-ethylhexyl)oxy)-3'-((1E,3E,5E)-6-(4-((triisopropylsilyl)ethynyl)phenyl)hexa-1,3,5-trien-1-yl)-[1,1'-biphenyl]-3-yl)hexa-1,3,5-triene (TPT-DPH3).

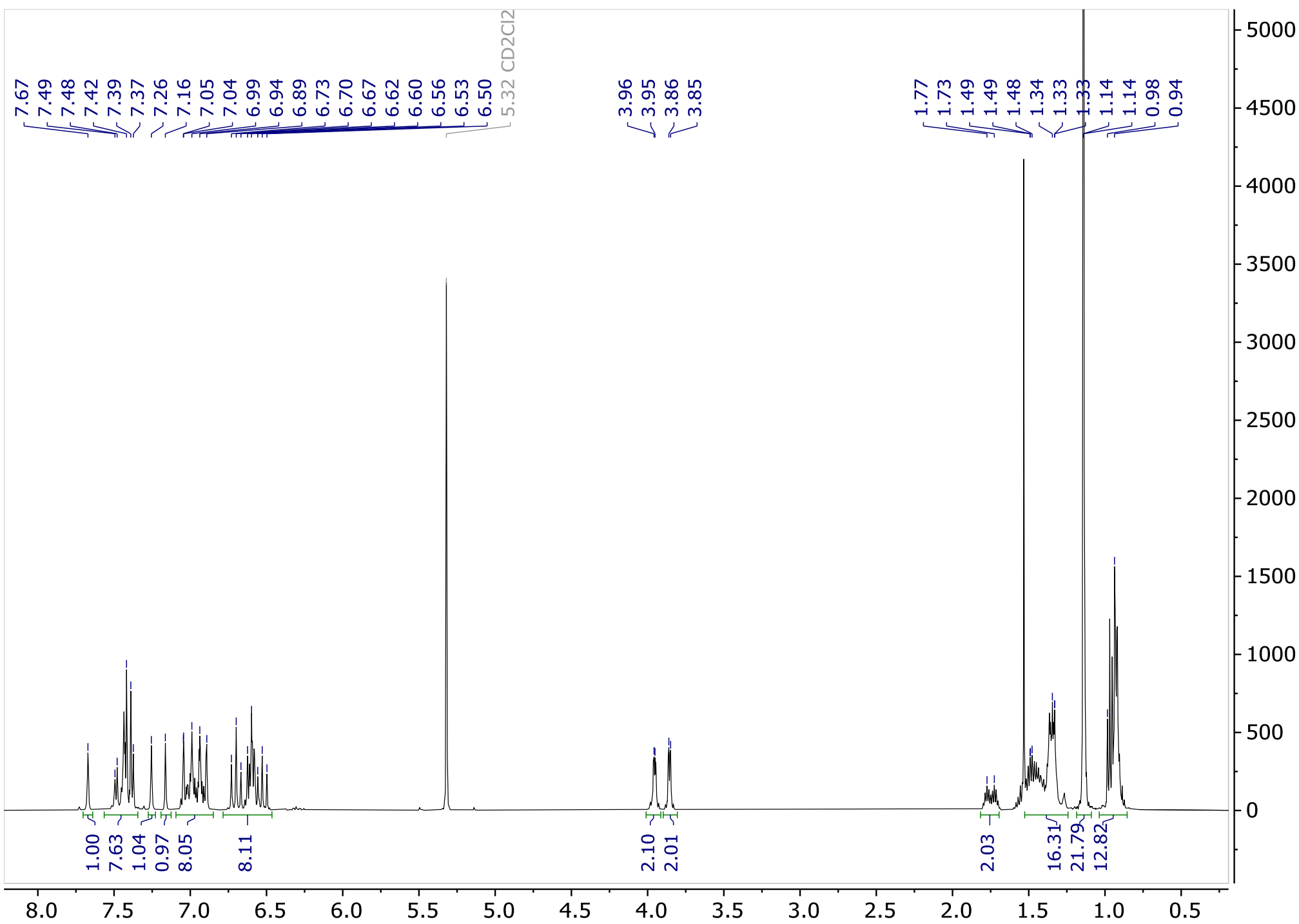

**Figure S34:** 1H NMR (500 MHz, $CDCl_3$) spectra of ((4-((1E,3E,5E)-6-(3'-((1E,3E,5E)-6-(3-bromo-5-((2-ethylhexyl)oxy)phenyl)hexa-1,3,5-trien-1-yl)-5'-((2-ethylhexyl)oxy)-[1,1'-biphenyl]-3-yl)hexa-1,3,5-trien-1-yl)phenyl)ethynyl)triisopropylsilane (S7).

# References


(1) *Citation | Gaussian.com*. https://gaussian.com/citation/ (accessed 2025-08-01).

(2) Eich, ll M.; Beisinghoff, H.; Knodler Ohl, R. M.; Sprave, M.; Vydra, J.; Eckl, M.; Dorr, M.; Zentel, R.; Ahlheim, M.; Stahelin, M.; Zysset Lidng R Levenson J Zyss, B. J.; John de Mello, B. C.; Felix Wittmann, H. An Improved Experimental Determination of External Photoluminescence Quantum Efficiency. *Advanced Materials* **1997**, *9* (3), 230–232. https://doi.org/10.1002/ADMA.19970090308.

(3) Van Stokkum, I. H. M.; Larsen, D. S.; Van Grondelle, R. Global and Target Analysis of Time-Resolved Spectra. *Biochimica et Biophysica Acta (BBA) - Bioenergetics* **2004**, *1657* (2–3), 82–104. https://doi.org/10.1016/J.BBABIO.2004.04.011.

(4) Snellenburg, J. J.; Laptenok, S.; Seger, R.; Mullen, K. M.; van Stokkum, I. H. M. Glotaran: A Java-Based Graphical User Interface for the R Package TIMP. *J. Stat. Softw.* **2012**, *49*, 1–22. https://doi.org/10.18637/JSS.V049.I03.

(5) Millington, O.; Montanaro, S.; Sharma, A.; Dowland, S. A.; Winkel, J.; Grune, J.; Leventis, A.; Bennett, T.; Shaikh, J.; Greenham, N.; Rao, A.; Bronstein, H. The Interplay of Strongly and Weakly Exchange-Coupled Triplet Pairs in Intramolecular Singlet Fission. *J. Am. Chem. Soc.* **2024**, *146* (43), 29664–29674. https://doi.org/10.1021/JACS.4C10483.

(6) Gillin, W. P.; Zhang, S.; Rolfe, N. J.; Desai, P.; Shakya, P.; Drew, A. J.; Kreouzis, T. Determining the Influence of Excited States on Current Transport in Organic Light Emitting Diodes Using Magnetic Field Perturbation. *Phys. Rev. B* **2010**, *82* (19), 195208. https://doi.org/10.1103/PhysRevB.82.195208.

(7) Wohlgenannt, M.; Bobbert, P. A.; Koopmans, B. Intrinsic Magnetic Field Effects in Organic Semiconductors. *MRS Bull.* **2014**, *39* (7), 590–595. https://doi.org/10.1557/MRS.2014.132.

(8) Wong, S. Y.; Benjamin, P.; Hore, P. J. Magnetic Field Effects on Radical Pair Reactions: Estimation of B1/2 for Flavin-Tryptophan Radical Pairs in Cryptochromes. *Physical Chemistry Chemical Physics* **2023**, *25* (2), 975–982. https://doi.org/10.1039/D2CP03793A.

(9) Fallon, K. J.; Sawhney, N.; Toolan, D. T. W.; Sharma, A.; Zeng, W.; Montanaro, S.; Leventis, A.; Dowland, S.; Millington, O.; Congrave, D.; Bond, A.; Friend, R.; Rao, A.; Bronstein, H. Quantitative Singlet Fission in Solution-Processable Dithienohexatrienes. *J. Am. Chem. Soc.* **2022**, *144* (51), 23516–23521. https://doi.org/10.1021/JACS.2C10254.

(10) Millington, O.; Montanaro, S.; Leventis, A.; Sharma, A.; Dowland, S. A.; Sawhney, N.; Fallon, K. J.; Zeng, W.; Congrave, D. G.; Musser, A. J.; Rao, A.; Bronstein, H. Soluble Diphenylhexatriene Dimers for Intramolecular Singlet Fission with High Triplet Energy. *J. Am. Chem. Soc.* **2023**, *145* (4), 2499–2510. https://doi.org/10.1021/JACS.2C12060.
